# Artificial intelligence deciphers codes for color and odor perceptions based on large-scale chemoinformatic data

**DOI:** 10.1093/gigascience/giaa011

**Published:** 2020-02-26

**Authors:** Xiayin Zhang, Kai Zhang, Duoru Lin, Yi Zhu, Chuan Chen, Lin He, Xusen Guo, Kexin Chen, Ruixin Wang, Zhenzhen Liu, Xiaohang Wu, Erping Long, Kai Huang, Zhiqiang He, Xiyang Liu, Haotian Lin

**Affiliations:** 1 State Key Laboratory of Ophthalmology, Zhongshan Ophthalmic Center, Sun Yat-sen University, Xian Lie South Road 54#, Guangzhou 510060, China; 2 School of Computer Science and Technology, Xidian University, Tai Bai South Road 2#, Xi'an 710000, China; 3 Department of Molecular and Cellular Pharmacology, University of Miami Miller School of Medicine, 1120 NW 14th Street, Miami, FL 33136, USA; 4 Sylvester Comprehensive Cancer Center, University of Miami Miller School of Medicine, 1120 NW 14th Street, Miami, FL 33136, USA; 5 Key Laboratory of Machine Intelligence and Advanced Computing, Ministry of Education School of Data and Computer Science, Sun Yat-Sen University, Wai Huan East Road 132#, Guangzhou 510000, China; 6 Key Laboratory of Universal Wireless Communications, Beijing University of Posts and Telecommunications, West Tu Cheng Road 10#, Beijing 100876, China; 7 Center of Precision Medicine, Sun Yat-sen University, Xin Guang West Road 135#, Guangzhou 510080, China

**Keywords:** color perception, odor perception, random forest, deep belief network, physicochemical features

## Abstract

**Background:**

Color vision is the ability to detect, distinguish, and analyze the wavelength distributions of light independent of the total intensity. It mediates the interaction between an organism and its environment from multiple important aspects. However, the physicochemical basis of color coding has not been explored completely, and how color perception is integrated with other sensory input, typically odor, is unclear.

**Results:**

Here, we developed an artificial intelligence platform to train algorithms for distinguishing color and odor based on the large-scale physicochemical features of 1,267 and 598 structurally diverse molecules, respectively. The predictive accuracies achieved using the random forest and deep belief network for the prediction of color were 100% and 95.23% ± 0.40% (mean ± SD), respectively. The predictive accuracies achieved using the random forest and deep belief network for the prediction of odor were 93.40% ± 0.31% and 94.75% ± 0.44% (mean ± SD), respectively. Twenty-four physicochemical features were sufficient for the accurate prediction of color, while 39 physicochemical features were sufficient for the accurate prediction of odor. A positive correlation between the color-coding and odor-coding properties of the molecules was predicted. A group of descriptors was found to interlink prominently in color and odor perceptions.

**Conclusions:**

Our random forest model and deep belief network accurately predicted the colors and odors of structurally diverse molecules. These findings extend our understanding of the molecular and structural basis of color vision and reveal the interrelationship between color and odor perceptions in nature.

## Background

Color vision mediates the relationship between an organism and its environment in multiple important ways, including influencing mate choice, camouflage, and speciation [1]. We see a colorful world because different objects are composed of materials with different reflectance spectra in the wavelength range visible to our eyes [2]. Although knowledge of fundamental optical processes such as reflection, refraction, interference, diffraction, and scattering is accumulating [3], we lack the ability to recognize the color of cellular structure and pattern formation at optical scales from nanometers to microns.

Nature creates various colorful materials based on physicochemical properties including topological and geometrical properties that humans cannot easily see [4, 5]. For instance, the color changes from bright yellow through reddish–purple to blue when the size of a gold sample is decreased [6]. The different colors of disubstituted benzenes were discovered to be related to differences in the molecular structure with *ortho, meta*, and *para* substitutions [7, 8]. The odors of chemicals are also fully encoded within their specific physicochemical properties [9, 10]. The compositions and structures of functional groups have been suggested to be crucial for the perception of aroma [11]. Moreover, evidence of the interaction between color vision and olfaction has been discovered [12]. For example, the odor of a host plant can modify the color sensed by a swallowtail butterfly [13]. The odor of wine can be predicted according to its color [14]. Additionally, the perceived intensity of an odor is positively correlated with the intensity of color [15, 16]. Neuroimaging and repetitive transcranial magnetic stimulation studies have shown that high-level odor processing also actives the visual cortex [17, 18]. However, the relationship between color and odor in terms of molecular physicochemical properties is largely unknown.

Artificial intelligence (AI) tools can be optimized to infer the innate laws of natural processes through machine learning tasks based on large-scale datasets and make predictions of the unknown [19, 20]. In the chemical sciences, AI has been used to guide chemical and material design, synthesis, characterization, and modeling [21, 22]. Previous researchers have equipped AI with a “nose” to predict human olfactory perception from the physicochemical features of 476 molecules and 21 perceptual attributes perceived by 49 individuals [23].

Here, we developed a random forest model and deep belief network (DBN) to predict the colors and odors of chemicals on the basis of their molecular descriptors. We applied random forest and genetic algorithm for feature selection to identify the key physicochemical features that contribute most to the predictive accuracies. In addition, we investigated the connection between the key physicochemical features in color and odor coding to unravel the commonality between visual and olfactory perception.

## Data Description

### Data collection and labeling

A total of 1,267 structurally diverse molecules were used for color prediction in this study, and 598 structurally diverse molecules were used for odor prediction. The color, odor, and 3D structure data of these molecules were all collected from the key chemical information resource at the US NCBI, PubChem [24, 25], between 1 June and 30 November 2017. Molecules with definite colors or odors were defined from PubChem, and molecules with multiple colors or odors that are difficult to define were excluded. The dataset of colors was classified into 12 diverse colors, including yellow (257 molecules), white (301 molecules), orange (31 molecules), red (16 molecules), purple (11 molecules), green (24 molecules), blue (9 molecules), brown (20 molecules), amber (15 molecules), gray (6 molecules), black (17 molecules), and colorless (560 molecules). The dataset of odors was classified into 12 diverse odors, including ammonia (37 molecules), aromatic (36 molecules), characteristic (27 molecules), flower (19 molecules), fruity (29 molecules), mild (38 molecules), other (127 molecules), pleasant (16 molecules), unpleasant (23 molecules), spicy (54 molecules), sweet (30 molecules), and odorless (162 molecules).

### Physicochemical features of the molecules

**Figure 1:**
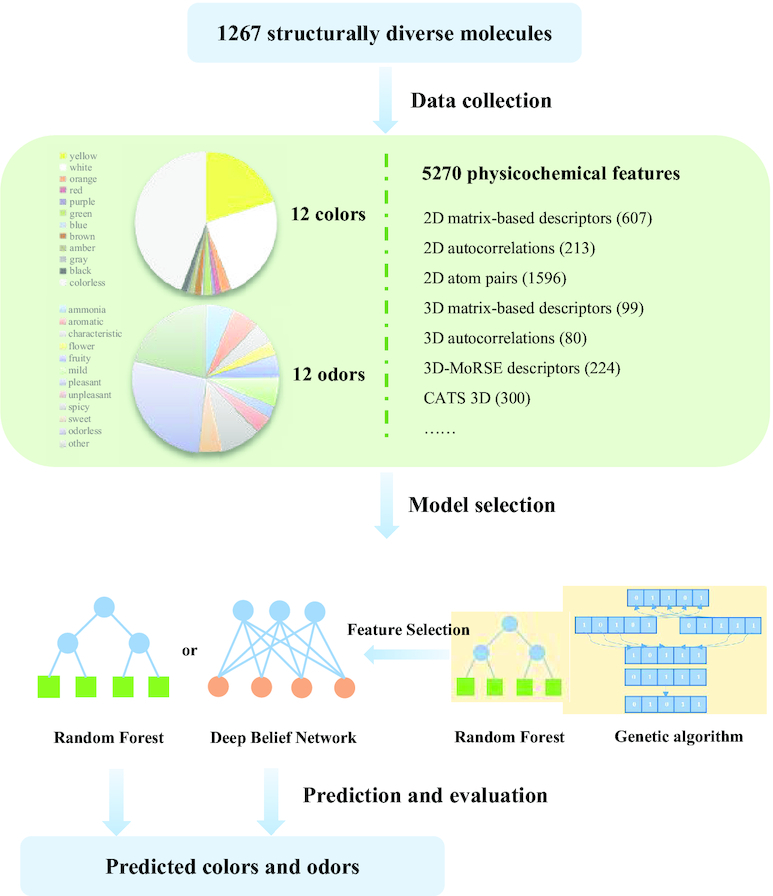
The overall workflow of color prediction and odor prediction. A total of 1,267 structurally diverse molecules were labeled with 12 diverse colors, and 598 structurally diverse molecules were labeled with 12 diverse odors. In addition, 5,270 physicochemical features of each molecule were generated by Dragon. Random forest models and deep belief networks were built to predict colors or odors using their physicochemical features. Feature selection was conducted by random forest models and the genetic algorithm. With the selected feature, random forest models and deep belief networks were reused for color and odor prediction. The models were evaluated on the basis of the means and variances of the accuracies between the labeled and predicted colors or odors.

The PubChem compound identifier for each molecule was provided (Supplementary Data 1–3). We applied the commercial chemoinformatics software package Dragon (version 7.0 [26]) to calculate 5,270 physicochemical descriptors for each of the molecules, including the simplest atom types, functional groups and fragment counts, topological and geometrical descriptors, 3D descriptors, several property estimations (such as log*P*), and drug-like and lead-like alerts (such as the Lipinski alert). These molecular descriptors are formal mathematical representations of a molecule and include their definition, symbols and labels, formulas, some numerical examples, data, and molecular graphs, as presented in the Handbook of Molecular Descriptors [27]. The missing values marked as “NaN” simply mean that for these molecules, some descriptors have not been calculated for some reason, which is common because several descriptors have particular constraints. Molecules with >2,000 descriptors marked as “NaN” were not used. We replaced all of the “NaN” entries with “0” during the dataset preprocessing. For molecules with color, the average number of “NaN” entries within 5,270 descriptors was 353 per molecule. For molecules with odor, the average number of “NaN” entries within 5,270 descriptors was 28 per molecule. The data were divided into the training and testing datasets without oversampling using *k*-fold cross-validations (*k* = 4). The overall workflow is shown in Fig. 1.

## Results

### Color prediction

**Figure 2:**
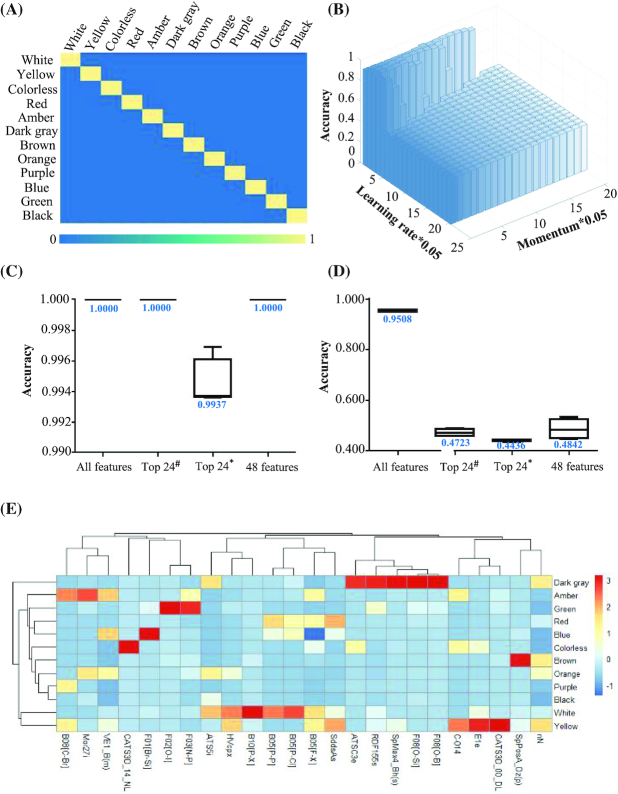
Color prediction using the random forest model and DBN. A. Confusion matrix for the classification of color with 100% accuracy by the random forest. The X-axis presents the labeled colors of the molecules, and the Y-axis presents the predicted colors of the molecules. B. The classification results for color were as high as 95.23% using the DBN. The X-axis presents the learning rate, the Y-axis presents the algorithm parameter “momentum,” and the Z-axis presents the accuracy rate. C. Boxplot presenting the accuracy of color prediction from 4-fold cross-validations using the random forest with all features, the top 24 features selected by random forest models, the top 24 features selected by random forest and the genetic algorithm, and the total 48 features from above. The median values of these boxplots are labeled. D. Boxplot presenting the accuracy of color prediction using the DBN with all features, the top 24 features selected by random forest models, the top 24 features selected by random forest and the genetic algorithm, and the total 48 features from above. The median values of these boxplots are labeled. ^#^Random forest models, *random forest models and genetic algorithm. E. Heat map of the correlation values between the top 24 features selected by random forest models and the 12 colors based on the hierarchical clustering framework. The connections between the colors and descriptors were calculated by the Euclid distances.

Random forest and DBN algorithms were applied for the *in silico* test. Using *k*-fold cross-validations (*k* = 4), the random forest model identified and utilized the most discriminative features with 100% accuracy in the prediction of 12 colors (Fig. 2A and C, Fig. S1), with a κ coefficient of 1. As a type of probability generation model consisting of multiple restricted Boltzmann machines (RBMs), the DBN also performed excellently, with a predictive accuracy of 95.23% ± 0.40% (mean ± SD) (Fig. 2B and D) and a κ coefficient of 0.9400 ± 0.0030 (mean ± SD).

### Key physicochemical features for color perception

Twenty-four descriptors were selected as the key physicochemical features in random forest algorithm with a classification accuracy of 100 by using *k*-fold cross-validations (*k* = 4). The molecular descriptor “B05[F-X]” ranked first, followed by “SddsAs,” “RDF155s,” and “F08[O-Si].” The heat map of the hierarchical cluster analysis between the 24 key features and the 12 colors is shown in Fig. 2E. “B10[P-X],” “B05[P-P],” “B05[P-Cl],” “HVcpx,” and “ATS5i” were the main contributors to white, whereas “CATS3D_00_DL” and “E1e” were the most important features in predicting yellow. Information relevant to the key physicochemical features for color perception is reported in Table S1.

### Distinction and connection with olfaction perception

**Figure 3:**
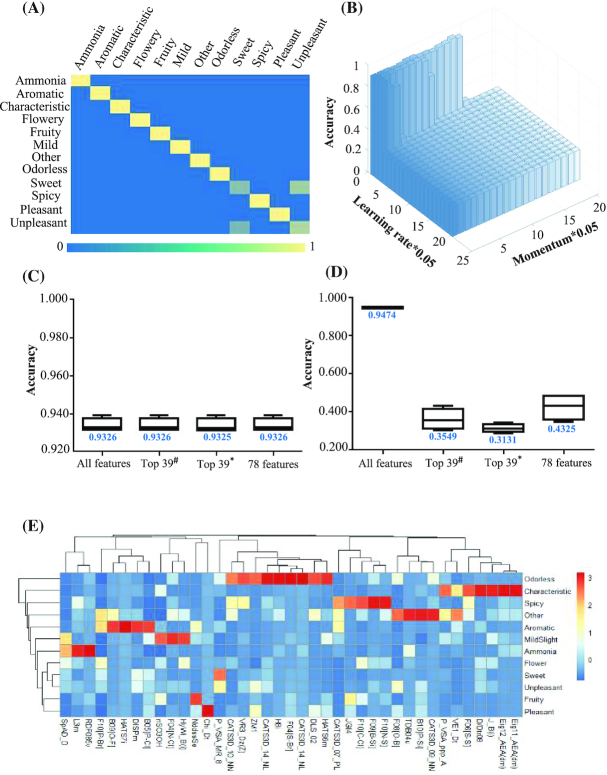
Odor prediction using the random forest model and DBN. A. The confusion matrix for the classification of odor with 93.40% accuracy by the random forest. B. The classification results for odor were as high as 94.75% using the DBN. The *X*-axis presents the learning rate, the *Y*-axis presents the algorithm parameter “momentum,” and the *Z*-axis presents the accuracy rate. C. Boxplot to present the accuracy of color prediction using the random forest with all features, the top 39 features selected by random forest models, the top 39 features selected by the random forest and the genetic algorithm, and the total 78 features from above. The median values of these boxplots are labeled. D. Boxplot presenting the accuracy of color prediction using the DBN with all features, the top 39 features selected by random forest models, the top 39 features selected by random forest and the genetic algorithm, and the total 78 features from above. The median values of these boxplots are labeled. ^#^Random forest models, *random forest models and genetic algorithm. E. Heat map of the correlation values between the top 39 features selected by random forest models and the 12 odors based on the hierarchical clustering framework. Connections between the odors and descriptors were calculated by the Euclid distances.

We next applied the AI platform to predict odor perception on the basis of physicochemical features. In total, 598 structurally diverse molecules were collected and classified into 12 diverse odors based on PubChem [24], including pleasant, unpleasant, ammonia, aromatic, flowery, fruity, spicy, sweet, mild, odorless, characteristic, and other. The accuracies of the odor prediction were 93.40% ± 0.31% for the random forest model using *k*-fold cross-validations (*k* = 4) (Fig. 3A and C, Fig. S1) and 94.75% ± 0.44% for the DBN (Fig. 3B and D), with κ coefficients of 0.9232 ± 0.0037 and 0.9397 ± 0.0031, respectively. Thirty-nine descriptors were selected as the key physicochemical features in the random forest model with a classification accuracy of 93.40% ± 0.31% (Table S3). The heat map of the hierarchical cluster analysis between the 39 key physicochemical features and the 12 odors is shown in Fig. 3E. Information relevant to the key physicochemical features for odor perception is presented in Table S2.

**Figure 4:**
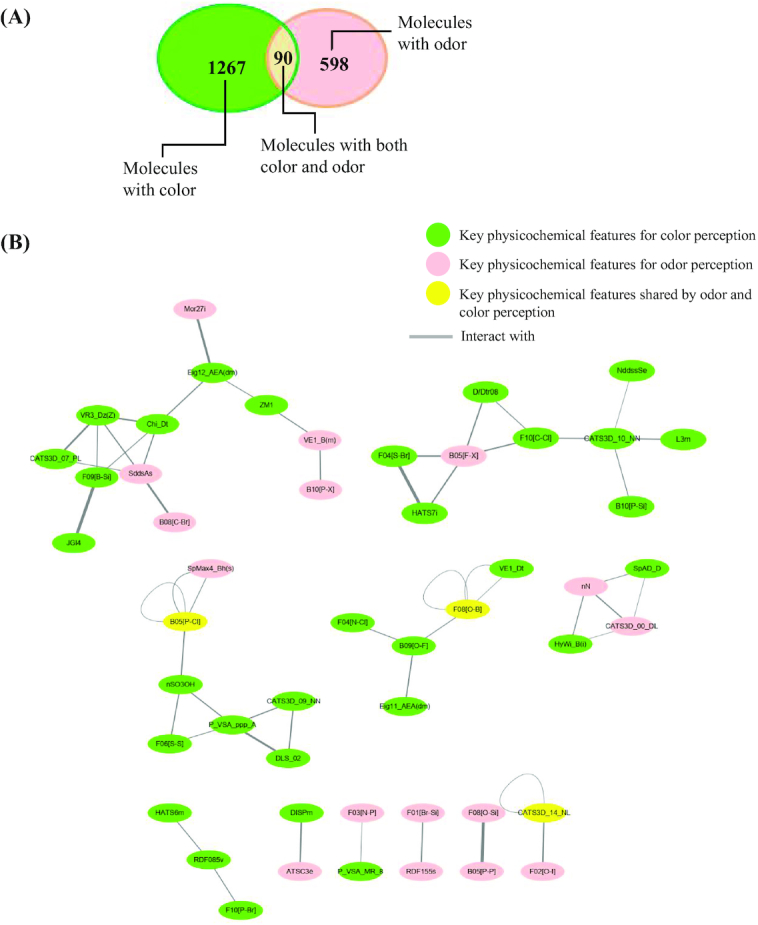
The correlations between color and olfaction perception. A. Of the 1,267 molecules with color, 90 also had odor information. B. Schematic diagram of the key physicochemical features for color and odor perceptions in the interactome. The key features for color perception were closely connected with the key features for odor perception. The distance of each line represents its correlation value.

To understand the correlation between color and odor, we collected 90 molecules with both color and odor information and analyzed the 2 groups using a *χ*^2^ test. The colors were divided into 2 categories (white/ colorless, other), as were the odors (odorless, other). A correlation was predicted for both types of perception for these molecules (*χ*^2^ = 17.445; *P* < 0.001). In the complex network of color and odor, key physicochemical features for color and odor prediction were converted into *z*-scores, and the relationship between each pair of attributes was evaluated by the Pearson correlation coefficient. More than 50 molecular descriptors were found to be interlinked prominently according to their correlation values (the absolute value of the Pearson correlation coefficients ≥ 0.300552) (Fig. 4). Three key features “B05[P-CI],” “F08[O-B],” and “CATS3D_14_NL” were shared for both color perception and odor perception.

## Discussion

Clarifying the underlying mechanism of color vision is inherently challenging because the cognitive process of color vision is multidimensional and includes crossover among the morphology and function of the human visual system [28–30]. Here, we established a framework for distinguishing color without wavelengths based on only 24 physicochemical features. We found that the accuracy and κ coefficient achieved using random forest (100%, 1) were better than those achieved with the DBN (95.23% ± 0.40%, 0.9400 ± 0.0030) in color prediction with 12 categories. For odor prediction with 12 categories, the accuracy and κ coefficient achieved using the DBN (94.75% ± 0.44%, 0.9397 ± 0.0031) were better than those achieved with the random forest (93.40% ± 0.31%, 0.9232 ± 0.0037). Above all, we believe that the machine learning method can be extended to predict both physicochemical properties.

Our findings also suggested that key physicochemical features in distinguishing color and odor are significantly correlated. The 2D Atom Pairs descriptors and many other descriptors interlink at the network between color and odor perception, indicating that both color and odor perceptions are partially determined by the physicochemical properties of the molecules and that color and odor perceptions are closely interrelated. With the prominently interlinked key physicochemical features identified in predicting color and odor, our results tend to call for more evidence for proving the practical relevance between these physicochemical features.

Previous studies on predicting odor have been conducted by the DREAM Olfaction Prediction Challenge [23, 31], with the best Pearson correlation coefficient achieved at ∼0.3 between observed and predicted perceptions. A dataset of 476 molecules sensed by 49 volunteers was applied, and the perceived attributes including the intensity were found to rate differently among the individuals, which considerably complicated the prediction challenge [31]. The winning algorithm of the DREAM challenge indicated that the random forest outperforms other base learners (linear, ridge, and support vector machine) in predicting odor [31]. Our study collected a total of 598 structurally diverse molecules and classified them into 12 diverse odors based on PubChem to avoid a subjective effect on odor perception. We added the DBN method and achieved the best result in odor prediction with a classification accuracy of 94.75% ± 0.44% for 12 categories using all features. In contrast, the performance of DBN using the key physicochemical features was less than satisfactory for the following reasons. First, the key physicochemical features identified in predicting color and odor were selected by random forest and genetic algorithm. These features may not be suitable for DBN, which can map the raw data to low-dimensional space by unsupervised learning in the pre-training phase. Second, although DBN is a multilayered recurrent neural network trained with energy-minimizing methods, the network structure of DBN has a great influence on the learning performance. The network structure of DBN utilizing all features and the key physicochemical features are exactly the same in our study. If other algorithm such as the Particle Swarm Optimization Algorithm could be used to optimize the number of DBN hidden-layer nodes, the performance of DBN network may improve.

In addition, odor sensing was found to be less accurate than that of color. Several factors may affect the accuracy of the AI in odor perception. First, odor perception is more subjective as a result of perceived biases, and it is challenging to confirm the number and character of its perceptual dimensions [32]. Defining a specific odor is especially difficult for human beings compared with other sensory modalities [33]. Second, the olfactory system involves high-dimensional input with attached arbitrary associations, whereas color vision occurs under predefined spatial conditions [12]. Thus, the processing demands of the 2 systems are not entirely consistent with each other. Third, the 2 systems use different strategies in temporal coding to convey information. The olfactory system uses temporal coding to increase its representational capacity, while the visual system uses temporal coding to reduce the redundancy [12].

In this study, we add new insight into the decoding of color vision, but the controlling and tuning of these codes require further investigation. Inspired by the key physicochemical features involved in color prediction, researchers may be able to develop materials with vivid colors for potential applications in sensing technologies, security, light-emitting sources, and paints [34–36].

### Potential implications

The ability to explain visual neural activities from the perspective of AI would also enable us to build an artificial vision system that could favorably stimulate the color vision of an individual. Once the perception process of human color vision is completely decoded, the AI platform may help in the design of artificial brain stimulation interfaces that can restore color vision and enable blind patients to “see” colors without biological eyes.

## Methods

### Random forest algorithm

Random forest is an ensemble learning method for regression and classification [37]. In a random forest model, each decision tree is built from a random sampling of samples and features, which can deliver generalized knowledge [37]. Furthermore, a random set of features is used to determine the best split at each node during the construction of a tree. Here, the dimensionality of the physicochemical data was high, with 5,270 descriptors per molecule, and the perception data matrix was sparse. By averaging hundreds of trees in this work, the effects of outliers and noise were reduced. The random forest parameter mTry (i.e., the number of input variables randomly chosen at each split) was set to 72 (square root of 5,270 features), while the other random forest parameter nTree (i.e., the number of trees to grow for each forest) was set to 100. *k*-fold cross-validation (*k* = 4) was applied for the classification.

### Deep belief network

DBN is a type of probability generative model that consists of multiple RBMs. The superposition of multiple RBMs solves the training problem of multiple layered neural networks. The overall training process of the DBN includes 2 stages: a pretraining stage and a fine-tuning stage [38]. (i) Pretraining stage: each RBM includes a visual layer and a hidden layer. There are no interlayer connections between the visual layer and hidden layer. After training the first RBM, the activation value of the hidden layer of the first RBM is input into the visual layer of the second RBM. (ii) Fine-tuning stage: with the help of the backpropagation neural network that resides after the last RBM and the chain rule of derivation, the DBN will be trained as a whole neural network. In this study, the input of the DBN is the vector consisting of 5,270 molecular descriptors. During the first stage of the DBN, the dimensions of the vector are compressed. During the second stage, the compressed vector can be used for classification.

We compared 3 DBN structures for the prediction of either color or odor, and optimizations of the parameters of each structure were conducted. The architecture that performed best in both color and odor prediction was the input layer with 5,270 neurons and only 1 RBM with 5,270 visible neurons and 500 hidden neurons. Moderate performance was achieved with the input layer with 5,270 neurons and 2 RBMs. One RBM was composed of 5,270 visible neurons and 2,000 hidden neurons, and the other contained 2,000 visible neurons and 500 hidden neurons. The worst performance was achieved with the input layer with 5,270 neurons and 3 RBMs. One RBM contained 5,270 visible neurons and 2,000 hidden neurons, 1 was composed of 2,000 visible neurons and 1,000 hidden neurons, and the last contained 1,000 visible neurons and 500 hidden neurons. Therefore, the best architecture was used in the follow-up prediction.

### Feature selection

Random forest algorithm and genetic algorithm [39, 40] were both applied to select the key features in this study. The random forest algorithm enable us to estimate the importance of each molecular descriptor by permuting the values of the descriptors across samples and computing the increases in prediction errors. The samples left-out in the training of each classifier (referred to as out-of-bag samples) are used for feature selection by determining the importance of different features during classification process. A value of “0” signifies that the feature corresponding to this bit is not needed for the classification; otherwise, the feature is needed for the classification. A total of 1,601 features were recognized as needed for the classification of color, and 1,820 were recognized as needed for the classification of odor in the random forest algorithm. To enable comparison with the selection results obtained by using both the genetic algorithm and random forest algorithm, similar numbers of key features were selected.

Genetic algorithms designed for feature selection can implement feature selection and classification processes simultaneously [41]. The accuracy of the random forest was adopted as the fitness evaluation function of the genetic algorithm. The chromosome coding method was binary coding, and the length of the chromosome was equal to the dimension of the feature vector. Because of the randomness of the genetic algorithm, the experiment was conducted 20 times. After running the genetic feature selection task 20 times, 24 descriptors were selected 18 times for color, and 39 descriptors were selected 16 times for odor.

### Feature ranking

Feature ranking for the random forest algorithm used out-of-bag permutation error. With the features selected from the genetic algorithm, feature ranking was performed to study which attributes were more important for classification. In this process, for a feature *A_i_* in the feature set {*A_1_, A_2__…_ A_n_*}, the validating accuracy for the original validation dataset is acc1. The validatiom accuracy obtained with the random permutation of *A_i_* is acc2. |acc2*−* acc1| is an indicator used to measure the importance of *A_i_*. Then, all features are compared with this indicator. Because of the randomness of the random forest, this process was conducted 20 times.

### Hierarchical clustering

Hierarchical approaches have the ability to simultaneously uncover multiple layers of a clustering structure [42]. The R heatmap package was used for clustering in this study.

### Statistical analysis

The data were collected using the Qualtrics Web-based questionnaire package and analyzed using IBM SPSS Statistics version 24 (SPSS, RRID:SCR_002865).

## Availability of Supporting Source Code and Requirements

Project name: Color Odor Prediction

Project home page: https://github.com/Hugo0512/ColorOdorprediction

Operating system: Platform independent

Programming language: MATLAB

License: MIT

## Availability of Supporting Data and Materials

All methods were implemented with MATLAB R2016a (MATLAB, RRID:SCR_001622) on an HP Z420 workstation with Intel Xeon CPU E5-1620 v2 at 3.70 GHz and 16 GB RAM. The operating system was Windows 7. Data corresponding to the molecules used in this study are presented in Supplementary Data 1–3, and archives of all the code and all supporting data are available in the *GigaScience* GigaDB repository [43].

## Additional Files

**Table S1:** Attribute importance ranking of color.

**Table S2:** Attribute importance ranking of odor.

**Table S3:** The results for each fold in the 4-fold cross-validation.

**Figure S1:** The prediction accuracies of random forest models for 12 colors and 12 odors using all features.

**Supplementary Data 1:** The datasets of the 1,267 structurally diverse molecules labeled with 12 diverse colors and 5,270 molecular descriptors.

**Supplementary Data 2:** The datasets of the 598 structurally diverse molecules labeled with 12 diverse odors and 5,270 molecular descriptors.

**Supplementary Data 3:** The datasets of the 90 molecules with both color and odor information.

giaa011_GIGA-D-19-00112_Original_Submission

giaa011_GIGA-D-19-00112_Revision_1

giaa011_GIGA-D-19-00112_Revision_2

giaa011_GIGA-D-19-00112_Revision_3

giaa011_Response_to_Reviewer_Comments_Original_Submission

giaa011_Response_to_Reviewer_Comments_Revision_1

giaa011_Response_to_Reviewer_Comments_Revision_2

giaa011_Reviewer_1_Report_Original_Submission Hongyang Li -- 6/22/2019 Reviewed

giaa011_Reviewer_1_Report_Revision_1 Hongyang Li -- 11/6/2019 Reviewed

giaa011_Reviewer_2_Report_Original_Submission Neetika Nath, Ph.D. -- 7/30/2019 Reviewed

giaa011_Reviewer_2_Report_Revision_1 Neetika Nath, Ph.D. -- 11/28/2019 Reviewed

giaa011_Reviewer_2_Report_Revision_2 Neetika Nath, Ph.D. -- 1/6/2020 Reviewed

giaa011_Supplemental_Files

## Abbreviations

AI: artificial intelligence; CPU: central processing unit; DBN: deep belief network; Dragon: software for the calculation of molecular descriptors; NCBI: National Center for Biotechnology Information; RAM: random access memory; RBM: restricted Boltzmann machine.

## Completing Interests

The authors declare that they have no competing interests.

## Funding

This study was funded by the National Key R&D Program of China (2018YFC0116500), the Key Research and Development Program of Guangdong Province (No. 2018B010109008), and the National Natural Science Foundation of China (81770967, 81822010). The funders had no role in the study design, data collection, and analysis, the decision to publish, or the preparation of the manuscript.

## Authors' Contributions

H.T.L., X.Y.Z., and D.R.L. conceived and designed the prediction algorithm. X.Y.Z., K.Z., D.R.L., and L.H. were responsible for data management and performing the computational analyses. R.X.W., Z.Z.L., X.H.W., and E.P.L. analyzed the discriminative features and prepared the figures. H.T.L., X.Y.Z., and D.R.L. contributed to the writing of the manuscript. Z.Y., C.C., X.S.G., K.X.C., K.H., X.Y.L., and Z.Q.H. contributed to the critical review of the study, and all authors read and approved the final manuscript.
