# Supplementary material for: Artificial intelligence deciphers codes for color and odor perceptions based on large-scale chemoinformatic data
Source: Gigascience. 2020 Feb 26;9(2):giaa011. doi: 10.1093/gigascience/giaa011 (PMC7043059; doi:10.1093/gigascience/giaa011)
Supplement: giaa011_GIGA-D-19-00112_Original_Submission [file giaa011_giga-d-19-00112_original_submission.pdf]

GigaScience

Artificial intelligence deciphers codes for color and odor perceptions based on large-scale chemoinformatic data

--Manuscript Draft--

**Manuscript Number:**	GIGA-D-19-00112
**Full Title:**	Artificial intelligence deciphers codes for color and odor perceptions based on large-scale chemoinformatic data
**Article Type:**	Research
**Funding Information:**	National Key Research and Development Program of China (2018YFC0116500) — Prof. Haotian Lin<br>National Natural Science Foundation of China (91546101) — Prof. Haotian Lin<br>National Natural Science Foundation of China (81822010) — Prof. Haotian Lin

Abstract:

Color perception by an organism is based on the different reflectance spectra of materials within a visible wavelength range 1. Although a variety of physical and chemical mechanisms, including ligand field effects, molecular orbital effects, and energy band effects, have been proposed to demystify the origin of color 2, 3, the ability to recognize colors at optical scales (i.e., from nanometers to microns) is still lacking. The physicochemical basis of color coding has not been explored completely, and how color perception is integrated with other sensory input, typically odor, is unclear. Here, we developed an artificial intelligence platform to train algorithms for distinguishing color and odor based on large-scale physicochemical features of 1267 and 598 structurally diverse molecules, respectively. The predictive accuracies using the random forest and deep belief network for the prediction of color were 100.0% ± 0.0% and 100.0% ± 0.0% (mean ± SD), respectively. Twenty-three physicochemical features were sufficient for the accurate prediction of color. A positive correlation between the color coding and odor coding properties of the molecules was predicted. Twenty-nine descriptors were found to interlink prominently in color and odor perceptions. These findings extend our understanding of the molecular and structural basis of color vision and reveal the interrelationship between color and odor perceptions in nature.

**Corresponding Author:**	Haotian Lin, Ph.D., M.D.<br>Sun Yat-Sen University Zhongshan Ophthalmic Center<br>CHINA
**Corresponding Author Secondary Information:**	
**Corresponding Author's Institution:**	Sun Yat-Sen University Zhongshan Ophthalmic Center
**Corresponding Author's Secondary Institution:**	
**First Author:**	Xiayin Zhang
**First Author Secondary Information:**	
**Order of Authors:**	Xiayin Zhang
	Kai Zhang
	Duoru Lin
	Yi Zhu
	Chuan Chen
	Ruixin Wang

Powered by Editorial Manager® and ProduXion Manager® from Aries Systems Corporation

	Zhenzhen Liu
	Xiaohang Wu
	Erping Long
	Weiyi Lai
	Wenben Chen
	Zhiqiang He
	Haotian Lin, Ph.D., M.D.
**Order of Authors Secondary Information:**	
**Additional Information:**	
**Question**	**Response**
Are you submitting this manuscript to a special series or article collection?	No
**Experimental design and statistics**<br><br>Full details of the experimental design and statistical methods used should be given in the Methods section, as detailed in our Minimum Standards Reporting Checklist. Information essential to interpreting the data presented should be made available in the figure legends.<br><br>Have you included all the information requested in your manuscript?	Yes
**Resources**<br><br>A description of all resources used, including antibodies, cell lines, animals and software tools, with enough information to allow them to be uniquely identified, should be included in the Methods section. Authors are strongly encouraged to cite Research Resource Identifiers (RRIDs) for antibodies, model organisms and tools, where possible.<br><br>Have you included the information requested as detailed in our Minimum Standards Reporting Checklist?	Yes
**Availability of data and materials**	Yes

All datasets and code on which the conclusions of the paper rely must be either included in your submission or deposited in publicly available repositories (where available and ethically appropriate), referencing such data using a unique identifier in the references and in the "Availability of Data and Materials" section of your manuscript.<br><br>Have you have met the above requirement as detailed in our Minimum Standards Reporting Checklist?	

Powered by Editorial Manager® and ProduXion Manager® from Aries Systems Corporation

Manuscript Click here to download Manuscript Manuscript 0408.docx

Artificial intelligence deciphers codes for color and odor perceptions based on large-scale chemoinformatic data

Xiayin Zhang[1†] (zhangxiayin@gzzoc.com), Kai Zhang[1,2†] (hugo88315@163.com), Duoru Lin[1†] (linduoru@sina.com), Yi Zhu[1,3] (y.zhu17@med.miami.edu), Chuan Chen[1,3] (c.chen30@med.miami.edu), Ruixin Wang[1] (ruiruiw413@aliyun.com), Zhenzhen Liu[1] (liu_zhenzhen@qq.com), Xiaohang Wu[1] (1034281949@qq.com), Erping Long[1] (longerping@qq.com), Weiyi Lai[1] (zsylaiwy@163.com), Wenben Chen[1] (weberchan@foxmail.com), Zhiqiang He[4] (hezq@bupt.edu.cn) and Haotian Lin[1*] (haot.lin@hotmail.com).

[1]State Key Laboratory of Ophthalmology, Zhongshan Ophthalmic Center, Sun Yat-sen University, Guangzhou 510060, China;

[2]School of Computer Science and Technology, Xidian University, Xi'an 710000, China;

[3]Department of Molecular and Cellular Pharmacology, University of Miami Miller School of Medicine, Miami, Florida 33136, USA;

[4] Key Laboratory of Universal Wireless Communications, Beijing University of Posts and Telecommunications, Beijing 100876, China.

[†] These authors contributed equally to this work.

* **Corresponding Author:**

Prof. Haotian Lin

Xian Lie South Road 54#, Guangzhou, China, 510060

Telephone: +86-13802793086, Email address: haot.lin@hotmail.com

Abstract

Background

Color vision is the ability to detect, distinguish, and analyze the wavelength distributions of light independent of the total intensity. It mediates the interaction between an organism and its environment in multiple important aspects, including mate choice, camouflage, and speciation. However, the physicochemical basis of color coding has not been explored completely, and how color perception is integrated with other sensory input, typically odor, is unclear.

Results

Here, we developed an artificial intelligence platform to train algorithms for distinguishing color and odor based on large-scale physicochemical features of 1267 and 598 structurally diverse molecules, respectively. The predictive accuracies using the random forest and deep belief network for the prediction of color were 100.0% ± 0.0% and 100.0% ± 0.0% (mean ± SD), respectively. Twenty-three physicochemical features were sufficient for the accurate prediction of color. A positive correlation between the color coding and odor coding properties of the molecules was predicted. Twenty-nine descriptors were found to interlink prominently in color and odor perceptions.

Conclusions

Our random forest model and DBN accurately predicted the colors and odors of structurally diverse molecules. These findings extend our understanding of the molecular and structural basis of color vision and reveal the interrelationship between

color and odor perceptions in nature.

Keywords: color perception; structure-color relationships; random forest; deep belief network; physicochemical features.

Background

Color perception by an organism is based on the different reflectance spectra of materials within a visible wavelength range [1]. Although a variety of physical and chemical mechanisms, including ligand field effects, molecular orbital effects, and energy band effects, have been proposed to demystify the origin of color [2, 3], the ability to recognize colors at optical scales (i.e., from nanometers to microns) is still lacking.

Nature creates various colorful materials based on physicochemical properties including the topological and geometrical properties that we humans cannot easily see [4, 5]. For instance, the color changes from bright yellow through reddish–purple up to blue when the size of gold samples is decreased [6]. The different colors of disubstituted benzenes were discovered to be related to differences in the molecular structure with ortho, meta and para substitutions [7, 8]. The odors of chemicals are also fully encoded within their specific physicochemical properties [9, 10]. The compositions and structures of the functional groups have been suggested to be crucial for the perception of aroma [11]. Moreover, evidence of the interaction between color vision and olfaction has been discovered [12]. For example, the odor of the host plant can modify the color sensed by a swallowtail butterfly [13]. The odor of a wine can be predicted according to its color [14].

Additionally, the perceived intensity of an odor is positively correlated with the intensity of color [15, 16]. Neuroimaging and repetitive transcranial magnetic stimulation studies showed that high-level odor processing also actives the visual cortex [17, 18]. However, the relationship between color and odor in terms of the molecular physicochemical properties is largely unknown.

Artificial intelligence (AI) tools can be optimized to infer the innate laws of natural processes following machine learning tasks based on large-scale data sets and make predictions of the unknown [19, 20]. In the chemical sciences, AI has been used to guide chemical and material design, synthesis, characterization, and modeling [21, 22]. Previous researchers have equipped AI with a “nose” to predict human olfactory perception from the physicochemical features of 476 molecules and 21 perceptual attributes perceived by 49 individuals [23].

Here, we developed a random forest model and deep belief network (DBN) to predict the colors of chemicals based on their molecular descriptors. We applied genetic algorithms for feature selection to identify the descriptors that contribute most to the predictive accuracies. In addition, we investigated the connection between the key physicochemical features in color and odor coding to unravel the commonality between visual and olfactory perception.

Data Description

Data collection and labeling. A total of 1267 structurally diverse molecules were used in this study, both the color and three-dimensional (3D) structure data of these

molecules were collected from the key chemical information resource at the U.S. National Center for Biotechnology Information, PubChem [24] (https://pubchem.ncbi.nlm.nih.gov) between June 1, 2017, and November 30, 2017. Molecules with colors or odors that are difficult to define were excluded. The data set was labeled three times with 12 diverse colors, including yellow (257 molecules), white (301 molecules), orange (31 molecules), red (16 molecules), purple (11 molecules), green (24 molecules), blue (9 molecules), brown (20 molecules), amber (15 molecules), gray (6 molecules), black (17 molecules) and colorless (560 molecules). The data set of odors was classified into 12 diverse odors, including ammonia (37 molecules), aromatic (36 molecules), characteristic (27 molecules), flower (19 molecules), fruity (29 molecules), mild (38 molecules), other (127 molecules), pleasant (16 molecules), unpleasant (23 molecules), spicy (54 molecules), sweet (30 molecules) and odorless (162 molecules).

Physicochemical features of the molecules. The PubChem compound identifier (CID) for each molecule was provided and used for PubChem or other database searches (Supplementary data). We applied a commercial chemoinformatics software package Dragon (version 7.0, https://chm.kode-solutions.net/products_dragon_papers.php) to generate 5270 physicochemical descriptors for each of the molecules, including the simplest atom types, functional groups and fragment counts, topological and geometrical descriptors, 3D descriptors, several property estimations (such as log*P*) and drug-like and lead-like alerts (such as the Lipinski's alert). These molecular descriptors are formal mathematical representations of a molecule and include their

definition, symbols and labels, formulas, some numerical examples, data and molecular graphs, as presented in the Handbook of Molecular Descriptors [25]. We have replaced all of the "NaN" entries with "0" in the dataset preprocessing. The overall workflow is shown in Figure 1.

Results

Color prediction

After applying the synthetic minority oversampling technique (SMOTE) to minimize the potential for misclassification caused by imbalances in the data structure, random forest and DBN were applied for the *in silico* test. Using *k*-fold cross-validations (k = 4), the random forest model identified and utilized the most discriminative features with 100.00% ± 0.0% (mean ± SD) accuracy in the prediction of twelve colors (Figure 2A). As a type of probability generation model consisting of multiple restricted Boltzmann machines (RBMs), the DBN also performed excellently, with a predictive accuracy of 100.00% ± 0.0% (mean ± SD) (Figure 2B).

Key physicochemical features for color perception

The random forest enables us to estimate the importance of each molecular descriptor by permuting the values of the descriptors across samples and computing the increases in prediction errors. After running the genetic feature selection task 20 times, twenty-three descriptors were selected as the key physicochemical features with a classification accuracy of 99.90% by using *k*-fold cross-validations (k = 4). The molecular descriptor "RTe+" ranked first, followed by "GATS6v", "B09[P-I]",

"SM2_B(s)" and "F07[O-X]". The heat map of the hierarchical cluster analysis between the twenty-three key features and the twelve colors is shown in Figure 2C. SM2_B(s), SpMax6_Bh(m), SpMAD_B(e), ATS2 m, nArOX and F09[Cl-Br] were the main contributors to white, whereas GATS6v was the most important factor in predicting red. B09[P-I] and F04[S-X] were the most important descriptors in predicting colorless. Information relevant to the key physicochemical features for color perception is reported in Supplementary Table 1.

Distinction and connection with olfaction perception

We next applied the AI platform to predict odor perception based on physicochemical features. A total of 598 structurally diverse molecules were collected and classified into twelve diverse odors based on PubChem [24], including pleasant, unpleasant, ammonia, aromatic, flowery, fruity, spicy, sweet, mild, odorless, characteristic, and other. The accuracies of the odor prediction were 89.59% ± 0.46% for the random forest model using k-fold cross-validations (k = 4) and 89.31 ± 0.01% for the DBN (Figure 3A, B). After running the genetic feature selection task 20 times, thirty-two descriptors were selected as the key physicochemical features, with a classification accuracy of 89.82% ± 0.76% in k-fold cross-validations (k = 4). The heat map of the hierarchical cluster analysis between the thirty-two key features and the twelve odors is shown in Figure 3C. Information relevant to the key physicochemical features for the odor perception is presented in Supplementary Table 2.

To understand the correlation between color and odor, we collected 90 molecules with

both color and odor information and analyzed the two groups using a chi-square test. The colors were divided into two categories (white, colorless/other), as well as the odors (odorless/other). A correlation was predicted for both types of perception for these molecules ($\chi^2 = 17.445$; $P < 0.001$). In the complex network of predicting color and odor, twenty-nine molecular descriptors were found to interlink prominently according to the correlation values (The absolute value of the Pearson correlation coefficients > 0.3). The “RTe+” descriptor that ranked first in key features for color perception was closely connected with the key features for odor perception, including “B10[S-Br]”, “CATS2D_01_NL”, “SpPos_Dz(i)”, “Mor19p”, “F10[O-B]”, “CATS3D_08_PN” and “SM3_Dz(Z)”. The key descriptor “Mor19p” for odor perception tended to interact with the key features for color perception including “RTe+”, “SM2_B(s)”, “SM3_B(p)”, “B01[B-Si]” and “B02[C-F]” (Figure 4).

Discussion

Clarifying the underlying mechanism of color vision is inherently challenging, as the cognitive process of color vision is multidimensional and includes crossover among the morphology and function of the human visual system [26-28]. Here, we established a terse framework for distinguishing color without wavelength based on only 23 physicochemical features. Our findings suggest that several key physicochemical features for distinguishing color and odor are connected. One of the most influential features is the GETAWAY (Geometry, topology and atom-weights assembly) descriptor “RTe+”, which refers to the *R* maximal index weighted by atomic Sanderson electronegativities and can be obtained from the leverage/geometry matrix

[6, 29]. The 2D autocorrelation descriptors and many other descriptors interlink at the network between color and odor perceptions, indicating that both color and odor perceptions are partially determined by the physicochemical properties of the molecules and color and odor perceptions are closely interrelated.

The decision-making of the random forest model was comparable to the DBN regarding the prediction tasks. However, odor sense was found to be less accurate than those of color. Several factors may affect the accuracy of the AI in odor perception. First, odor perception is more subjective based on perceived biases, and it is challenging to confirm the number and character of its perceptual dimensions [30]. Defining a specific odor is especially difficult for human beings compared with other sensory modalities [31]. Second, the olfactory system involves high-dimensional input with attached arbitrary associations, whereas color vision occurs under predefined spatial conditions [12]. Thus, the processing demands of the two systems are not entirely consistent with each other. Third, the two systems employ different strategies in temporal coding to convey information. The olfactory system uses temporal coding to increase its representational capacity, while the visual system uses temporal coding to reduce the redundancy [12].

Potential implications

In this study, we add new insight in the decoding of color vision, but the controlling and tuning of these codes require further investigation. Inspired by the key physicochemical features involved in color prediction, researchers may be able to

develop materials with vivid colors for potential application in sensing technologies, security, light-emitting sources, and paints [32-34]. The ability to explain visual neural activities from the perspective of AI would also enable us to build an artificial vision system that could favorably stimulate the color vision of an individual [26]. Once the perception process of human color vision is completely decoded, the AI platform may help in the design of artificial brain stimulation interfaces that can restore color vision and enable blind patients to "see" colors without biological eyes.

Methods

Synthetic minority oversampling technique (SMOTE)

The classifiers, which included decision trees and neural networks, were biased in favor of the majority class when learning from an imbalanced dataset [35]. SMOTE is an oversampling technique for avoiding this bias of the classification results [36]. Each instance is considered to be a vector, and synthetic samples are generated along the line between the minority sample and its nearest neighbor in SMOTE. After the pretreatment, the data set with 12 diverse colors was presented as yellow (257 data), white (301 data), orange (310 data), red (320 data), purple (330 data), green (312 data), blue (270 data), brown (300 data), amber (300 data), gray (300 data), black (306 data) and colorless (560 data). The data set with 12 diverse odors was presented as ammonia (148 data), aromatic (144 data), characteristic (135 data), flower (133 data), fruity (145 data), mild (152 data), other (127 data), pleasant (160 data), unpleasant (161 data), spicy (162 data), sweet (150 data) and odorless (162 data).

Random forest algorithm

Random forest is an ensemble learning method for regression and classification [37]. In a random forest model, each decision tree is built from a random sampling of samples and features, which can effectively avoid overfitting and deliver generalized knowledge. Furthermore, a random set of features is used to determine the best split at each node during the construction of a tree. Here, the dimensionality of the physicochemical data was high, with 5270 descriptors per molecule, and the perception data matrix was sparse. By averaging hundreds of trees in this work, overfitting was avoided, and the effects of outliers and noise were reduced. The random forest parameter mTry (i.e., the number of input variables randomly chosen at each split) was set to 72 (square root of 5270 features), while the other random forest parameter nTree (i.e., the number of trees to grow for each forest) was set to 1000. k-fold cross-validation (k = 4) was applied for the classification.

Deep belief network (DBN)

DBN is a type of probability generative model that consists of multiple RBMs. The superposition of multiple RBMs solves the training problem of a multiple layered neural network. The overall training process of the DBN includes two stages: a pretraining stage and a fine-tuning stage [38]. 1) Pretraining stage: Each RBM includes a visual layer and a hidden layer. There are no interlayer connections between the visual layer and hidden layer. After training the first RBM, the activation value of the hidden layer of the first RBM is input into the visual layer of the second RBM. 2) Fine-tuning stage: With the help of the BP neural network that resides after the last RBM and the

chain rule of derivation, the DBN will be trained as a whole neural network. In this study, the input of DBN is the vector consists of 5270 molecular descriptors. During the first stage of DBN, the dimension of the vector is compressed. During the second stage, the compressed vector can be used to classification.

Genetic algorithms for feature selection

Because the high dimensionality of the feature vector of the molecules leads to difficulties in distinguishing which feature is helpful for classification, the genetic algorithm and random forest algorithm are combined so that the important features can be selected in this study. Genetic algorithms for feature selection can implement feature selection and classification processes simultaneously. The accuracy of the random forest here was adopted as the fitness evaluation function of the genetic algorithm. The chromosome coding method is binary coding, and the length of the chromosome equals the dimension of the feature vector. A bit 0 signifies that the feature corresponding to this bit is not needed in the classification; otherwise, the feature is needed in the classification. Because of the randomness of the genetic algorithm, the experiment was conducted 20 times. All of the attributes chosen by genetic feature selection in color and odor prediction were converted into z-scores, and the relationships between each pair of attributes were evaluated by the Pearson correlation coefficient. The cutoff for the weights is chosen between -0.3 to 0.3.

With the selected features from the genetic algorithm, feature ranking was performed to study which attributes were more important for classification. For a feature A_i in the feature set $\{A_1, A_2 \dots A_n\}$, the validating accuracy for the original validating dataset is

acc1. The validating accuracy obtained with the random permutation of A_i is *acc2*. $|acc2-acc1|$ is an indicator used to measure the importance of A_i. Then, all features are compared with this indicator. Because of the randomness of the random forest, this process was conducted 20 times. For the color and odor classifications, the numbers of selected features are twenty-three and thirty-two, respectively.

Hierarchical clustering

Hierarchical approaches have the ability to simultaneously uncover multiple layers of a clustering structure [39]. The R heatmap package was used for clustering in this study.

Statistical analysis

The data were collected using the Qualtrics Web-based questionnaire package and analyzed using IBM SPSS Statistics version 24.

Availability of supporting data

Data of the molecules used in this study are presented in Supplementary Data1-3. The source code of this study is presented in https://github.com/Hugo0512/ColorOdorprediction.

Additional files

Table S1. Attribute importance ranking of color.

Table S2. Attribute importance ranking of odor.

Supplementary Data1. The datasets of the 1267 structurally diverse molecules labeled with 12 diverse colors and 5270 molecular descriptors.

Supplementary Data2. The datasets of the 598 structurally diverse molecules labeled with 12 diverse odors and 5270 molecular descriptors.

Supplementary Data3. The datasets of the 90 molecules with both color and odor information.

Declarations

List of abbreviations

3D, Three dimensional; AI, Artificial intelligence; CID, PubChem compound identifier; DBN, Deep belief network; DRAGON, Software for the calculation of molecular descriptors; GETAWAY, Geometry, topology and atom-weights assembly; RBM, Restricted Boltzmann machine; SMOTE, Synthetic minority oversampling technique.

Completing interests

The authors declare that they have no competing interests.

Funding

This study was funded by the National Key R&D Program of China (2018YFC0116500), the National Natural Science Foundation of China (91546101, 81822010). The funders had no role in the study design, data collection and analysis, the decision to publish or the preparation of the manuscript.

Author contributions

H.T.L., X.Y.Z. and D.R.L. conceived and designed the prediction algorithm, K.Z., X.Y.Z. and D.R.L. were responsible for data management and the performing of the computational analyses. R.X.W., Z.Z.L., and X.H.W. analyzed the discriminative features and prepared the figures. H.T.L., X.Y.Z. and D.R.L. contributed to the writing of the manuscript. Z.Y., C.C., E.P.L., W.Y.L., W.B.C., and Z.Q.H. contributed to the

critical review of the study, and all authors read and approved the final manuscript.

Acknowledgments

We thank Xiaoming Chen (School Of Chemistry, Sun Yat-sen University) for reading, discussing and providing constructive comments for the manuscript.

Author Information

Correspondence and requests for materials should be addressed to H.T.L. (haot.lin@hotmail.com). Readers are welcome to comment on the online version of the paper.

References

1. Chang, L., Bao, P. & Tsao, D.Y. The representation of colored objects in macaque color patches. *Nat Commun* 2017; 8, 2064.
2. Kinoshita, S., Yoshioka, S. & Miyazaki, J. Physics of structural colors. *Rep Prog Phys* 2008; 71, 175-180.
3. Nassau, K. 7–The Physics and Chemistry of Color : the 15 Mechanisms. *Science of Color* 2003; 18, 247-280.
4. Wilkinson, F.A. & Murillo, S.G. Advanced inorganic chemistry. 1988. Wiley.
5. McMurry, John. Organic chemistry. 2007. Brooks Cole.
6. Hallenbeck. Recent Advances in QSAR Studies. *Challenges & Advances in Computational Chemistry & Physics* 2010; 8, 31-32.
7. Paul, A. The use of nanocrystals in biological detection. *Nat Biotechnol* 2004; 22, 47-52.
8. Chen, F. & Gerion, D. Fluorescent CdSe/ZnS Nanocrystal−Peptide Conjugates

for Long-term, Nontoxic Imaging and Nuclear Targeting in Living Cells. *Office of Scientific & Technical Information Technical Reports* 2004; 4, 1827-1832.

9. Rossiter, K.J. Structure−Odor Relationships. *Chemical ReviewsChemical ReviewsChem. Rev.* 1996; 96, 3201-3240.

10. Turin, L. A method for the calculation of odor character from molecular structure. *J Theor Biol* 2002; 216, 367-385.

11. Czerny, M., Brueckner, R., Kirchhoff, E., Schmitt, R. & Buettner, A. The influence of molecular structure on odor qualities and odor detection thresholds of volatile alkylated phenols. *Chem Senses* 2011; 36, 539.

12. Gire, D.H., *et al.* Temporal processing in the olfactory system: can we see a smell. *Neuron* 2013; 78, 416-432.

13. Yoshida, M., Itoh, Y., Ômura, H., Arikawa, K. & Kinoshita, M. Plant scents modify innate color preference in foraging swallowtail butterflies. *Biol Lett* 2015; 11.

14. Morrot, G., Brochet, F. & Dubourdieu, D. The Color of Odors. *Brain & Language* 2001; 79, 309-320.

15. Zellner, D.A. & Kautz, M.A. Color affects perceived odor intensity. *J Exp Psychol Hum Percept Perform* 1990; 16, 391-397.

16. Dubose, C.N., Cardello, A.V. & Maller, O. Effects of colorants and flavorants on identification, perceived flavor and hedonic quality of fruit-flavored beverages and cake. *J Food Sci* 2010; 45, 1393-1399.

17. Royet, J.P., *et al.* Functional anatomy of perceptual and semantic processing for odors. *J Cogn Neurosci* 1999; 11, 94-109.

18. Jadauji, J.B., *et al.* Modulation of olfactory perception by visual cortex stimulation. *Journal of Neuroscience the Official Journal of the Society for Neuroscience* 2012; 32, 3095.

19. Gershman, S.J., Horvitz, E.J. & Tenenbaum, J.B. Computational rationality: A converging paradigm for intelligence in brains, minds, and machines. *Science* 2015; 349, 273-278.

20. Sanchez-Lengeling, B. & Aspuru-Guzik, A. Inverse molecular design using machine learning: Generative models for matter engineering. *Science* 2018; 361, 360-365.

21. Butler, K.T., Davies, D.W., Cartwright, H., Isayev, O. & Walsh, A. Machine learning for molecular and materials science. *Nature* 2018; 559, 547-555.

22. Paruzzo, F.M., *et al.* Chemical shifts in molecular solids by machine learning. *Nat Commun* 2018; 9, 4501.

23. Keller, A., *et al.* Predicting human olfactory perception from chemical features of odor molecules. *Science* 2017; 355, 820-826.

24. Kim, S., *et al.* PubChem Substance and Compound databases. *Nucleic Acids Res* 2016; 44, D1202-1213.

25. Todeschini, R. & Consonni, V. Handbook of Molecular Descriptors. 2000.

26. Solomon, S.G. & Lennie, P. The machinery of colour vision. *Nat Rev Neurosci* 2007; 8, 276-286.

27. Bennett, A. .D. & Théry, M. Avian Color Vision and Coloration: Multidisciplinary Evolutionary Biology. *Am Nat* 2007; 169, S1-1S6.

28. Kelber, A. & Osorio, D. From spectral information to animal colour vision: experiments and concepts. *Proceedings: Biological Sciences* 2010; 277, 1617-1625.

29. Maldonado, A.G., Doucet, J.P., Petitjean, M. & Fan, B.T. Molecular similarity and diversity in chemoinformatics: from theory to applications. *Mol Divers* 2006; 10, 39-79.

30. Kaeppler, K. & Mueller, F. Odor classification: a review of factors influencing perception-based odor arrangements. *Chem Senses* 2013; 38, 189-209.

31. Wippich, W., Mecklenbräuker, S. & Trouet, J. Implicit and explicit memories of odors. *Archiv Für Psychologie* 1989; 141, 195.

32. Hwang, J., *et al.* Electro-tunable optical diode based on photonic bandgap liquid-crystal heterojunctions. *Nat Mater* 2005; 4, 383-387.

33. Lee, H.S., Shim, T.S., Hwang, H., Yang, S.M. & Kim, S.H. Colloidal Photonic Crystals toward Structural Color Palettes for Security Materials. *Chemistry of Materials* 2013; 25, 2684-2690.

34. Sung Yeun, C., *et al.* Mesoporous bragg stack color tunable sensors. *Nano Lett* 2006; 6, 2456-2461.

35. Chawla, N.V. C4. 5 and imbalanced data sets: investigating the effect of sampling method, probabilistic estimate, and decision tree structure. *Proceedings of the Icml'03 Workshop on Class Imbalances*, 2003.

36. Chawla, N.V., Bowyer, K.W., Hall, L.O. & Kegelmeyer, W.P. SMOTE: Synthetic Minority Over-sampling Technique. *J Artif Intell Res* 2002; 16, 321-357.

37. Breiman, L. Random Forests. *Mach Learn* 2001; 45, 5-32.

38. Le, R.N. & Bengio, Y. Representational power of restricted boltzmann machines and deep belief networks. *Neural Comput* 2008; 20, 1631-1649.

39. Eisen, M.B., *et al.* Cluster analysis and display of genome-wide expression patterns, 1998; 14863-14868.

Supplementary Materials

Table S1. Attribute importance ranking of color.

Ranking	Descriptor Name	Description	Block
1	RTe+	R maximal index weighted by the Sanderson electronegativity	GETAWAY descriptors
2	GATS6v	Geary autocorrelation of lag 6 weighted by the van der Waals volume	2D autocorrelations
3	B09[P-I]	Presence/absence of P - I at topological distance 9	2D Atom Pairs
4	SM2_B(s)	Spectral moment of order 2 from the Burden matrix weighted by the I-State	2D matrix-based descriptors
5	F07[O-X]	Frequency of O - X at topological distance 7	2D atom Pairs
6	nArOX	Number of hypohalogenides (aromatic)	Functional group counts
7	SM3_B(p)	Spectral moment of order 3 from the Burden matrix weighted by the polarizability	2D matrix-based descriptors
8	SpMax6_Bh(m)	Largest eigenvalue n. 6 of Burden matrix weighted by mass	Burden eigenvalues

9	B01[B-Si]	Presence/absence of B - Si at topological distance 1	2D atom Pairs
10	F09[Cl-Br]	Frequency of Cl - Br at topological distance 9	2D atom Pairs
11	AVS_D	Average vertex sum from topological distance matrix	2D matrix-based descriptors
12	B02[C-F]	Presence/absence of C - F at topological distance 2	2D Atom Pairs
13	ATS2 m	Broto-Moreau autocorrelation of lag 2 (log function) weighted by mass	2D autocorrelations
14	HATSu	Leverage-weighted total index / unweighted	GETAWAY descriptors
15	SM1_B(s)	spectral moment of order 1 from the Burden matrix weighted by I-State	2D matrix-based descriptors
16	SM1_B(v)	spectral moment of order 1 from Burden matrix weighted by the van der Waals volume	2D matrix-based descriptors
17	piPC06	Molecular multiple path count of order 6	Walk and path counts
18	F04[S-X]	Frequency of S - X at topological distance 4	2D atom Pairs
19	CATS2D_09_DD	CATS2D Donor-Donor at lag 09	CATS 2D
20	B07[S-S]	Presence/absence of S - S at topological distance 7	2D Atom Pairs
21	Satot	Total surface area from P_VSA-like descriptors	Molecular properties
22	SpMAD_B(e)	spectral mean absolute deviation from the	2D matrix-based descriptors

		Burden matrix weighted by the Sanderson electronegativity	
23	SM11_EA(dm)	Spectral moment of order 11 from the edge adjacency mat. weighted by the dipole moment	Edge adjacency indices

Table S2. Attribute importance ranking of odor.

Ranking	Descriptor Name	Description	Block
1	EE_Dt	Estrada-like index (log function) from detour matrix	2D matrix-based descriptors
2	TPSA(Tot)	Topological polar surface area using N,O,S,P polar contributions	Molecular properties
3	nThiranes	Number of thiranes	Functional group counts
4	Mor23i	Signal 23 / weighted by the ionization potential	3D-MoRSE descriptors
5	R6s	R autocorrelation of lag 6 / weighted by the I-state	GETAWAY descriptors
6	CATS3D_11_DN	CATS3D Donor-Negative BIN 11 (11.000 - 12.000 Å)	CATS 3D
7	SM13_EA(dm)	Spectral moment of order 13 from the edge adjacency matrix weighted by the dipole moment	Edge adjacency indices

8	SpMax_X	Leading eigenvalue from the chi matrix	2D matrix-based descriptors
9	CATS3D_02_AN	CATS3D Acceptor-Negative BIN 02 (2.000 - 3.000 Å)	CATS 3D
10	B10[S-Br]	Presence/absence of S - Br at topological distance 10	2D Atom Pairs
11	CATS2D_01_NL	CATS2D Negative-Lipophilic at lag 01	CATS 2D
12	SpPos_Dz(i)	Spectral positive sum from the Barysz matrix weighted by the ionization potential	2D matrix-based descriptors
13	SM11_EA(bo)	Spectral moment of order 11 from the edge adjacency matrix weighted by the bond order	Edge adjacency indices
14	F02[F-F]	Frequency of F - F at topological distance 2	2D Atom Pairs
15	SpMaxA_G	Normalized leading eigenvalue from the geometrical matrix	3D matrix-based descriptors
16	B02[F-Cl]	Presence/absence of F - Cl at topological distance 2	2D Atom Pairs
17	ATSC6i	Centered Broto-Moreau autocorrelation of lag 6 weighted by ionization potential	2D autocorrelations
18	Mor19p	Signal 19 / weighted by polarizability	3D-MoRSE descriptors
19	MATS5v	Moran autocorrelation of lag 5 weighted by the van der Waals volume	2D autocorrelations
20	B10[F-Si]	Presence/absence of F - Si at topological	2D Atom Pairs

		distance 10	
21	JGI2	Mean topological charge index of order 2	2D autocorrelations
22	F10[O-B]	Frequency of O - B at topological distance 10	2D Atom Pairs
23	TDB06s	3D Topological distance based descriptors - lag 6 weighted by the I-state	3D autocorrelations
24	CATS3D_08_PN	CATS3D Positive-Negative BIN 08 (8.000 - 9.000 Å)	CATS 3D
25	Mor06i	Signal 06 / weighted by the ionization potential	3D-MoRSE descriptors
26	SsI	Sum of sI E-states	Atom-type E-state indices
27	Neoplastic-50	Ghose-Viswanadhan-Wendoloski antineoplastic-like index at 50%	Drug-like indices
28	SM3_Dz(Z)	Spectral moment of order 3 from Barysz matrix weighted by atomic number	2D matrix-based descriptors
29	CATS2D_06_AL	CATS2D Acceptor-Lipophilic at lag 06	CATS 2D
30	X3v	Valence connectivity index of order 3	Connectivity indices
31	Wi_B(m)	Wiener-like index from Burden matrix weighted by mass	2D matrix-based descriptors
32	SM3_Dz(v)	Spectral moment of order 3 from the Barysz matrix weighted by the van der Waals volume	2D matrix-based descriptors

Supplementary Data1. The datasets of the 1267 structurally diverse molecules labeled with 12 diverse colors and 5270 molecular descriptors.

Supplementary Data2. The datasets of the 598 structurally diverse molecules labeled with 12 diverse odors and 5270 molecular descriptors.

Supplementary Data3. The datasets of the 90 molecules with both color and odor information.

Figure1-4

Click here to download Figure Figure.pdf

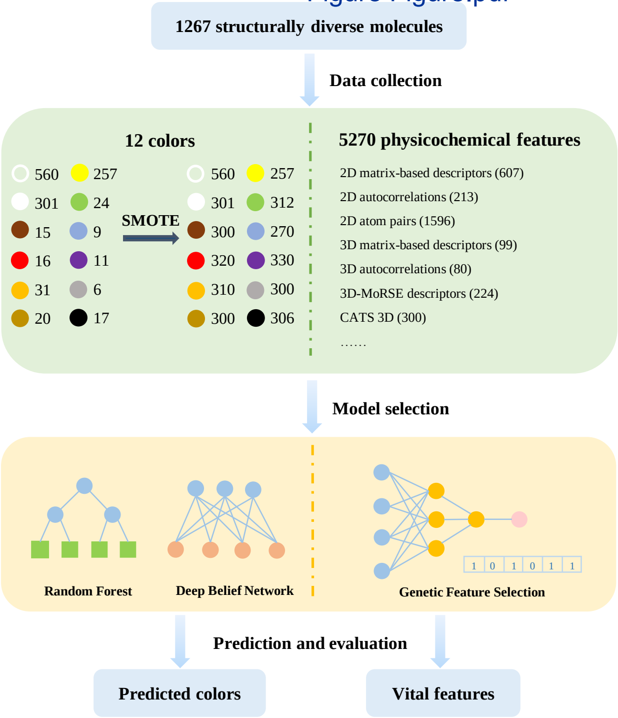

Figure 2

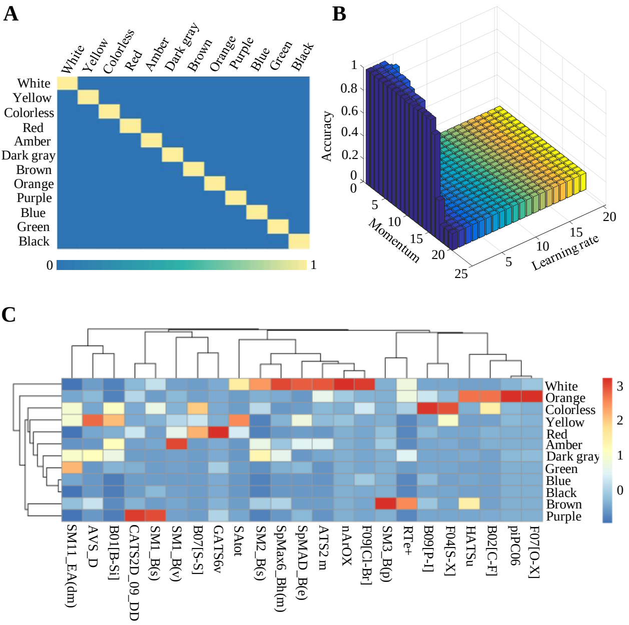
A
White
Yellow
Colorless
Red
Amber
Dark gray
Brown
Orange
Purple
Blue
Green
Black
0
1
B
Accuracy
Momentum
Learning rate
C
White
Orange
Colorless
Yellow
Red
Amber
Dark gray
Green
Blue
Black
Brown
Purple
SM11_EA(dm)
AVS_D
B01[B-Si]
CATS2D_09_DD
SM1_B(s)
SM1_B(v)
B07[S-S]
GATS6v
SAtot
SM2_B(s)
SpMax6_Bh(m)
SpMAD_B(e)
ATS2m
nArOX
F09[Cl-Br]
SM3_B(p)
RTe+
B09[P-I]
F04[S-X]
HATSu
B02[C-F]
piPC06
F07[O-X]

Figure 3

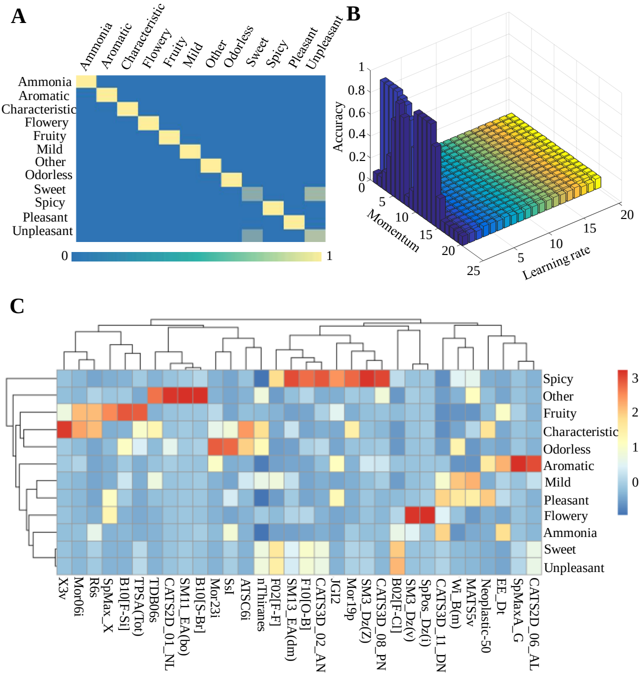

A
Ammonia
Aromatic
Characteristic
Flowery
Fruity
Mild
Other
Odorless
Sweet
Spicy
Pleasant
Unpleasant
0
1
B
Accuracy
Momentum
Learning rate
0
0.2
0.4
0.6
0.8
1
0
5
10
15
20
25
5
10
15
20
C
Spicy
Other
Fruity
Characteristic
Odorless
Aromatic
Mild
Pleasant
Flowery
Ammonia
Sweet
Unpleasant
3
2
1
0
X3v
Mor06i
R6s
SpMax_X
B10[F-Si]
TPSA(Tot)
TDB06s
CATS2D_01_NL
SM11_EA(bo)
B10[S-Br]
Mor23i
SsI
ATSC6i
nThiranes
F02[F-F]
SM13_EA(dm)
F10[O-B]
CATS3D_02_AN
JGI2
Mor19p
SM3_Dz(Z)
CATS3D_08_PN
B02[F-Cl]
SM3_Dz(v)
SpPos_Dz(i)
CATS3D_11_DN
Wi_B(m)
MATS5v
Neoplastic-50
EE_Dt
SpMaxA_G
CATS2D_06_AL

Figure 4

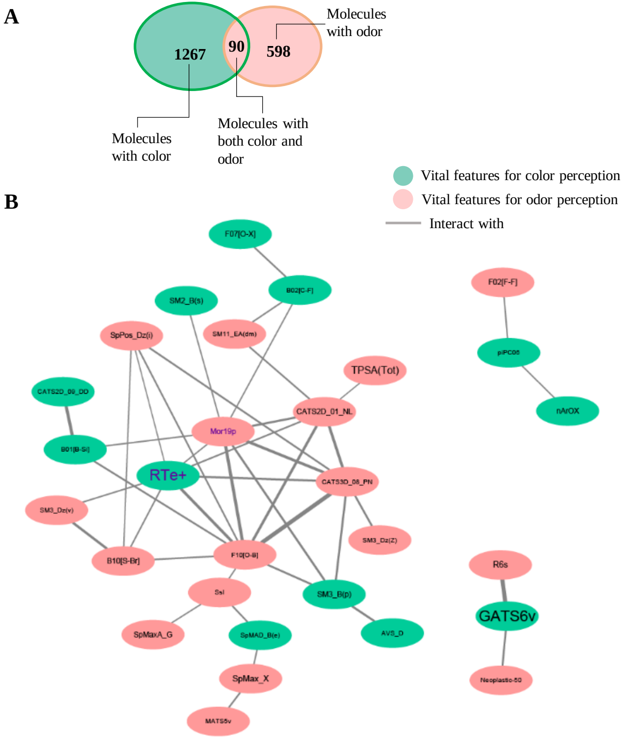
A
1267
90
598
Molecules with odor
Molecules with color
Molecules with both color and odor
B
Vital features for color perception
Vital features for odor perception
Interact with
F07[O-X]
B02[C-F]
SM2_B(s)
SpPos_Dz(i)
SM11_EA(dm)
TPSA(Tot)
CATS2D_09_DD
CATS2D_01_NL
Mor19p
B01[B-Si]
RTe+
CATS3D_08_PN
SM3_Dz(v)
SM3_Dz(Z)
B10[S-Br]
F10[O-B]
Ssl
SM3_B(p)
SpMaxA_G
SpMAD_B(e)
AVS_D
SpMax_X
MATS5v
F02[F-F]
piPC06
nArOX
R6s
GATS6v
Neoplastic-50

Click here to access/download
Supplementary Material
Supplementary Data1.xlsx

Click here to access/download
Supplementary Material
Supplementary Data2.xlsx

Supplementary Data3

Click here to access/download
Supplementary Material
Supplementary Data3.xlsx

Click here to download Personal Cover Cover Letter.pdf

SUN YAT-SEN UNIVERSITY

Haotian Lin, M.D., Ph.D.
State Key Laboratory of Ophthalmology
Zhongshan Ophthalmic Center
Sun Yat-sen University
Tel: +86-13802793086
E-mail: haot.lin@hotmail.com

Apr 8, 2019

Dear editors of *GigaScience*,

We would like to submit the enclosed manuscript entitled "Artificial intelligence deciphers codes for color and odor perceptions based on large-scale physicochemical features", for consideration of publication in *GigaScience*.

Color vision is indispensable in human life, and has become a premier model system for understanding how information is processed by neural circuits. In the past 20 years, the field of animal coloration research has been propelled forward by technological advances including spectrophotometry, digital imaging and computational neuroscience. However, the physicochemical basis of objects' color has not been explored completely, and how color information is integrated with information from odor is still unclear.

A previous research in *GigaScience* (Volume 7, Issue 2, February 2018, gix127, https://doi.org/10.1093/gigascience/gix127) equipped artificial intelligence with a "nose" to predict human olfactory perception from the chemoinformatic features of 476 molecules. Inspired by the study, we developed an accurate artificial intelligence platform distinguishing both color and odor based on over one million physicochemical features of different molecules, and validated key features for perception prediction based on machine learning techniques. The accuracy of distinguishing color was 100.0%. Furthermore, we factored out several key features interlink at the perception network that help explain why color and odor perceptions are interrelated.

We believe that both color and odor perceptions are closely connected with the physicochemical properties of the molecules, and our artificial intelligence platform can understand and navigate colors and odors in the real world. Our findings offer new insight into the understanding of objects' color, and lend a few clues on the investigations of relationships among neural circuits and perceptions.

In addition, we certify that the submission is an original work and is not under review by any other publication. We confirm that all co-authors have reviewed and agreed with the content of the manuscript, and there is no financial interest to report. If you have any questions or concerns regarding this manuscript, please do not hesitate to contact us. Thank you for your consideration.

Sincerely yours,

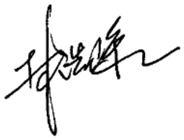

Haotian Lin on behalf of the authors
