# Supplementary material for: Artificial intelligence deciphers codes for color and odor perceptions based on large-scale chemoinformatic data
Source: Gigascience. 2020 Feb 26;9(2):giaa011. doi: 10.1093/gigascience/giaa011 (PMC7043059; doi:10.1093/gigascience/giaa011)
Supplement: giaa011_GIGA-D-19-00112_Revision_1 [file giaa011_giga-d-19-00112_revision_1.pdf]

GigaScience

Artificial intelligence deciphers codes for color and odor perceptions based on large-scale chemoinformatic data

--Manuscript Draft--

**Manuscript Number:**	GIGA-D-19-00112R1
**Full Title:**	Artificial intelligence deciphers codes for color and odor perceptions based on large-scale chemoinformatic data
**Article Type:**	Research
**Funding Information:**	National Key Research and Development Program of China (2018YFC0116500) — Prof. Haotian Lin<br>the Key Research and Development Program of Guangdong Province (No. 2018B010109008) — Prof. Haotian Lin<br>National Natural Science Foundation of China (81770967) — Prof. Haotian Lin<br>National Natural Science Foundation of China (81822010) — Prof. Haotian Lin
**Abstract:**	Background<br>Color vision is the ability to detect, distinguish, and analyze the wavelength distributions of light independent of the total intensity. It mediates the interaction between an organism and its environment from multiple important aspects. However, the physicochemical basis of color coding has not been explored completely, and how color perception is integrated with other sensory input, typically odor, is unclear.<br>Results<br>Here, we developed an artificial intelligence platform to train algorithms for distinguishing color and odor based on the large-scale physicochemical features of 1267 and 598 structurally diverse molecules, respectively. The predictive accuracies achieved using the random forest and deep belief network for the prediction of color were 100.0% ± 0.0% and 95.23% ± 0.40% (mean ± SD), respectively. The predictive accuracies achieved using the random forest and deep belief network for the prediction of odor were 93.40% ± 0.31% and 94.75% ± 0.44% (mean ± SD), respectively. Twenty-four physicochemical features were sufficient for the accurate prediction of color, while four physicochemical features were sufficient for the accurate prediction of odor. A positive correlation between the color coding and odor coding properties of the molecules was predicted. A group of descriptors was found to interlink prominently in color and odor perceptions.<br>Conclusions<br>Our random forest model and DBN accurately predicted the colors and odors of structurally diverse molecules. These findings extend our understanding of the molecular and structural basis of color vision and reveal the interrelationship between color and odor perceptions in nature.
**Corresponding Author:**	Haotian Lin, Ph.D., M.D.<br>Sun Yat-Sen University Zhongshan Ophthalmic Center<br>CHINA
**Corresponding Author Secondary Information:**	
**Corresponding Author's Institution:**	Sun Yat-Sen University Zhongshan Ophthalmic Center
**Corresponding Author's Secondary Institution:**	
**First Author:**	Xiayin Zhang
**First Author Secondary Information:**	
**Order of Authors:**	Xiayin Zhang

Powered by Editorial Manager® and ProduXion Manager® from Aries Systems Corporation

	Kai Zhang
	Duoru Lin
	Yi Zhu
	Chuan Chen
	Lin He
	Xusen Guo
	Kexin Chen
	Ruixin Wang
	Zhenzhen Liu
	Xiaohang Wu
	Erping Long
	Kai Huang
	Zhiqiang He
	Xiyang Liu
	Haotian Lin, Ph.D., M.D.
**Order of Authors Secondary Information:**	
**Response to Reviewers:**	Dear Scott Edmunds and Reviewers,<br><br>Thank you so much for the agreements and insightful suggestions on our manuscript. The following are our point-by-point responses to the reviewers’ comments and corresponding changes are marked in the revised manuscript. We hope that we have addressed all the suggestions adequately. Please let us know if you have any further questions or suggestions.<br><br>----------------------------------<br><br>Our point-by-point responses are as follows:<br><br>Reviewer #1:<br><br>Comment (1): The authors proposed machine learning approaches for predicting color and odor of a small molecule based on large-scale chemoinformatic features. They investigated the interplay between color and odor perception and found chemoinformatic features in predicting color and odor perception.<br>Key results and information are missing in this manuscript. None of the figure legends were provided. For example, in line 118, the authors claim "Using k-fold cross-validations (k = 4), the random forest model identified and utilized the most discriminative features with 100.00% ± 0.0% (mean ± SD) accuracy in the prediction of twelve colors (Figure 2A)." However, Figure 2A seems to be a heatmap across different colors, instead of prediction accuracy. Even if the authors refer to Figure 2B, it does not make any sense to me. What is the meaning of colors in Figure 2B? Is it the result for only one odor? I assume the "Momentum" and "Learning rate" are the parameters used in DBN, then where are the results using random forest? Where is the result for each fold in their 4-fold cross-validation? It is the same situation for odor prediction in Figure 3.<br>In sum, the authors really need solid evidence (e.g. shown in both box plots and supplementary tables) to support their claim of 100% and 89% accuracy in predicting color and odor.<br><br>Response: Thanks so much for your constructive comments and suggestions for our study. We strengthened and completed the key results and information for both color and odor prediction. All the figure legends were modified (Pages 21-22). We hope you find that we have addressed your concerns well.<br>To show our key results more clearly, we added boxplots presenting the results of color

and odor prediction using the random forest or DBN (Figure 2C, D; Figure 3C, D). At the same time, confusion matrixes were used to assist in observing the prediction results achieved using random forest (Figure 2A; Figure 3A), and column charts were used to assist in observing the prediction results achieved with DBN to represent the results of predicting all twelve colors or all twelve odors (Figure 2B; Figure 3B). In addition, the color of the column has been changed to be uniform.
The updated results for each fold in the 4-fold cross-validation are shown below. The table has been added to the Supplementary Materials (Table S3).

Comment (2): The experiment details are not clearly described. Based on the manuscript, the authors first used a strategy called SMOTE to over-sample the minority class and under-sample the majority class. Then they performed 4-fold cross validation. This may introduce overfitting to their study. For example, a molecule was oversampled and used twice in both model training and model testing during their cross validations. The correct way is partitioning the data into the training and testing data first, then oversampling. The authors need to clarify this.

Response: Thanks so much for your scrupulous correction. We agree that partitioning the data into training and testing data should come first, followed by oversampling. In the previous version, first we separated the data used for the 4-fold cross-validation and testing, and second, we completed the oversampling for training. The test data were not oversampled. In this version, we did not use any oversampling method according to your suggestion, and we clarified this point on Page 7, Lines 145-146.

Comment (3): The advantage of SMOTE is not clear. I suggest they compare the results of (1) SMOTE oversampling and (2) random oversampling.

Response: Thanks for your suggestion. We agree that the use of SMOTE oversampling needs further verification. In the review process, our results suggested that the accuracies achieved using direct classifications for the random forest and DBN (Table S3) were better than those achieved using SMOTE or random oversampling. Therefore, all the information about SMOTE oversampling has been removed.

Comment (4): The recent state-of-the-art method published in GigaScience ("Accurate prediction of personalized olfactory perception from large-scale chemoinformatic features.") was not discussed in this study. The author should compare with the previous method, or at least discuss the connections and differences between these studies.

Response: We are grateful for your recommendation. We have studied the best algorithm for olfaction prediction in the DREAM challenge and further discussed the connections and differences between the findings (Page 10, Lines 215-232).

Comment (5): The network architecture of deep belief network should be provided, including details such as number of layers, number of parameters.

Response: Many thanks for your comment. We compared three DBN structures for the prediction of either color or odor, and optimizations of the parameters of each structure have been conducted. The architecture that performed best for both color and odor prediction was the input layer with 5270 neurons and only one RBM with 5270 visible neurons and 500 hidden neurons. The moderate performance was achieved with the input layer with 5270 neurons and two RBMs. One RBM was composed of 5270 visible neurons and 2000 hidden neurons, and the other contained 2000 visible neurons and 500 hidden neurons. The worst performance was achieved with the input layer with 5270 neurons and three RBMs. One RBM contained 5270 visible neurons and 2000 hidden neurons, one was composed of 2000 visible neurons and 1000 hidden neurons, and the last contained 1000 visible neurons and 500 hidden neurons. Therefore, the best architecture was used in the follow-up prediction. We have also added these details on Page 13, Lines 284-295.

Reviewer #2:

Comment (1): The authors in their manuscript develop machine learning (random forest and DBN) trained models for distinguishing 12 distinct colours and 12 odours

Powered by Editorial Manager® and ProduXion Manager® from Aries Systems Corporation

based on large-scale physicochemical features of 1267 and 598 structurally diverse molecules, respectively. In this analysis, the authors discuss identified important features for a specific classification. Moreover, shows some connections between colours, and odour features. The manuscript is well written, made it easier to go through the content. However, some major issues are listed below should discuss or clarify in the manuscript.

Response: Thanks for your agreement on the merit and quality of our work. We also appreciate your constructive comments and have further discussed the major restrictions of our study (see the following responses).

Comment (2): In the data description section: line 90 - 99: the decision of selecting these specific colours and odour is missing. For example where these colour for particular molecules previously defined from NCBI or they visually identify the colours of the molecules or they used some software for this identification. The similar question arises for the odours. I think odours are very subjective to the person who is labelling the features. This should be mentioned in the data description.

Response: Thanks so much for your constructive suggestion. We agree that olfactory perception varies greatly among individuals. So we selected molecules with definite color or odors as defined by the NCBI.
A previous study of personalized olfactory perception published in GigaScience used a dataset of molecules sensed by 49 voluntary people [1]. They found that the perceived attributes including the intensity were rated differently among individuals, which considerably complicated the prediction challenge. We further discussed the connections and differences between these studies and emphasized the data from NCBI as our “gold standard” in the revised manuscript (Page 10, Lines 215-221).
1. Hongyang Li, Bharat Panwar, Gilbert S. Omenn & Yuanfang Guan. Accurate prediction of personalized olfactory perception from large-scale chemoinformatic features. GigaScience 2017; 7, 1–11.

Comment (3): line 111: replacing "NaN" with 0. I don't think the missing values should be treated this genitally. Unless all the missing values are because of one reason and the information is not needed for a particular molecule. The missing values in chemoinformatics dataset could be present because of various reasons, for example, the introduction of missing values is either no information was available (in literature/experiment etc) or due to the chemical calculation is not needed for this molecule. Both the cases can’t have the same output. This should be reflected in your dataset and influence the model prediction. Also, mention how much missing data is present in your dataset.

Response: Thanks for your suggestion. In our study, all the missing values are due to unavailable information in Dragon 7.0, which is the most used application for the calculation of molecular descriptors worldwide. The missing values simply mean that for the associated molecules, some descriptors have not been calculated for some reason, which commonly happens, as several descriptors have particular constraints (https://chm.kode-solutions.net/products_dragon_tutorial.php#01). In addition, our results of classification were quite good when substituting "NaN" with 0, indicating that these missing values did not play significant roles in the prediction modeling. However, we agree that new information is required to confirm our findings if an upgraded version of the Dragon software becomes available. The reasons behind and statistics of missing data have been added in the data description according to your suggestion (Page 7, Lines 138-145).

Comment (4): In Figure 1: it is unclear how odour dataset was included? Do you have two different workflows for colour and odour dataset?

Response: Thanks for your suggestion. We have rearranged Figure 1 to integrate the workflows of color and odor prediction.

Comment (5): I think the colour classification model is overestimating the prediction of the training dataset. For a clear understanding, you can report sensitivity, specificity, and F1 instead of accuracy, also because of accuracy paradox.

	Response: Many thanks for your comment. We are sorry that the sensitivity, specificity, and F1 which are regularly used for model evaluation in bi-classification were not fit for our study. Because both color and odor were divided into twelve categories, confusion matrixes were used to assist in observing the prediction effects of the random forest (Figure 2A, 3A), and column charts were used to assist in observing the prediction effects of the DBN (Figure 2B, 3B). To better evaluate our models of twelve-category classification, we added the kappa coefficient. Upon using all features to predict color, k = 1.0000 ± 0.0000 (mean ± SD) using the random forest, and k = 0.9400 ± 0.0030 (mean ± SD) using the DBN. Upon using all features to predict odor, k = 0.9232 ± 0.0037 (mean ± SD) using the random forest, and k = 0.9397 ± 0.0031 (mean ± SD) using the DBN. The kappa coefficients have been added in the results (Page7, Lines 152-153; Page8, Lines 177-178).<br><br>Comment (6): Figure legend is missing, which makes it hard to read and understand the figures.<br><br>Response: Thanks so much for your correction. All the figure legends were modified (Pages 21-22).<br><br>Comment (7): From figures and text, it is unclear if the random forest performed better than DBN? This is not the main findings of this manuscript, however, it is helpful to identify which method performs better for future prediction. The impression of Figure 1 also suggests that there will be a comparison between the random forest and DBN. The comparison, in terms of evaluation measure (false positives, false negative, F1 measure), should be mentioned in the main publication.<br><br>Response: Thanks for your suggestion. We agree that the comparison between the random forest and DBN should be completed. To show our key results more clearly, we added boxplots presenting the results of the random forest and DBN for color and odor prediction (Figure 2C, D; Figure 3C, D). The new results for each fold in the 4-fold cross-validation are shown below. The table has also been added to the Supplementary Materials (Table S3).<br>Overall, we found that the accuracy and kappa coefficient achieved using the random forest (100% ± 0.00%, 1.0000 ± 0.0000) were better than those achieved with the DBN (95.23% ± 0.40%, 0.9400 ± 0.0030) in color prediction with twelve categories. For odor prediction with twelve categories, the accuracy and kappa coefficient achieved using the DBN (94.75% ± 0.44%, 0.9397 ± 0.0031) were better than those achieved with the random forest (93.40% ± 0.31%, 0.9232 ± 0.0037). We further discussed the comparison in Page 10, Line 202-209. We are sorry that the sensitivity, specificity, and F1 which are regularly used for model evaluation in bi-classification, were not fit for our study.<br><br>Comment (8): Line 218, could you elaborate on how random forest can effectively avoid overfitting and deliver generalized knowledge? there is no evidence suggesting that random forest avoids overfitting. For some reference check this blog: https://mljar.com/blog/random-forest-overfitting/.<br><br>Response: Thanks for your scrupulous correction. We removed the statement “A random forest model can effectively avoid overfitting” to avoid potential controversies in the Method (Page 12, Lines 260-261).<br><br>Comment (9): The random forest can produce variable importance, out of curiosity, are the variable importance comparable to the genetic algorithm? I think this is an interesting part of your publication that can be discussed.<br><br>Response: Thanks for your suggestion. We agree that the comparison between the random forest and genetic algorithm could be very interesting. However, the dimensionality of the physicochemical data was very high with 5270 descriptors per molecule, and the data matrix was sparse in our study (Page 14, Lines 297-300). Many of the features are valued as “0” when calculated by the Dragon software, which means that they do not contribute to the classification (Page 14, Lines 304-306). Therefore, we preferred to combine the genetic algorithm and random forest algorithm, while the genetic algorithm was used for feature selection.

Powered by Editorial Manager® and ProduXion Manager® from Aries Systems Corporation

	Comment (10): Change heat map to heatmap in the publication.<br><br>Response: Thanks for your suggestion. We have changed the statement accordingly (Lines 164, 184).<br><br>Comment (11): Use the full form first before abbreviation.<br><br>Response: Thanks for your suggestion. We have made modifications to use the full form first. A list of abbreviations is presented on Page 16, Line 342-345.<br><br>--<br><br>Finally, thank you again for your acceptance and all of the helpful comments, and we hope that you will now find our revisions suitable for publication.<br><br>Sincerely yours,<br>Haotian Lin on behalf of all authors
**Additional Information:**	
**Question**	**Response**
Are you submitting this manuscript to a special series or article collection?	No
**Experimental design and statistics**<br><br>Full details of the experimental design and statistical methods used should be given in the Methods section, as detailed in our Minimum Standards Reporting Checklist. Information essential to interpreting the data presented should be made available in the figure legends.<br><br>Have you included all the information requested in your manuscript?	Yes
**Resources**<br><br>A description of all resources used, including antibodies, cell lines, animals and software tools, with enough information to allow them to be uniquely identified, should be included in the Methods section. Authors are strongly encouraged to cite Research Resource Identifiers (RRIDs) for antibodies, model organisms and tools, where possible.<br><br>Have you included the information requested as detailed in our Minimum Standards Reporting Checklist?	Yes

**Availability of data and materials**<br><br>All datasets and code on which the conclusions of the paper rely must be either included in your submission or deposited in publicly available repositories (where available and ethically appropriate), referencing such data using a unique identifier in the references and in the "Availability of Data and Materials" section of your manuscript.<br><br>Have you have met the above requirement as detailed in our Minimum Standards Reporting Checklist?	Yes

Manuscript (with changes tracked)
Click here to access/download;Manuscript;Manuscript (with changes tracked).docx
Click here to view linked References

Artificial intelligence deciphers codes for color and odor perceptions based on large-scale chemoinformatic data

Xiayin Zhang[1†] (zhangxiayin@gzzoc.com), Kai Zhang[1,2†] (hugo88315@163.com), Duoru Lin[1†] (linduoru@sina.com), Yi Zhu[1,3] (y.zhu17@med.miami.edu), Chuan Chen[1,3] (c.chen30@med.miami.edu), Lin He[2] (August_us@163.com), Xusen Guo[4] (guoxs3@mail2.sysu.edu.cn), Kexin Chen[1] (873490288@qq.com), Ruixin Wang[1] (ruiruiw413@aliyun.com), Zhenzhen Liu[1] (liu_zhenzhen@qq.com), Xiaohang Wu[1] (1034281949@qq.com), Erping Long[1] (longerping@qq.com), Kai Huang[4] (huangk36@mail.sysu.edu.cn), Zhiqiang He[5] (hezq@bupt.edu.cn), Xiyang Liu[2] (xyliu@xidian.edu.cn) and Haotian Lin[1,6*] (haot.lin@hotmail.com).

[1]State Key Laboratory of Ophthalmology, Zhongshan Ophthalmic Center, Sun Yat-sen University, Guangzhou 510060, China;

[2]School of Computer Science and Technology, Xidian University, Xi'an 710000, China;

[3]Department of Molecular and Cellular Pharmacology, University of Miami Miller School of Medicine, Miami, Florida 33136, USA;

[4]Key Laboratory of Machine Intelligence and Advanced Computing, Ministry of Education School of Data and Computer Science, Sun Yat-Sen University;

[5]Key Laboratory of Universal Wireless Communications, Beijing University of Posts and Telecommunications, Beijing 100876, China.

[6]Center of Precision Medicine, Sun Yat-sen University, Guangzhou 510080, China.

[†] These authors contributed equally to this work.

* **Corresponding Author:**

Prof. Haotian Lin

Xian Lie South Road 54#, Guangzhou, China, 510060

Telephone: +86-13802793086

Email address: haot.lin@hotmail.com

Abstract

Background

Color vision is the ability to detect, distinguish, and analyze the wavelength distributions of light independent of the total intensity. It mediates the interaction between an organism and its environment from multiple important aspects. However, the physicochemical basis of color coding has not been explored completely, and how color perception is integrated with other sensory input, typically odor, is unclear.

Results

Here, we developed an artificial intelligence platform to train algorithms for distinguishing color and odor based on the large-scale physicochemical features of 1267 and 598 structurally diverse molecules, respectively. The predictive accuracies achieved using the random forest and deep belief network for the prediction of color were 100.0% ± 0.0% and 95.23% ± 0.40% (mean ± SD), respectively. The predictive accuracies achieved using the random forest and deep belief network for the prediction of odor were 93.40% ± 0.31% and 94.75% ± 0.44% (mean ± SD), respectively. Twenty-four physicochemical features were sufficient for the accurate prediction of color, while four physicochemical features were sufficient for the accurate prediction of odor. A positive correlation between the color coding and odor coding properties of the molecules was predicted. A group of descriptors was found to interlink prominently in color and odor perceptions.

Conclusions

Our random forest model and DBN accurately predicted the colors and odors of

structurally diverse molecules. These findings extend our understanding of the molecular and structural basis of color vision and reveal the interrelationship between color and odor perceptions in nature.

Keywords: color perception; odor perception; random forest; deep belief network; physicochemical features.

Background

Color vision mediates the relationship between an organism and its environment in multiple important ways, including influencing mate choice, camouflage, and speciation [1]. We see a colorful world because different objects are composed of materials with different reflectance spectra in the wavelength range visible to our eyes [2]. Although knowledge of fundamental optical processes such as reflection, refraction, interference, diffraction, and scattering is accumulating [3], we lack the ability to recognize the color of cellular structure and pattern formation at optical scales from nanometers to microns.

Nature creates various colorful materials based on physicochemical properties including topological and geometrical properties that humans cannot easily see [4, 5]. For instance, the color changes from bright yellow through reddish–purple to blue when the size of a gold sample is decreased [6]. The different colors of disubstituted benzenes were discovered to be related to differences in the molecular structure with ortho, meta and para substitutions [7, 8]. The odors of chemicals are also fully encoded within their specific physicochemical properties [9, 10]. The compositions and structures of functional groups have been suggested to be crucial for the perception of aroma [11]. Moreover, evidence of the interaction between color vision and olfaction has been discovered [12]. For example, the odor of a host plant can modify the color sensed by a swallowtail butterfly [13]. The odor of wine can be predicted according to its color [14]. Additionally, the perceived intensity of an odor is positively correlated with the intensity of color [15, 16]. Neuroimaging and

repetitive transcranial magnetic stimulation studies showed that high-level odor processing also actives the visual cortex [17, 18]. However, the relationship between color and odor in terms of molecular physicochemical properties is largely unknown.

Artificial intelligence (AI) tools can be optimized to infer the innate laws of natural processes through machine learning tasks based on large-scale data sets and make predictions of the unknown [19, 20]. In the chemical sciences, AI has been used to guide chemical and material design, synthesis, characterization, and modeling [21, 22]. Previous researchers have equipped AI with a "nose" to predict human olfactory perception from the physicochemical features of 476 molecules and 21 perceptual attributes perceived by 49 individuals [23].

Here, we developed a random forest model and deep belief network (DBN) to predict the colors and odors of chemicals based on their molecular descriptors. We applied genetic algorithms for feature selection to identify the descriptors that contribute most to the predictive accuracies. In addition, we investigated the connection between the key physicochemical features in color and odor coding to unravel the commonality between visual and olfactory perception.

Data Description

Data collection and labeling. A total of 1267 structurally diverse molecules was used for color prediction in this study, and 598 structurally diverse molecules were used for odor prediction. The color, odor and three-dimensional (3D) structure data of these molecules were all collected from the key chemical information resource at the U.S.

National Center for Biotechnology Information, PubChem [24] (https://pubchem.ncbi.nlm.nih.gov) between June 1, 2017, and November 30, 2017. Molecules with definite colors or odors were defined from PubChem, and molecules with multiple colors or odors that are difficult to define were excluded. The data set of colors was classified into 12 diverse colors, including yellow (257 molecules), white (301 molecules), orange (31 molecules), red (16 molecules), purple (11 molecules), green (24 molecules), blue (9 molecules), brown (20 molecules), amber (15 molecules), gray (6 molecules), black (17 molecules) and colorless (560 molecules). The data set of odors was classified into 12 diverse odors, including ammonia (37 molecules), aromatic (36 molecules), characteristic (27 molecules), flower (19 molecules), fruity (29 molecules), mild (38 molecules), other (127 molecules), pleasant (16 molecules), unpleasant (23 molecules), spicy (54 molecules), sweet (30 molecules) and odorless (162 molecules).

Physicochemical features of the molecules. The PubChem compound identifier for each molecule was provided (Supplementary data). We applied the commercial chemoinformatics software package Dragon (version 7.0, https://chm.kode-solutions.net/products_dragon_papers.php) to calculate 5270 physicochemical descriptors for each of the molecules, including the simplest atom types, functional groups and fragment counts, topological and geometrical descriptors, 3D descriptors, several property estimations (such as log$P$) and drug-like and lead-like alerts (such as the Lipinski's alert). These molecular descriptors are formal mathematical representations of a molecule and include their definition, symbols and

labels, formulas, some numerical examples, data, and molecular graphs, as presented in the Handbook of Molecular Descriptors [25]. The missing values marked as “NaN” simply mean that for these molecules, some descriptors have not been calculated for some reason, which is common because several descriptors have particular constraints. Molecules with more than 2000 descriptors marked as “NaN” were not used. We replaced all of the “NaN” entries with “0” during the dataset preprocessing. For molecules with color, the average number of “NaN” within 5270 descriptors was 353 per molecule. For molecules with odor, the average number of “NaN” within 5270 descriptors was 28 per molecule. The data was divided into the training and testing data sets without oversampling. The overall workflow is shown in Figure 1.

Results

Color prediction

Random forest and DBN algorithms were applied for the *in silico* test. Using *k*-fold cross-validations (k = 4), the random forest model identified and utilized the most discriminative features with 100.00% ± 0.0% (mean ± SD) accuracy in the prediction of twelve colors (Figure 2A, C), with a kappa coefficient of 1.0000 ± 0.0000 (mean ± SD). As a type of probability generation model consisting of multiple restricted Boltzmann machines (RBMs), the DBN also performed excellently, with a predictive accuracy of 95.23% ± 0.40% (mean ± SD) (Figure 2B, D) and a kappa coefficient of 0.9400 ± 0.0030 (mean ± SD).

Key physicochemical features for color perception

The combination of the genetic algorithm and random forest algorithm enables us to estimate the importance of each molecular descriptor by permuting the values of the descriptors across samples and computing the increases in prediction errors. After running the genetic feature selection task 20 times, twenty-four descriptors were selected as the key physicochemical features with a classification accuracy of 99.45% ± 0.14% by using *k*-fold cross-validations (*k* = 4). The molecular descriptor "ATS6p" ranked first, followed by "B05[S-X]", "P_VSA_m_1" and "F01[C-S]". The heatmap of the hierarchical cluster analysis between the twenty-four key features and the twelve colors is shown in Figure 2E. "VR1_H2", "SssGeH2", "B05[S-X]", "SpPos_D", and "CATS2D_01_NL" were the main contributors to white, whereas "RDF025s" was the most important factor in predicting green. Information relevant to the key physicochemical features for color perception is reported in Table S1.

Distinction and connection with olfaction perception

We next applied the AI platform to predict odor perception based on physicochemical features. In total, 598 structurally diverse molecules were collected and classified into twelve diverse odors based on PubChem [24], including pleasant, unpleasant, ammonia, aromatic, flowery, fruity, spicy, sweet, mild, odorless, characteristic, and other. The accuracies of the odor prediction were 93.40% ± 0.31% for the random forest model using *k*-fold cross-validations (*k* = 4) (Figure 3A, C) and 94.75% ± 0.44% for the DBN (Figure 3B, D), with kappa coefficients of 0.9232 ± 0.0037 and 0.9397 ± 0.0031, respectively. After running the genetic feature selection task 20 times, thirty-nine descriptors were selected 16 times, with a classification accuracy of 93.38%

± 0.31% for the random forest model and 94.68% ± 0.67% for the DBN. Meanwhile, 12 descriptors and 4 descriptors were selected 17 times and 18 times respectively. Using the top 4 descriptors "Wi_Dz(Z)", "F01[Si-Si]", "F04[S-F]", and "G(I..I)", the DBN achieved the best accuracy of 96.40% ± 0.51% in odor prediction with twelve categories (Table S3). The heatmap of the hierarchical cluster analysis between the thirty-nine key features and the twelve odors is shown in Figure 3E. Information relevant to the key physicochemical features for odor perception is presented in Table S2.

To understand the correlation between color and odor, we collected 90 molecules with both color and odor information and analyzed the two groups using a chi-square test. The colors were divided into two categories (white, colorless/other), as were the odors (odorless/other). A correlation was predicted for both types of perception for these molecules (χ^2 = 17.445; $P < 0.001$). In the complex network of color and odor, more than fifty molecular descriptors were found to be interlinked prominently according to their correlation values (the absolute value of the Pearson correlation coefficients > 0.1958) (Figure 4). The "ATS6p" descriptor that ranked first in key features for color perception was closely connected with the key features for odor perception, including "NdsssAs", "B01[Br-X]" and "nR05".

Discussion

Clarifying the underlying mechanism of color vision is inherently challenging, as the cognitive process of color vision is multidimensional and includes crossover among

the morphology and function of the human visual system [26-28]. Here, we established a terse framework for distinguishing color without wavelengths based on only 24 physicochemical features. We found that the accuracy and kappa coefficient achieved using random forest (100% ± 0.00%, 1.0000 ± 0.0000) were better than those achieved with the DBN (95.23% ± 0.40%, 0.9400 ± 0.0030) in color prediction with twelve categories. For odor prediction with twelve categories, the accuracy and kappa coefficient achieved using the DBN (94.75% ± 0.44%, 0.9397 ± 0.0031) were better than those achieved with the random forest (93.40% ± 0.31%, 0.9232 ± 0.0037). Our findings also suggested that key physicochemical features in distinguishing color and odor are connected. The 2D autocorrelation descriptors and many other descriptors interlink at the network between color and odor perception, indicating that both color and odor perceptions are partially determined by the physicochemical properties of the molecules and that color and odor perceptions are closely interrelated.

Previous studies on predicting odor have been conducted by the DREAM Olfaction Prediction Challenge [23, 29]. A dataset of 476 molecules sensed by 49 voluntary people was applied, and the perceived attributes including the intensity were found to rate differently among the individuals, which considerably complicated the prediction challenge [29]. Our study collected a total of 598 structurally diverse molecules and classified them into twelve diverse odors based on PubChem to avoid a subjective effect on odor perception. The winning algorithm of the DREAM challenge, which was the best performer in predicting individual responses and the second-best

performer in predicting population responses in the challenge, indicated that the random forest outperforms other base learners (linear, ridge, and support vector machine) in predicting odor [29]. Based on their study, we added the DBN method and achieved better results in odor prediction with a classification accuracy of 94.75% ± 0.44% for 12 categories. Considering that the RBM can map features into higher feature space to make classification easier, the use of four features achieved the best classification accuracy of 96.40% ± 0.51%. In contrast, the random forest showed a higher accuracy in color prediction than DBN did. Above all, we believe that the machine learning method can be extended to predict other physicochemical properties.

In addition, odor sensing was found to be less accurate than that of color. Several factors may affect the accuracy of the AI in odor perception. First, odor perception is more subjective based on perceived biases, and it is challenging to confirm the number and character of its perceptual dimensions [30]. Defining a specific odor is especially difficult for human beings compared with other sensory modalities [31]. Second, the olfactory system involves high-dimensional input with attached arbitrary associations, whereas color vision occurs under predefined spatial conditions [12]. Thus, the processing demands of the two systems are not entirely consistent with each other. Third, the two systems employ different strategies in temporal coding to convey information. The olfactory system uses temporal coding to increase its representational capacity, while the visual system uses temporal coding to reduce the redundancy [12].

In this study, we add new insight into the decoding of color vision, but the controlling and tuning of these codes require further investigation. Inspired by the key physicochemical features involved in color prediction, researchers may be able to develop materials with vivid colors for potential applications in sensing technologies, security, light-emitting sources, and paints [32-34].

Potential implications

The ability to explain visual neural activities from the perspective of AI would also enable us to build an artificial vision system that could favorably stimulate the color vision of an individual. Once the perception process of human color vision is completely decoded, the AI platform may help in the design of artificial brain stimulation interfaces that can restore color vision and enable blind patients to “see” colors without biological eyes.

Methods

Random forest algorithm

Random forest is an ensemble learning method for regression and classification [35]. In a random forest model, each decision tree is built from a random sampling of samples and features, which can deliver generalized knowledge [35]. Furthermore, a random set of features is used to determine the best split at each node during the construction of a tree. Here, the dimensionality of the physicochemical data was high, with 5270 descriptors per molecule, and the perception data matrix was sparse. By averaging hundreds of trees in this work, the effects of outliers and noise were

reduced. The random forest parameter mTry (i.e., the number of input variables randomly chosen at each split) was set to 72 (square root of 5270 features), while the other random forest parameter nTree (i.e., the number of trees to grow for each forest) was set to 100. *k*-fold cross-validation ($k = 4$) was applied for the classification.

Deep belief network (DBN)

DBN is a type of probability generative model that consists of multiple RBMs. The superposition of multiple RBMs solves the training problem of multiple layered neural networks. The overall training process of the DBN includes two stages: a pretraining stage and a fine-tuning stage [36]. 1) Pretraining stage: Each RBM includes a visual layer and a hidden layer. There are no interlayer connections between the visual layer and hidden layer. After training the first RBM, the activation value of the hidden layer of the first RBM is input into the visual layer of the second RBM. 2) Fine-tuning stage: With the help of the BP neural network that resides after the last RBM and the chain rule of derivation, the DBN will be trained as a whole neural network. In this study, the input of the DBN is the vector consisting of 5270 molecular descriptors. During the first stage of the DBN, the dimensions of the vector are compressed. During the second stage, the compressed vector can be used for classification.

We compared three DBN structures for the prediction of either color or odor, and optimizations of the parameters of each structure were conducted. The architecture that performed best in both color and odor prediction was the input layer with 5270 neurons and only one RBM with 5270 visible neurons and 500 hidden neurons. The

moderate performance was achieved with the input layer with 5270 neurons and two RBMs. One RBM was composed of 5270 visible neurons and 2000 hidden neurons, and the other contained 2000 visible neurons and 500 hidden neurons. The worst performance was achieved with the input layer with 5270 neurons and three RBMs. One RBM contained 5270 visible neurons and 2000 hidden neurons, one was composed of 2000 visible neurons and 1000 hidden neurons, and the last contained 1000 visible neurons and 500 hidden neurons. Therefore, the best architecture was used in the follow-up prediction.

Genetic algorithms for feature selection

Because the high dimensionality of the feature vector of the molecules leads to difficulties in distinguishing which features are helpful in classification, the genetic algorithm [37,38] and random forest algorithm were combined so that the important features could be selected in this study. Genetic algorithms designed for feature selection can implement feature selection and classification processes simultaneously. In this study, the accuracy of the random forest was adopted as the fitness evaluation function of the genetic algorithm. The chromosome coding method was binary coding, and the length of the chromosome was equal to the dimension of the feature vector. A value of “0” signifies that the feature corresponding to this bit is not needed in the classification; otherwise, the feature is needed in the classification. Because of the randomness of the genetic algorithm, the experiment was conducted 20 times. All of the attributes chosen through genetic feature selection for use in color and odor prediction were converted into z-scores, and the relationship between each pair of

attributes was evaluated by the Pearson correlation coefficient. The cutoff for the weights was chosen between -0.3 and 0.3.

With the features selected from the genetic algorithm, feature ranking was performed to study which attributes were more important for classification. In this process, for a feature A_i in the feature set $\{A_1, A_2 \ldots A_n\}$, the validating accuracy for the original validation dataset is *acc1*. The validatiom accuracy obtained with the random permutation of A_i is *acc2*. $|acc2\text{-}acc1|$ is an indicator used to measure the importance of A_i. Then, all features are compared with this indicator. Because of the randomness of the random forest, this process was conducted 20 times.

Hierarchical clustering

Hierarchical approaches have the ability to simultaneously uncover multiple layers of a clustering structure [39]. The R heatmap package was used for clustering in this study.

Statistical analysis

The data were collected using the Qualtrics Web-based questionnaire package and analyzed using IBM SPSS Statistics version 24.

Availability of Supporting Data and Materials

All methods were implemented with MATLAB R2016a on HP Z420 workstation with Intel Xeon CPU E5-1620 v2@ 3.70GHZ and 16GB RAM. The operating system is Windows 7. Data corresponding to the molecules used in this study are presented in

Supplementary Data1-3. The source code of this study is presented in https://github.com/Hugo0512/ColorOdorprediction.

Additional Files

Table S1. Attribute importance ranking of color.

Table S2. Attribute importance ranking of odor.

Table S3. The results for each fold in the 4-fold cross-validation.

Supplementary Data1. The datasets of the 1267 structurally diverse molecules labeled with 12 diverse colors and 5270 molecular descriptors.

Supplementary Data2. The datasets of the 598 structurally diverse molecules labeled with 12 diverse odors and 5270 molecular descriptors.

Supplementary Data3. The datasets of the 90 molecules with both color and odor information.

Abbreviations

3D, Three dimensional; AI, Artificial intelligence; DBN, Deep belief network; Dragon, Software for the calculation of molecular descriptors; GETAWAY, Geometry, topology and atom-weights assembly; RBM, Restricted Boltzmann machine.

Completing interests

The authors declare that they have no competing interests.

Funding

This study was funded by the National Key R&D Program of China (2018YFC0116500), the Key Research and Development Program of Guangdong Province (No. 2018B010109008), the National Natural Science Foundation of China

(81770967, 81822010). The funders had no role in the study design, data collection, and analysis, the decision to publish or the preparation of the manuscript.

Author contributions

H.T.L., X.Y.Z. and D.R.L. conceived and designed the prediction algorithm, K.Z., X.Y.Z. and D.R.L. were responsible for data management and performing the computational analyses. R.X.W., Z.Z.L., X.H.W., and E.P.L. analyzed the discriminative features and prepared the figures. H.T.L., X.Y.Z. and D.R.L. contributed to the writing of the manuscript. Z.Y., C.C., L.H., X.S.G., K.X.C., K.H., X.Y.L., and Z.Q.H. contributed to the critical review of the study, and all authors read and approved the final manuscript.

Acknowledgments

We thank Xiaoming Chen (School Of Chemistry, Sun Yat-sen University) for reading, discussing and providing constructive comments for the manuscript.

References

1. Pete Vukusic & J. Roy Sambles. Photonic structures in biology. *Nature* 2003; 424, 852–855.

2. Le Chang, Pinglei Bao & Doris Y. Tsao. The representation of colored objects in macaque color patches. *Nature Communications* 2017; 8 (1).

3. S Kinoshita, S Yoshioka & J Miyazaki. Physics of structural colors. *Rep. Prog. Phys*; 2008, 71, 30pp.

4. Wilkinson, F.A. & Murillo, S.G. Advanced inorganic chemistry. 1988. Wiley.

5. McMurry, John. Organic chemistry. 2007. Brooks Cole.

6. Hallenbeck. Recent Advances in QSAR Studies. *Challenges & Advances in Computational Chemistry & Physics* 2010; 8, 31-32.

7. Paul, A. The use of nanocrystals in biological detection. *Nat Biotechnol* 2004; 22, 47-52.

8. Chen, F. & Gerion, D. Fluorescent CdSe/ZnS Nanocrystal−Peptide Conjugates for Long-term, Nontoxic Imaging and Nuclear Targeting in Living Cells. *Office of Scientific & Technical Information Technical Reports* 2004; 4, 1827-1832.

9. Rossiter, K.J. Structure−Odor Relationships. *Chemical ReviewsChemical ReviewsChem. Rev.* 1996; 96, 3201-3240.

10. Turin, L. A method for the calculation of odor character from molecular structure. *J Theor Biol* 2002; 216, 367-385.

11. Czerny, M., Brueckner, R., Kirchhoff, E., Schmitt, R. & Buettner, A. The influence of molecular structure on odor qualities and odor detection thresholds of volatile alkylated phenols. *Chem Senses* 2011; 36, 539.

12. Gire, D.H., *et al.* Temporal processing in the olfactory system: can we see a smell. *Neuron* 2013; 78, 416-432.

13. Yoshida, M., Itoh, Y., Ômura, H., Arikawa, K. & Kinoshita, M. Plant scents modify innate color preference in foraging swallowtail butterflies. *Biol Lett* 2015; 11.

14. Morrot, G., Brochet, F. & Dubourdieu, D. The Color of Odors. *Brain & Language* 2001; 79, 309-320.

15. Zellner, D.A. & Kautz, M.A. Color affects perceived odor intensity. *J Exp Psychol Hum Percept Perform* 1990; 16, 391-397.

16. Dubose, C.N., Cardello, A.V. & Maller, O. Effects of colorants and flavorants on

identification, perceived flavor and hedonic quality of fruit-flavored beverages and cake. *J Food Sci* 2010; 45, 1393-1399.

17. Royet, J.P., *et al.* Functional anatomy of perceptual and semantic processing for odors. *J Cogn Neurosci* 1999; 11, 94-109.

18. Jadauji, J.B., *et al.* Modulation of olfactory perception by visual cortex stimulation. *Journal of Neuroscience the Official Journal of the Society for Neuroscience* 2012; 32, 3095.

19. Gershman, S.J., Horvitz, E.J. & Tenenbaum, J.B. Computational rationality: A converging paradigm for intelligence in brains, minds, and machines. *Science* 2015; 349, 273-278.

20. Sanchez-Lengeling, B. & Aspuru-Guzik, A. Inverse molecular design using machine learning: Generative models for matter engineering. *Science* 2018; 361, 360-365.

21. Butler, K.T., Davies, D.W., Cartwright, H., Isayev, O. & Walsh, A. Machine learning for molecular and materials science. *Nature* 2018; 559, 547-555.

22. Paruzzo, F.M., *et al.* Chemical shifts in molecular solids by machine learning. *Nat Commun* 2018; 9, 4501.

23. Keller, A., *et al.* Predicting human olfactory perception from chemical features of odor molecules. *Science* 2017; 355, 820-826.

24. Kim, S., *et al.* PubChem Substance and Compound databases. *Nucleic Acids Res* 2016; 44, D1202-1213.

25. Todeschini, R. & Consonni, V. Handbook of Molecular Descriptors. 2000.

26. Solomon, S.G. & Lennie, P. The machinery of colour vision. *Nat Rev Neurosci* 2007; 8, 276-286.

27. Bennett, A. .D. & Théry, M. Avian Color Vision and Coloration: Multidisciplinary Evolutionary Biology. *Am Nat* 2007; 169, S1-1S6.

28. Kelber, A. & Osorio, D. From spectral information to animal colour vision: experiments and concepts. *Proceedings: Biological Sciences* 2010; 277, 1617-1625.

29. Hongyang Li, Bharat Panwar, Gilbert S. Omenn & Yuanfang Guan. Accurate prediction of personalized olfactory perception from large-scale chemoinformatic features. *GigaScience* 2017; 7, 1–11.

30. Kaeppler, K. & Mueller, F. Odor classification: a review of factors influencing perception-based odor arrangements. *Chem Senses* 2013; 38, 189-209.

31. Wippich, W., Mecklenbräuker, S. & Trouet, J. Implicit and explicit memories of odors. *Archiv Für Psychologie* 1989; 141, 195.

32. Hwang, J., *et al.* Electro-tunable optical diode based on photonic bandgap liquid-crystal heterojunctions. *Nat Mater* 2005; 4, 383-387.

33. Lee, H.S., Shim, T.S., Hwang, H., Yang, S.M. & Kim, S.H. Colloidal Photonic Crystals toward Structural Color Palettes for Security Materials. *Chemistry of Materials* 2013; 25, 2684-2690.

34. Sung Yeun, C., *et al.* Mesoporous bragg stack color tunable sensors. *Nano Lett* 2006; 6, 2456-2461.

35. Breiman, L. Random Forests. *Mach Learn* 2001; 45, 5-32.

36. Le, R.N. & Bengio, Y. Representational power of restricted boltzmann machines and deep belief networks. *Neural Comput* 2008; 20, 1631-1649.

37. Wang L, Zhang K, Liu X, et al. Comparative analysis of image classification methods for

automatic diagnosis of ophthalmic images. Scientific reports, 2017, 7: 41545.
38. Zhang K, Pan Q, Yu D, et al. Systemically modeling the relationship between climate change and wheat aphid abundance. Science of The Total Environment, 2019, 674: 392-400.
39. Eisen, M.B., *et al*. Cluster analysis and display of genome-wide expression patterns, 1998; 14863-14868.

Figures legends

Figure 1. The overall workflow of color prediction and odor prediction. 1267 structurally diverse molecules were labeled with 12 diverse colors, and 598 structurally diverse molecules were labeled with 12 diverse odors. In addition, 5270 physicochemical features of each molecule were generated by Dragon. Random forest models and deep belief networks were built to predict colors or odors using their physicochemical features. The models were evaluated based on the means and variances of the accuracies between the labeled and predicted colors or odors. Vital features were identified using genetic feature selection.

Figure 2. Color prediction using the random forest model and DBN. A. The confusion matrix for the classification of color with 100.00% accuracy by the random forest. The X-axis presents the labeled colors of the molecules, and the Y-axis presents the predicted colors of the molecules. B. The classification results for color were as high as 95.23% using the DBN. The *X*-axis presents the learning rate, the *Y*-axis presents the algorithm parameter “momentum”, and the *Z*-axis presents the accuracy rate. C. The boxplot presenting the accuracy of color prediction using the

random forest with all features or the top 24 features. D. The boxplot presenting the accuracy of color prediction using the DBN with all features or the top 24 features. E. The heatmap of the the correlation values between the twenty-four key features and the twelve colors based on the hierarchical clustering framework. The connections between the colors and descriptors were calculated by the Euclid distances.

Figure 3. Odor prediction using the random forest model and DBN. A. The confusion matrix for the classification of odor with 93.40% accuracy by the random forest. B. The classification results for odor were as high as 94.75% using the DBN. The *X*-axis presents the learning rate, the *Y*-axis presents the algorithm parameter “momentum”, and the *Z*-axis presents the accuracy rate. C. The boxplot to present the accuracy of color prediction using the random forest with all features or the top 39/12/4 features. D. The boxplot presenting the accuracy of color prediction using the DBN with all features or the top 39/12/4 features. E. The heatmap of the correlation values between the thirty-nine key features and the twelve odors based on the hierarchical clustering framework. Connections between the odors and descriptors were calculated by the Euclid distances.

Figure 4. The correlations between color and olfaction perception. A. Of the 1267 molecules with color, 90 also had odor information. B. Schematic diagram of the key physicochemical features for color and odor perceptions in the interactome. The key features for color perception were closely connected with the key features for odor perception. The distance of each line represents its correlation value.

Supplementary Materials

Table S1. Attribute importance ranking of color.

Ranking	Descriptor Name	Description	Block
1	ATS6p	Broto-Moreau autocorrelation of lag 6 (log function) weighted by polarizability	2D autocorrelations
2	B05[S-X]	Presence/absence of S - X at topological distance 5	2D Atom Pairs
3	P_VSA_m_1	P_VSA-like on mass, bin 1	P_VSA-like descriptor
4	F01[C-S]	Frequency of C - S at topological distance 1	2D Atom Pairs
5	RDF025s	Radial distribution function - 025 / weighted by I-state	RDF descriptors
6	Chi_D	Randic-like index from the topological distance matrix	2D matrix-based descriptors
7	HATSu	Leverage-weighted total index / unweighted	GETAWAY descriptors
8	VR1_H2	Randic-like eigenvector-based index from the reciprocal squared distance matrix	2D matrix-based descriptors
9	SpMax8_Bh(m)	Largest eigenvalue n. 8 of Burden matrix weighted by mass	Burden eigenvalues
10	CATS3D_19_DA	CATS3D Donor-Acceptor BIN 19 (19.000 - 20.000 Å)	CATS 3D
11	CATS3D_14_LL	CATS3D Lipophilic-Lipophilic BIN 14 (14.000 - 15.000 Å)	CATS 3D
12	EE_G	Estrada-like index (log function) from geometrical matrix	3D matrix-based descriptors
13	G(F..Br)	Sum of geometrical distances between F..Br	3D Atom Pairs
14	SssssBe-	Sum of ssssBe- E-states	Atom-type E-state indices
15	F09[Cl-Br]	Frequency of Cl - Br at topological distance 9	2D Atom Pairs
16	CATS3D_08_PL	CATS3D Positive-Lipophilic BIN 08 (8.000 - 9.000 Å)	CATS 3D
17	Mor28m	Signal 28 / weighted by mass	3D-MoRSE descriptors
18	CATS2D_01_NL	CATS2D Negative-Lipophilic at lag 01	CATS 2D
19	SssGeH2	Sum of ssGeH2 E-states	Atom-type E-state indices
20	SpPos_D	Spectral positive sum from topological distance matrix	2D matrix-based descriptors
21	HATS3i	Leverage-weighted autocorrelation of lag 3 /	GETAWAY

		weighted by ionization potential	descriptors
22	F05[N-I]	Frequency of N - I at topological distance 5	2D Atom Pairs
23	SM04_AEA(dm)	Spectral moment of order 4 from augmented edge adjacency mat. weighted by the dipole moment	Edge adjacency indices
24	TDB05i	3D Topological distance based descriptors - lag 5 weighted by ionization potential	3D autocorrelations

486 **Table S2. Attribute importance ranking of odor.**

Ranking	Descriptor Name	Description	Block
1	Wi_Dz(Z)	Wiener-like index from Barysz matrix weighted by atomic number	2D matrix-based descriptors
2	F01[Si-Si]	Frequency of Si - Si at topological distance 1	2D Atom Pairs
3	F04[S-F]	Frequency of S - F at topological distance 4	2D Atom Pairs
4	G(I..I)	Sum of geometrical distances between I..I	3D Atom Pairs
5	AVS_B(i)	Average vertex sum from Burden matrix weighted by ionization potential	2D matrix-based descriptors
6	F02[O-I]	Frequency of O - I at topological distance 2	2D Atom Pairs
7	nCIC	Number of rings (cyclomatic number)	Ring descriptors
8	RDF090i	Radial distribution function - 090 / weighted by ionization potential	RDF descriptors
9	nCconjX	Number of X on exo-conjugated C	Functional group counts
10	B10[Cl-Br]	Presence/absence of Cl - Br at topological distance 10	2D Atom Pairs
11	F07[O-X]	Frequency of O - X at topological distance 7	2D Atom Pairs
12	nR05	Number of 5-membered rings	Ring descriptors
13	H1e	H autocorrelation of lag 1 / weighted by Sanderson electronegativity	GETAWAY descriptors
14	NdsssAs	Number of atoms of type dsssAs	Atom-type E-state indices
15	B03[Br-X]	Presence/absence of Br - X at topological distance 3	2D Atom Pairs
16	B04[S-Si]	Presence/absence of S - Si at topological distance 4	2D Atom Pairs
17	B08[Cl-Si]	Presence/absence of Cl - Si at topological distance 8	2D Atom Pairs
18	VE3_Dz(p)	The logarithmic coefficient sum of the last eigenvector (absolute values) from Barysz matrix weighted by polarizability	2D matrix-based descriptors
19	VE1_Dz(i)	The coefficient sum of the last eigenvector (absolute values) from Barysz matrix weighted by ionization potential	2D matrix-based descriptors
20	B01[Br-X]	Presence/absence of Br - X at topological distance 1	2D Atom Pairs
21	B04[I-Si]	Presence/absence of I - Si at topological distance 4	2D Atom Pairs
22	CATS3D_18_DA	CATS3D Donor-Acceptor BIN 18 (18.000 - 19.000 Å)	CATS 3D

23	piPC03	Molecular multiple path count of order 3	Walk and path counts
24	ATS7s	Broto-Moreau autocorrelation of lag 7 (log function) weighted by I-state	2D autocorrelations
25	Eig14_EA(bo)	Eigenvalue n. 14 from edge adjacency mat. weighted by bond order	Edge adjacency indices
26	SM5_G	Spectral moment of order 5 from a geometrical matrix	3D matrix-based descriptors
27	Mor12i	Signal 12 / weighted by ionization potential	3D-MoRSE descriptors
28	nArC=N	Number of imines (aromatic)	Functional group counts
29	Br-091	Br attached to C1(sp3)	Atom-centered fragments
30	B07[O-P]	Presence/absence of O - P at topological distance 7	2D Atom Pairs
31	B10[B-Si]	Presence/absence of B - Si at topological distance 10	2D Atom Pairs
32	F03[I-B]	Frequency of I - B at topological distance 3	2D Atom Pairs
33	F09[F-F]	Frequency of F - F at topological distance 9	2D Atom Pairs
34	CATS3D_01_DD	CATS3D Donor-Donor BIN 01 (1.000 - 2.000 Å)	CATS 3D
35	Eta_beta_A	Eta average VEM count	ETA indices
36	RDF040i	Radial distribution function - 040 / weighted by ionization potential	RDF descriptors
37	B08[C-P]	Presence/absence of C - P at topological distance 8	2D Atom Pairs
38	F06[F-X]	Frequency of F - X at topological distance 6	2D Atom Pairs
39	CATS3D_01_DP	CATS3D Donor-Positive BIN 01 (1.000 - 2.000 Å)	CATS 3D

Table S3. The results for each fold in the 4-fold cross-validation.

Task	Method	Number of features	Mean accuracy	Accuracy (4-fold cross-validation)
Color perception	Random forest	All features	100%	100%, 100%, 100%, 100%
		24 features	99.45%	99.37%, 99.69%, 99.36%, 99.37%
	DBN	All features	95.23%	95.89%, 94.93%, 94.88%, 95.23%
		24 features	94.68%	95.24%, 95.33%, 94.46%, 93.67%
Odor perception	Random forest	All features	93.40%	93.33%, 93.92%, 93.20%, 93.15%
		39 features	93.38%	93.33%, 93.90%, 93.18%, 93.11%
		12 features	86.81%	86.00%, 86.49%, 87.07%, 87.67%
		4 features	66.07%	68.67, 63.51%, 61.22%, 71.23%
	DBN	All features	94.75%	95.24%, 94.35%, 94.27%, 95.13%
		39 features	94.68%	95.24%, 95.33%, 94.46% , 93.67%
		12 features	95.75%	95.53%, 95.67%, 96.03%, 95.78%
		4 features	96.40%	95.67%, 96.73%, 96.46%, 96.75%

Supplementary Data1. The datasets of the 1267 structurally diverse molecules labeled with 12 diverse colors and 5270 molecular descriptors.

Supplementary Data2. The datasets of the 598 structurally diverse molecules labeled with 12 diverse odors and 5270 molecular descriptors.

Supplementary Data3. The datasets of the 90 molecules with both color and odor information.

Manuscript (clean) Click here to access/download;Manuscript;Manuscript (clean).docx

Click here to view linked References

Artificial intelligence deciphers codes for color and odor perceptions based on large-scale chemoinformatic data

Xiayin Zhang[1†] (zhangxiayin@gzzoc.com), Kai Zhang[1,2†] (hugo88315@163.com), Duoru Lin[1†] (linduoru@sina.com), Yi Zhu[1,3] (y.zhu17@med.miami.edu), Chuan Chen[1,3] (c.chen30@med.miami.edu), Lin He[2] (August_us@163.com), Xusen Guo[4] (guoxs3@mail2.sysu.edu.cn), Kexin Chen[1] (873490288@qq.com), Ruixin Wang[1] (ruiruiw413@aliyun.com), Zhenzhen Liu[1] (liu_zhenzhen@qq.com), Xiaohang Wu[1] (1034281949@qq.com), Erping Long[1] (longerping@qq.com), Kai Huang[4] (huangk36@mail.sysu.edu.cn), Zhiqiang He[5] (hezq@bupt.edu.cn), Xiyang Liu[2] (xyliu@xidian.edu.cn) and Haotian Lin[1,6*] (haot.lin@hotmail.com).

[1]State Key Laboratory of Ophthalmology, Zhongshan Ophthalmic Center, Sun Yat-sen University, Guangzhou 510060, China;

[2]School of Computer Science and Technology, Xidian University, Xi'an 710000, China;

[3]Department of Molecular and Cellular Pharmacology, University of Miami Miller School of Medicine, Miami, Florida 33136, USA;

[4]Key Laboratory of Machine Intelligence and Advanced Computing, Ministry of Education School of Data and Computer Science, Sun Yat-Sen University;

[5]Key Laboratory of Universal Wireless Communications, Beijing University of Posts and Telecommunications, Beijing 100876, China.

[6]Center of Precision Medicine, Sun Yat-sen University, Guangzhou 510080, China.

[†] These authors contributed equally to this work.

* **Corresponding Author:**

Prof. Haotian Lin

Xian Lie South Road 54#, Guangzhou, China, 510060

Telephone: +86-13802793086

Email address: haot.lin@hotmail.com

Abstract

Background

Color vision is the ability to detect, distinguish, and analyze the wavelength distributions of light independent of the total intensity. It mediates the interaction between an organism and its environment from multiple important aspects. However, the physicochemical basis of color coding has not been explored completely, and how color perception is integrated with other sensory input, typically odor, is unclear.

Results

Here, we developed an artificial intelligence platform to train algorithms for distinguishing color and odor based on the large-scale physicochemical features of 1267 and 598 structurally diverse molecules, respectively. The predictive accuracies achieved using the random forest and deep belief network for the prediction of color were 100.0% ± 0.0% and 95.23% ± 0.40% (mean ± SD), respectively. The predictive accuracies achieved using the random forest and deep belief network for the prediction of odor were 93.40% ± 0.31% and 94.75% ± 0.44% (mean ± SD), respectively. Twenty-four physicochemical features were sufficient for the accurate prediction of color, while four physicochemical features were sufficient for the accurate prediction of odor. A positive correlation between the color coding and odor coding properties of the molecules was predicted. A group of descriptors was found to interlink prominently in color and odor perceptions.

Conclusions

Our random forest model and DBN accurately predicted the colors and odors of

structurally diverse molecules. These findings extend our understanding of the molecular and structural basis of color vision and reveal the interrelationship between color and odor perceptions in nature.

Keywords: color perception; odor perception; random forest; deep belief network; physicochemical features.

Background

Color vision mediates the relationship between an organism and its environment in multiple important ways, including influencing mate choice, camouflage, and speciation [1]. We see a colorful world because different objects are composed of materials with different reflectance spectra in the wavelength range visible to our eyes [2]. Although knowledge of fundamental optical processes such as reflection, refraction, interference, diffraction, and scattering is accumulating [3], we lack the ability to recognize the color of cellular structure and pattern formation at optical scales from nanometers to microns.

Nature creates various colorful materials based on physicochemical properties including topological and geometrical properties that humans cannot easily see [4, 5]. For instance, the color changes from bright yellow through reddish–purple to blue when the size of a gold sample is decreased [6]. The different colors of disubstituted benzenes were discovered to be related to differences in the molecular structure with ortho, meta and para substitutions [7, 8]. The odors of chemicals are also fully encoded within their specific physicochemical properties [9, 10]. The compositions and structures of functional groups have been suggested to be crucial for the perception of aroma [11]. Moreover, evidence of the interaction between color vision and olfaction has been discovered [12]. For example, the odor of a host plant can modify the color sensed by a swallowtail butterfly [13]. The odor of wine can be predicted according to its color [14]. Additionally, the perceived intensity of an odor is positively correlated with the intensity of color [15, 16]. Neuroimaging and

repetitive transcranial magnetic stimulation studies showed that high-level odor processing also actives the visual cortex [17, 18]. However, the relationship between color and odor in terms of molecular physicochemical properties is largely unknown.

Artificial intelligence (AI) tools can be optimized to infer the innate laws of natural processes through machine learning tasks based on large-scale data sets and make predictions of the unknown [19, 20]. In the chemical sciences, AI has been used to guide chemical and material design, synthesis, characterization, and modeling [21, 22]. Previous researchers have equipped AI with a "nose" to predict human olfactory perception from the physicochemical features of 476 molecules and 21 perceptual attributes perceived by 49 individuals [23].

Here, we developed a random forest model and deep belief network (DBN) to predict the colors and odors of chemicals based on their molecular descriptors. We applied genetic algorithms for feature selection to identify the descriptors that contribute most to the predictive accuracies. In addition, we investigated the connection between the key physicochemical features in color and odor coding to unravel the commonality between visual and olfactory perception.

Data Description

Data collection and labeling. A total of 1267 structurally diverse molecules was used for color prediction in this study, and 598 structurally diverse molecules were used for odor prediction. The color, odor and three-dimensional (3D) structure data of these molecules were all collected from the key chemical information resource at the U.S.

National Center for Biotechnology Information, PubChem [24] (https://pubchem.ncbi.nlm.nih.gov) between June 1, 2017, and November 30, 2017. Molecules with definite colors or odors were defined from PubChem, and molecules with multiple colors or odors that are difficult to define were excluded. The data set of colors was classified into 12 diverse colors, including yellow (257 molecules), white (301 molecules), orange (31 molecules), red (16 molecules), purple (11 molecules), green (24 molecules), blue (9 molecules), brown (20 molecules), amber (15 molecules), gray (6 molecules), black (17 molecules) and colorless (560 molecules). The data set of odors was classified into 12 diverse odors, including ammonia (37 molecules), aromatic (36 molecules), characteristic (27 molecules), flower (19 molecules), fruity (29 molecules), mild (38 molecules), other (127 molecules), pleasant (16 molecules), unpleasant (23 molecules), spicy (54 molecules), sweet (30 molecules) and odorless (162 molecules).

Physicochemical features of the molecules. The PubChem compound identifier for each molecule was provided (Supplementary data). We applied the commercial chemoinformatics software package Dragon (version 7.0, https://chm.kode-solutions.net/products_dragon_papers.php) to calculate 5270 physicochemical descriptors for each of the molecules, including the simplest atom types, functional groups and fragment counts, topological and geometrical descriptors, 3D descriptors, several property estimations (such as log$P$) and drug-like and lead-like alerts (such as the Lipinski's alert). These molecular descriptors are formal mathematical representations of a molecule and include their definition, symbols and

labels, formulas, some numerical examples, data, and molecular graphs, as presented in the Handbook of Molecular Descriptors [25]. The missing values marked as "NaN" simply mean that for these molecules, some descriptors have not been calculated for some reason, which is common because several descriptors have particular constraints. Molecules with more than 2000 descriptors marked as "NaN" were not used. We replaced all of the "NaN" entries with "0" during the dataset preprocessing. For molecules with color, the average number of "NaN" within 5270 descriptors was 353 per molecule. For molecules with odor, the average number of "NaN" within 5270 descriptors was 28 per molecule. The data was divided into the training and testing data sets without oversampling. The overall workflow is shown in Figure 1.

Results

Color prediction

Random forest and DBN algorithms were applied for the *in silico* test. Using *k*-fold cross-validations ($k = 4$), the random forest model identified and utilized the most discriminative features with 100.00% ± 0.0% (mean ± SD) accuracy in the prediction of twelve colors (Figure 2A, C), with a kappa coefficient of 1.0000 ± 0.0000 (mean ± SD). As a type of probability generation model consisting of multiple restricted Boltzmann machines (RBMs), the DBN also performed excellently, with a predictive accuracy of 95.23% ± 0.40% (mean ± SD) (Figure 2B, D) and a kappa coefficient of 0.9400 ± 0.0030 (mean ± SD).

Key physicochemical features for color perception

The combination of the genetic algorithm and random forest algorithm enables us to estimate the importance of each molecular descriptor by permuting the values of the descriptors across samples and computing the increases in prediction errors. After running the genetic feature selection task 20 times, twenty-four descriptors were selected as the key physicochemical features with a classification accuracy of 99.45% ± 0.14% by using *k*-fold cross-validations (*k* = 4). The molecular descriptor "ATS6p" ranked first, followed by "B05[S-X]", "P_VSA_m_1" and "F01[C-S]". The heatmap of the hierarchical cluster analysis between the twenty-four key features and the twelve colors is shown in Figure 2E. "VR1_H2", "SssGeH2", "B05[S-X]", "SpPos_D", and "CATS2D_01_NL" were the main contributors to white, whereas "RDF025s" was the most important factor in predicting green. Information relevant to the key physicochemical features for color perception is reported in Table S1.

Distinction and connection with olfaction perception

We next applied the AI platform to predict odor perception based on physicochemical features. In total, 598 structurally diverse molecules were collected and classified into twelve diverse odors based on PubChem [24], including pleasant, unpleasant, ammonia, aromatic, flowery, fruity, spicy, sweet, mild, odorless, characteristic, and other. The accuracies of the odor prediction were 93.40% ± 0.31% for the random forest model using *k*-fold cross-validations (*k* = 4) (Figure 3A, C) and 94.75% ± 0.44% for the DBN (Figure 3B, D), with kappa coefficients of 0.9232 ± 0.0037 and 0.9397 ± 0.0031, respectively. After running the genetic feature selection task 20 times, thirty-nine descriptors were selected 16 times, with a classification accuracy of 93.38%

± 0.31% for the random forest model and 94.68% ± 0.67% for the DBN. Meanwhile, 12 descriptors and 4 descriptors were selected 17 times and 18 times respectively. Using the top 4 descriptors "Wi_Dz(Z)", "F01[Si-Si]", "F04[S-F]", and "G(I..I)", the DBN achieved the best accuracy of 96.40% ± 0.51% in odor prediction with twelve categories (Table S3). The heatmap of the hierarchical cluster analysis between the thirty-nine key features and the twelve odors is shown in Figure 3E. Information relevant to the key physicochemical features for odor perception is presented in Table S2.

To understand the correlation between color and odor, we collected 90 molecules with both color and odor information and analyzed the two groups using a chi-square test. The colors were divided into two categories (white, colorless/other), as were the odors (odorless/other). A correlation was predicted for both types of perception for these molecules (χ^2 = 17.445; P < 0.001). In the complex network of color and odor, more than fifty molecular descriptors were found to be interlinked prominently according to their correlation values (the absolute value of the Pearson correlation coefficients > 0.1958) (Figure 4). The "ATS6p" descriptor that ranked first in key features for color perception was closely connected with the key features for odor perception, including "NdsssAs", "B01[Br-X]" and "nR05".

Discussion

Clarifying the underlying mechanism of color vision is inherently challenging, as the cognitive process of color vision is multidimensional and includes crossover among

the morphology and function of the human visual system [26-28]. Here, we established a terse framework for distinguishing color without wavelengths based on only 24 physicochemical features. We found that the accuracy and kappa coefficient achieved using random forest (100% ± 0.00%, 1.0000 ± 0.0000) were better than those achieved with the DBN (95.23% ± 0.40%, 0.9400 ± 0.0030) in color prediction with twelve categories. For odor prediction with twelve categories, the accuracy and kappa coefficient achieved using the DBN (94.75% ± 0.44%, 0.9397 ± 0.0031) were better than those achieved with the random forest (93.40% ± 0.31%, 0.9232 ± 0.0037). Our findings also suggested that key physicochemical features in distinguishing color and odor are connected. The 2D autocorrelation descriptors and many other descriptors interlink at the network between color and odor perception, indicating that both color and odor perceptions are partially determined by the physicochemical properties of the molecules and that color and odor perceptions are closely interrelated.

Previous studies on predicting odor have been conducted by the DREAM Olfaction Prediction Challenge [23, 29]. A dataset of 476 molecules sensed by 49 voluntary people was applied, and the perceived attributes including the intensity were found to rate differently among the individuals, which considerably complicated the prediction challenge [29]. Our study collected a total of 598 structurally diverse molecules and classified them into twelve diverse odors based on PubChem to avoid a subjective effect on odor perception. The winning algorithm of the DREAM challenge, which was the best performer in predicting individual responses and the second-best

performer in predicting population responses in the challenge, indicated that the random forest outperforms other base learners (linear, ridge, and support vector machine) in predicting odor [29]. Based on their study, we added the DBN method and achieved better results in odor prediction with a classification accuracy of 94.75% ± 0.44% for 12 categories. Considering that the RBM can map features into higher feature space to make classification easier, the use of four features achieved the best classification accuracy of 96.40% ± 0.51%. In contrast, the random forest showed a higher accuracy in color prediction than DBN did. Above all, we believe that the machine learning method can be extended to predict other physicochemical properties.

In addition, odor sensing was found to be less accurate than that of color. Several factors may affect the accuracy of the AI in odor perception. First, odor perception is more subjective based on perceived biases, and it is challenging to confirm the number and character of its perceptual dimensions [30]. Defining a specific odor is especially difficult for human beings compared with other sensory modalities [31]. Second, the olfactory system involves high-dimensional input with attached arbitrary associations, whereas color vision occurs under predefined spatial conditions [12]. Thus, the processing demands of the two systems are not entirely consistent with each other. Third, the two systems employ different strategies in temporal coding to convey information. The olfactory system uses temporal coding to increase its representational capacity, while the visual system uses temporal coding to reduce the redundancy [12].

In this study, we add new insight into the decoding of color vision, but the controlling and tuning of these codes require further investigation. Inspired by the key physicochemical features involved in color prediction, researchers may be able to develop materials with vivid colors for potential applications in sensing technologies, security, light-emitting sources, and paints [32-34].

Potential implications

The ability to explain visual neural activities from the perspective of AI would also enable us to build an artificial vision system that could favorably stimulate the color vision of an individual. Once the perception process of human color vision is completely decoded, the AI platform may help in the design of artificial brain stimulation interfaces that can restore color vision and enable blind patients to "see" colors without biological eyes.

Methods

Random forest algorithm

Random forest is an ensemble learning method for regression and classification [35]. In a random forest model, each decision tree is built from a random sampling of samples and features, which can deliver generalized knowledge [35]. Furthermore, a random set of features is used to determine the best split at each node during the construction of a tree. Here, the dimensionality of the physicochemical data was high, with 5270 descriptors per molecule, and the perception data matrix was sparse. By averaging hundreds of trees in this work, the effects of outliers and noise were

reduced. The random forest parameter mTry (i.e., the number of input variables randomly chosen at each split) was set to 72 (square root of 5270 features), while the other random forest parameter nTree (i.e., the number of trees to grow for each forest) was set to 100. *k*-fold cross-validation (*k* = 4) was applied for the classification.

Deep belief network (DBN)

DBN is a type of probability generative model that consists of multiple RBMs. The superposition of multiple RBMs solves the training problem of multiple layered neural networks. The overall training process of the DBN includes two stages: a pretraining stage and a fine-tuning stage [36]. 1) Pretraining stage: Each RBM includes a visual layer and a hidden layer. There are no interlayer connections between the visual layer and hidden layer. After training the first RBM, the activation value of the hidden layer of the first RBM is input into the visual layer of the second RBM. 2) Fine-tuning stage: With the help of the BP neural network that resides after the last RBM and the chain rule of derivation, the DBN will be trained as a whole neural network. In this study, the input of the DBN is the vector consisting of 5270 molecular descriptors. During the first stage of the DBN, the dimensions of the vector are compressed. During the second stage, the compressed vector can be used for classification.

We compared three DBN structures for the prediction of either color or odor, and optimizations of the parameters of each structure were conducted. The architecture that performed best in both color and odor prediction was the input layer with 5270 neurons and only one RBM with 5270 visible neurons and 500 hidden neurons. The

moderate performance was achieved with the input layer with 5270 neurons and two RBMs. One RBM was composed of 5270 visible neurons and 2000 hidden neurons, and the other contained 2000 visible neurons and 500 hidden neurons. The worst performance was achieved with the input layer with 5270 neurons and three RBMs. One RBM contained 5270 visible neurons and 2000 hidden neurons, one was composed of 2000 visible neurons and 1000 hidden neurons, and the last contained 1000 visible neurons and 500 hidden neurons. Therefore, the best architecture was used in the follow-up prediction.

Genetic algorithms for feature selection

Because the high dimensionality of the feature vector of the molecules leads to difficulties in distinguishing which features are helpful in classification, the genetic algorithm [37,38] and random forest algorithm were combined so that the important features could be selected in this study. Genetic algorithms designed for feature selection can implement feature selection and classification processes simultaneously. In this study, the accuracy of the random forest was adopted as the fitness evaluation function of the genetic algorithm. The chromosome coding method was binary coding, and the length of the chromosome was equal to the dimension of the feature vector. A value of “0” signifies that the feature corresponding to this bit is not needed in the classification; otherwise, the feature is needed in the classification. Because of the randomness of the genetic algorithm, the experiment was conducted 20 times. All of the attributes chosen through genetic feature selection for use in color and odor prediction were converted into z-scores, and the relationship between each pair of

attributes was evaluated by the Pearson correlation coefficient. The cutoff for the weights was chosen between -0.3 and 0.3.

With the features selected from the genetic algorithm, feature ranking was performed to study which attributes were more important for classification. In this process, for a feature A_i in the feature set $\{A_{1,} A_{2\ldots} A_n\}$, the validating accuracy for the original validation dataset is *acc1*. The validatiom accuracy obtained with the random permutation of A_i is *acc2*. |*acc2-acc1*| is an indicator used to measure the importance of A_i. Then, all features are compared with this indicator. Because of the randomness of the random forest, this process was conducted 20 times.

Hierarchical clustering

Hierarchical approaches have the ability to simultaneously uncover multiple layers of a clustering structure [39]. The R heatmap package was used for clustering in this study.

Statistical analysis

The data were collected using the Qualtrics Web-based questionnaire package and analyzed using IBM SPSS Statistics version 24.

Availability of Supporting Data and Materials

All methods were implemented with MATLAB R2016a on HP Z420 workstation with Intel Xeon CPU E5-1620 v2@ 3.70GHZ and 16GB RAM. The operating system is Windows 7. Data corresponding to the molecules used in this study are presented in

Supplementary Data1-3. The source code of this study is presented in https://github.com/Hugo0512/ColorOdorprediction.

Additional Files

Table S1. Attribute importance ranking of color.

Table S2. Attribute importance ranking of odor.

Table S3. The results for each fold in the 4-fold cross-validation.

Supplementary Data1. The datasets of the 1267 structurally diverse molecules labeled with 12 diverse colors and 5270 molecular descriptors.

Supplementary Data2. The datasets of the 598 structurally diverse molecules labeled with 12 diverse odors and 5270 molecular descriptors.

Supplementary Data3. The datasets of the 90 molecules with both color and odor information.

Abbreviations

3D, Three dimensional; AI, Artificial intelligence; DBN, Deep belief network; Dragon, Software for the calculation of molecular descriptors; GETAWAY, Geometry, topology and atom-weights assembly; RBM, Restricted Boltzmann machine.

Completing interests

The authors declare that they have no competing interests.

Funding

This study was funded by the National Key R&D Program of China (2018YFC0116500), the Key Research and Development Program of Guangdong Province (No. 2018B010109008), the National Natural Science Foundation of China

(81770967, 81822010). The funders had no role in the study design, data collection, and analysis, the decision to publish or the preparation of the manuscript.

Author contributions

H.T.L., X.Y.Z. and D.R.L. conceived and designed the prediction algorithm, K.Z., X.Y.Z. and D.R.L. were responsible for data management and performing the computational analyses. R.X.W., Z.Z.L., X.H.W., and E.P.L. analyzed the discriminative features and prepared the figures. H.T.L., X.Y.Z. and D.R.L. contributed to the writing of the manuscript. Z.Y., C.C., L.H., X.S.G., K.X.C., K.H., X.Y.L., and Z.Q.H. contributed to the critical review of the study, and all authors read and approved the final manuscript.

Acknowledgments

We thank Xiaoming Chen (School Of Chemistry, Sun Yat-sen University) for reading, discussing and providing constructive comments for the manuscript.

References

1. Pete Vukusic & J. Roy Sambles. Photonic structures in biology. *Nature* 2003; 424, 852–855.

2. Le Chang, Pinglei Bao & Doris Y. Tsao. The representation of colored objects in macaque color patches. *Nature Communications* 2017; 8 (1).

3. S Kinoshita, S Yoshioka & J Miyazaki. Physics of structural colors. *Rep. Prog. Phys*; 2008, 71, 30pp.

4. Wilkinson, F.A. & Murillo, S.G. Advanced inorganic chemistry. 1988. Wiley.

5. McMurry, John. Organic chemistry. 2007. Brooks Cole.

6. Hallenbeck. Recent Advances in QSAR Studies. *Challenges & Advances in Computational Chemistry & Physics* 2010; 8, 31-32.

7. Paul, A. The use of nanocrystals in biological detection. *Nat Biotechnol* 2004; 22, 47-52.

8. Chen, F. & Gerion, D. Fluorescent CdSe/ZnS Nanocrystal−Peptide Conjugates for Long-term, Nontoxic Imaging and Nuclear Targeting in Living Cells. *Office of Scientific & Technical Information Technical Reports* 2004; 4, 1827-1832.

9. Rossiter, K.J. Structure−Odor Relationships. *Chemical ReviewsChemical ReviewsChem. Rev.* 1996; 96, 3201-3240.

10. Turin, L. A method for the calculation of odor character from molecular structure. *J Theor Biol* 2002; 216, 367-385.

11. Czerny, M., Brueckner, R., Kirchhoff, E., Schmitt, R. & Buettner, A. The influence of molecular structure on odor qualities and odor detection thresholds of volatile alkylated phenols. *Chem Senses* 2011; 36, 539.

12. Gire, D.H., *et al.* Temporal processing in the olfactory system: can we see a smell. *Neuron* 2013; 78, 416-432.

13. Yoshida, M., Itoh, Y., Ômura, H., Arikawa, K. & Kinoshita, M. Plant scents modify innate color preference in foraging swallowtail butterflies. *Biol Lett* 2015; 11.

14. Morrot, G., Brochet, F. & Dubourdieu, D. The Color of Odors. *Brain & Language* 2001; 79, 309-320.

15. Zellner, D.A. & Kautz, M.A. Color affects perceived odor intensity. *J Exp Psychol Hum Percept Perform* 1990; 16, 391-397.

16. Dubose, C.N., Cardello, A.V. & Maller, O. Effects of colorants and flavorants on

identification, perceived flavor and hedonic quality of fruit-flavored beverages and cake. *J Food Sci* 2010; 45, 1393-1399.

17. Royet, J.P., *et al*. Functional anatomy of perceptual and semantic processing for odors. *J Cogn Neurosci* 1999; 11, 94-109.

18. Jadauji, J.B., *et al.* Modulation of olfactory perception by visual cortex stimulation. *Journal of Neuroscience the Official Journal of the Society for Neuroscience* 2012; 32, 3095.

19. Gershman, S.J., Horvitz, E.J. & Tenenbaum, J.B. Computational rationality: A converging paradigm for intelligence in brains, minds, and machines. *Science* 2015; 349, 273-278.

20. Sanchez-Lengeling, B. & Aspuru-Guzik, A. Inverse molecular design using machine learning: Generative models for matter engineering. *Science* 2018; 361, 360-365.

21. Butler, K.T., Davies, D.W., Cartwright, H., Isayev, O. & Walsh, A. Machine learning for molecular and materials science. *Nature* 2018; 559, 547-555.

22. Paruzzo, F.M., *et al.* Chemical shifts in molecular solids by machine learning. *Nat Commun* 2018; 9, 4501.

23. Keller, A., *et al.* Predicting human olfactory perception from chemical features of odor molecules. *Science* 2017; 355, 820-826.

24. Kim, S., *et al.* PubChem Substance and Compound databases. *Nucleic Acids Res* 2016; 44, D1202-1213.

25. Todeschini, R. & Consonni, V. Handbook of Molecular Descriptors. 2000.

26. Solomon, S.G. & Lennie, P. The machinery of colour vision. *Nat Rev Neurosci* 2007; 8, 276-286.

27. Bennett, A. .D. & Théry, M. Avian Color Vision and Coloration: Multidisciplinary Evolutionary Biology. *Am Nat* 2007; 169, S1-1S6.

28. Kelber, A. & Osorio, D. From spectral information to animal colour vision: experiments and concepts. *Proceedings: Biological Sciences* 2010; 277, 1617-1625.

29. Hongyang Li, Bharat Panwar, Gilbert S. Omenn & Yuanfang Guan. Accurate prediction of personalized olfactory perception from large-scale chemoinformatic features. *GigaScience* 2017; 7, 1–11.

30. Kaeppler, K. & Mueller, F. Odor classification: a review of factors influencing perception-based odor arrangements. *Chem Senses* 2013; 38, 189-209.

31. Wippich, W., Mecklenbräuker, S. & Trouet, J. Implicit and explicit memories of odors. *Archiv Für Psychologie* 1989; 141, 195.

32. Hwang, J., *et al.* Electro-tunable optical diode based on photonic bandgap liquid-crystal heterojunctions. *Nat Mater* 2005; 4, 383-387.

33. Lee, H.S., Shim, T.S., Hwang, H., Yang, S.M. & Kim, S.H. Colloidal Photonic Crystals toward Structural Color Palettes for Security Materials. *Chemistry of Materials* 2013; 25, 2684-2690.

34. Sung Yeun, C., *et al.* Mesoporous bragg stack color tunable sensors. *Nano Lett* 2006; 6, 2456-2461.

35. Breiman, L. Random Forests. *Mach Learn* 2001; 45, 5-32.

36. Le, R.N. & Bengio, Y. Representational power of restricted boltzmann machines and deep belief networks. *Neural Comput* 2008; 20, 1631-1649.

37. Wang L, Zhang K, Liu X, et al. Comparative analysis of image classification methods for

automatic diagnosis of ophthalmic images. Scientific reports, 2017, 7: 41545.

38. Zhang K, Pan Q, Yu D, et al. Systemically modeling the relationship between climate change and wheat aphid abundance. Science of The Total Environment, 2019, 674: 392-400.

39. Eisen, M.B., *et al.* Cluster analysis and display of genome-wide expression patterns, 1998; 14863-14868.

Figures legends

Figure 1. The overall workflow of color prediction and odor prediction. 1267 structurally diverse molecules were labeled with 12 diverse colors, and 598 structurally diverse molecules were labeled with 12 diverse odors. In addition, 5270 physicochemical features of each molecule were generated by Dragon. Random forest models and deep belief networks were built to predict colors or odors using their physicochemical features. The models were evaluated based on the means and variances of the accuracies between the labeled and predicted colors or odors. Vital features were identified using genetic feature selection.

Figure 2. Color prediction using the random forest model and DBN. A. The confusion matrix for the classification of color with 100.00% accuracy by the random forest. The X-axis presents the labeled colors of the molecules, and the Y-axis presents the predicted colors of the molecules. B. The classification results for color were as high as 95.23% using the DBN. The *X*-axis presents the learning rate, the *Y*-axis presents the algorithm parameter “momentum”, and the *Z*-axis presents the accuracy rate. C. The boxplot presenting the accuracy of color prediction using the

random forest with all features or the top 24 features. D. The boxplot presenting the accuracy of color prediction using the DBN with all features or the top 24 features. E. The heatmap of the the correlation values between the twenty-four key features and the twelve colors based on the hierarchical clustering framework. The connections between the colors and descriptors were calculated by the Euclid distances.

Figure 3. Odor prediction using the random forest model and DBN. A. The confusion matrix for the classification of odor with 93.40% accuracy by the random forest. B. The classification results for odor were as high as 94.75% using the DBN. The *X*-axis presents the learning rate, the *Y*-axis presents the algorithm parameter "momentum", and the *Z*-axis presents the accuracy rate. C. The boxplot to present the accuracy of color prediction using the random forest with all features or the top 39/12/4 features. D. The boxplot presenting the accuracy of color prediction using the DBN with all features or the top 39/12/4 features. E. The heatmap of the correlation values between the thirty-nine key features and the twelve odors based on the hierarchical clustering framework. Connections between the odors and descriptors were calculated by the Euclid distances.

Figure 4. The correlations between color and olfaction perception. A. Of the 1267 molecules with color, 90 also had odor information. B. Schematic diagram of the key physicochemical features for color and odor perceptions in the interactome. The key features for color perception were closely connected with the key features for odor perception. The distance of each line represents its correlation value.

Supplementary Materials

Table S1. Attribute importance ranking of color.

Ranking	Descriptor Name	Description	Block
1	ATS6p	Broto-Moreau autocorrelation of lag 6 (log function) weighted by polarizability	2D autocorrelations
2	B05[S-X]	Presence/absence of S - X at topological distance 5	2D Atom Pairs
3	P_VSA_m_1	P_VSA-like on mass, bin 1	P_VSA-like descriptor
4	F01[C-S]	Frequency of C - S at topological distance 1	2D Atom Pairs
5	RDF025s	Radial distribution function - 025 / weighted by I-state	RDF descriptors
6	Chi_D	Randic-like index from the topological distance matrix	2D matrix-based descriptors
7	HATSu	Leverage-weighted total index / unweighted	GETAWAY descriptors
8	VR1_H2	Randic-like eigenvector-based index from the reciprocal squared distance matrix	2D matrix-based descriptors
9	SpMax8_Bh(m)	Largest eigenvalue n. 8 of Burden matrix weighted by mass	Burden eigenvalues
10	CATS3D_19_DA	CATS3D Donor-Acceptor BIN 19 (19.000 - 20.000 Å)	CATS 3D
11	CATS3D_14_LL	CATS3D Lipophilic-Lipophilic BIN 14 (14.000 - 15.000 Å)	CATS 3D
12	EE_G	Estrada-like index (log function) from geometrical matrix	3D matrix-based descriptors
13	G(F..Br)	Sum of geometrical distances between F..Br	3D Atom Pairs
14	SssssBe-	Sum of ssssBe- E-states	Atom-type E-state indices
15	F09[Cl-Br]	Frequency of Cl - Br at topological distance 9	2D Atom Pairs
16	CATS3D_08_PL	CATS3D Positive-Lipophilic BIN 08 (8.000 - 9.000 Å)	CATS 3D
17	Mor28m	Signal 28 / weighted by mass	3D-MoRSE descriptors
18	CATS2D_01_NL	CATS2D Negative-Lipophilic at lag 01	CATS 2D
19	SssGeH2	Sum of ssGeH2 E-states	Atom-type E-state indices
20	SpPos_D	Spectral positive sum from topological distance matrix	2D matrix-based descriptors
21	HATS3i	Leverage-weighted autocorrelation of lag 3 /	GETAWAY

		weighted by ionization potential	descriptors
22	F05[N-I]	Frequency of N - I at topological distance 5	2D Atom Pairs
23	SM04_AEA(dm)	Spectral moment of order 4 from augmented edge adjacency mat. weighted by the dipole moment	Edge adjacency indices
24	TDB05i	3D Topological distance based descriptors - lag 5 weighted by ionization potential	3D autocorrelations

486 **Table S2. Attribute importance ranking of odor.**

Ranking	Descriptor Name	Description	Block
1	Wi_Dz(Z)	Wiener-like index from Barysz matrix weighted by atomic number	2D matrix-based descriptors
2	F01[Si-Si]	Frequency of Si - Si at topological distance 1	2D Atom Pairs
3	F04[S-F]	Frequency of S - F at topological distance 4	2D Atom Pairs
4	G(I..I)	Sum of geometrical distances between I..I	3D Atom Pairs
5	AVS_B(i)	Average vertex sum from Burden matrix weighted by ionization potential	2D matrix-based descriptors
6	F02[O-I]	Frequency of O - I at topological distance 2	2D Atom Pairs
7	nCIC	Number of rings (cyclomatic number)	Ring descriptors
8	RDF090i	Radial distribution function - 090 / weighted by ionization potential	RDF descriptors
9	nCconjX	Number of X on exo-conjugated C	Functional group counts
10	B10[Cl-Br]	Presence/absence of Cl - Br at topological distance 10	2D Atom Pairs
11	F07[O-X]	Frequency of O - X at topological distance 7	2D Atom Pairs
12	nR05	Number of 5-membered rings	Ring descriptors
13	H1e	H autocorrelation of lag 1 / weighted by Sanderson electronegativity	GETAWAY descriptors
14	NdsssAs	Number of atoms of type dsssAs	Atom-type E-state indices
15	B03[Br-X]	Presence/absence of Br - X at topological distance 3	2D Atom Pairs
16	B04[S-Si]	Presence/absence of S - Si at topological distance 4	2D Atom Pairs
17	B08[Cl-Si]	Presence/absence of Cl - Si at topological distance 8	2D Atom Pairs
18	VE3_Dz(p)	The logarithmic coefficient sum of the last eigenvector (absolute values) from Barysz matrix weighted by polarizability	2D matrix-based descriptors
19	VE1_Dz(i)	The coefficient sum of the last eigenvector (absolute values) from Barysz matrix weighted by ionization potential	2D matrix-based descriptors
20	B01[Br-X]	Presence/absence of Br - X at topological distance 1	2D Atom Pairs
21	B04[I-Si]	Presence/absence of I - Si at topological distance 4	2D Atom Pairs
22	CATS3D_18_DA	CATS3D Donor-Acceptor BIN 18 (18.000 - 19.000 Å)	CATS 3D

23	piPC03	Molecular multiple path count of order 3	Walk and path counts
24	ATS7s	Broto-Moreau autocorrelation of lag 7 (log function) weighted by I-state	2D autocorrelations
25	Eig14_EA(bo)	Eigenvalue n. 14 from edge adjacency mat. weighted by bond order	Edge adjacency indices
26	SM5_G	Spectral moment of order 5 from a geometrical matrix	3D matrix-based descriptors
27	Mor12i	Signal 12 / weighted by ionization potential	3D-MoRSE descriptors
28	nArC=N	Number of imines (aromatic)	Functional group counts
29	Br-091	Br attached to C1(sp3)	Atom-centered fragments
30	B07[O-P]	Presence/absence of O - P at topological distance 7	2D Atom Pairs
31	B10[B-Si]	Presence/absence of B - Si at topological distance 10	2D Atom Pairs
32	F03[I-B]	Frequency of I - B at topological distance 3	2D Atom Pairs
33	F09[F-F]	Frequency of F - F at topological distance 9	2D Atom Pairs
34	CATS3D_01_DD	CATS3D Donor-Donor BIN 01 (1.000 - 2.000 Å)	CATS 3D
35	Eta_beta_A	Eta average VEM count	ETA indices
36	RDF040i	Radial distribution function - 040 / weighted by ionization potential	RDF descriptors
37	B08[C-P]	Presence/absence of C - P at topological distance 8	2D Atom Pairs
38	F06[F-X]	Frequency of F - X at topological distance 6	2D Atom Pairs
39	CATS3D_01_DP	CATS3D Donor-Positive BIN 01 (1.000 - 2.000 Å)	CATS 3D

Table S3. The results for each fold in the 4-fold cross-validation.

Task	Method	Number of features	Mean accuracy	Accuracy (4-fold cross-validation)
Color perception	Random forest	All features	100%	100%, 100%, 100%, 100%
		24 features	99.45%	99.37%, 99.69%, 99.36%, 99.37%
	DBN	All features	95.23%	95.89%, 94.93%, 94.88%, 95.23%
		24 features	94.68%	95.24%, 95.33%, 94.46%, 93.67%
Odor perception	Random forest	All features	93.40%	93.33%, 93.92%, 93.20%, 93.15%
		39 features	93.38%	93.33%, 93.90%, 93.18%, 93.11%
		12 features	86.81%	86.00%, 86.49%, 87.07%, 87.67%
		4 features	66.07%	68.67, 63.51%, 61.22%, 71.23%
	DBN	All features	94.75%	95.24%, 94.35%, 94.27%, 95.13%
		39 features	94.68%	95.24%, 95.33%, 94.46% , 93.67%
		12 features	95.75%	95.53%, 95.67%, 96.03%, 95.78%
		4 features	96.40%	95.67%, 96.73%, 96.46%, 96.75%

Supplementary Data1. The datasets of the 1267 structurally diverse molecules labeled with 12 diverse colors and 5270 molecular descriptors.

Supplementary Data2. The datasets of the 598 structurally diverse molecules labeled with 12 diverse odors and 5270 molecular descriptors.

Supplementary Data3. The datasets of the 90 molecules with both color and odor information.

Figure 1

Click here to access/download;Figu

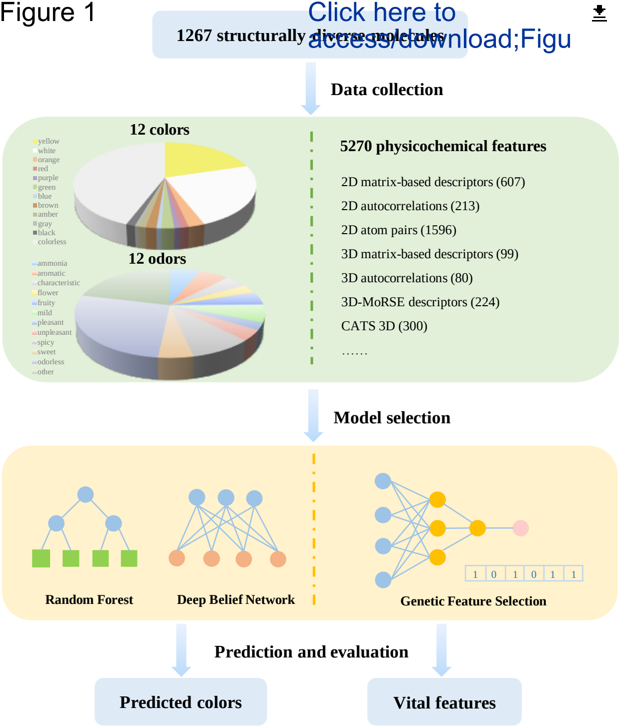

Figure 2

Click here to access/download;Figu

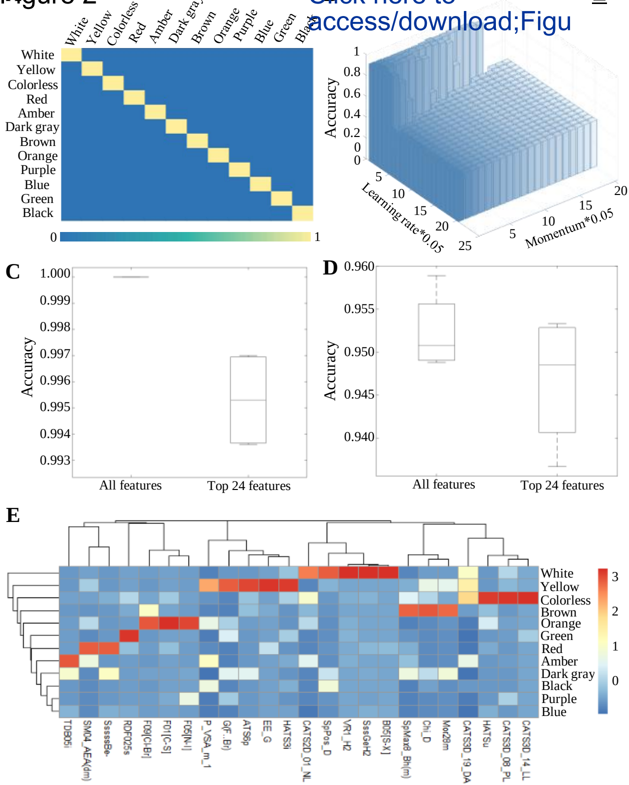

Figure 3

Click here to access/download;Figu

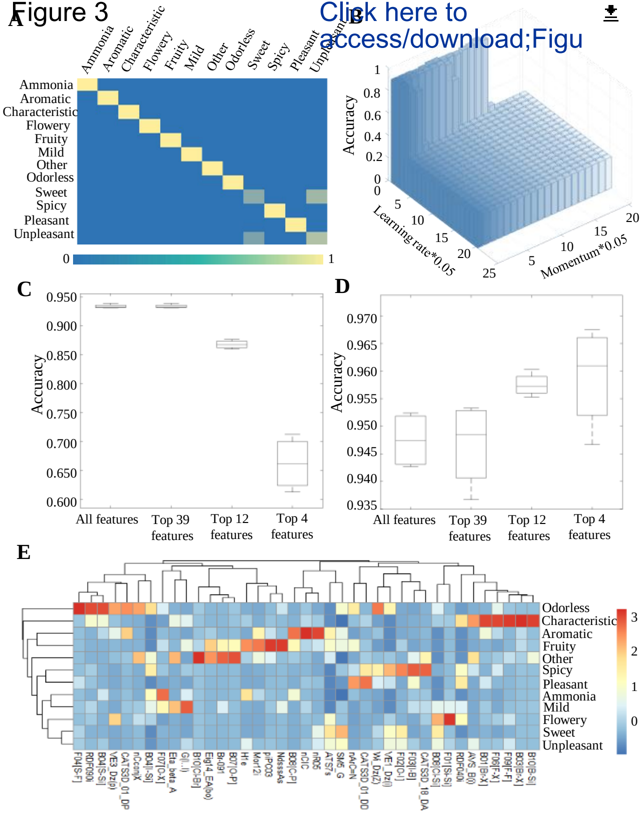

Figure 4

Click here to access/download;Figu

A

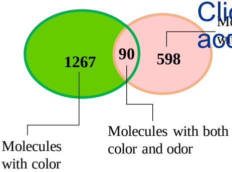

B

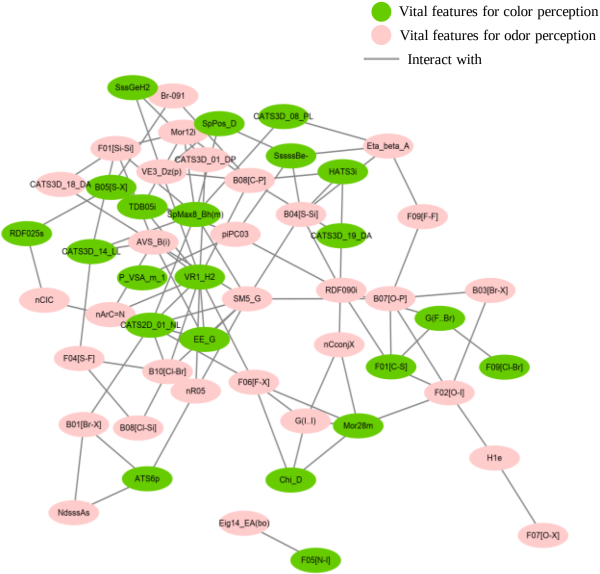

Supplementary Data1

Click here to access/download
Supplementary Material
Supplementary Data1.xlsx

Supplementary Data2

Click here to access/download
Supplementary Material
Supplementary Data2.xlsx

Supplementary Data3

Click here to access/download
Supplementary Material
Supplementary Data3.xlsx

Click here to access/download;Personal Cover;Revision Cover Letter.doc

中山大學
SUN YAT-SEN UNIVERSITY

Haotian Lin, M.D., Ph.D.
State Key Laboratory of Ophthalmology
Zhongshan Ophthalmic Center
Sun Yat-sen University
Tel: +86-13802793086
E-mail: haot.lin@hotmail.com

Oct 19, 2019

Dear Scott Edmunds,

Thank you so much for your decision letter on our manuscript entitled “Artificial intelligence deciphers codes for color and odor perceptions based on large-scale chemoinformatic data”. We sincerely treasure all the constructive and crucial suggestions from you and reviewers. We hope that you and the reviewers are interested in our study. Based on the provided suggestions and comments, we have addressed all the issues carefully and revised our manuscript accordingly. We hope that the revised manuscript can convince you and reviewers.

The main improvements and revisions are as follows:

1) The elaboration of the background was oriented to show the rationale and utility of color perception by AI for broader audiences (Page 4, Lines 73-80).
2) More rigorous and comprehensive comparisons were completed to present the results of color and odor prediction using the random forest or DBN (Page 10, Lines 201-208; Figure 2; Figure 3; Table S3).
3) Additional discussion of previous studies on odor prediction (Page 10, Lines 215-232), and improved explanation of the methods were added (Page 13, Lines 284-295, 297-300, 304-306).

In addition, we declare that our study did not involve any new software application. The source code of the algorithm is presented in https://github.com/Hugo0512/ColorOdorprediction. Data corresponding to the molecules used in this study are presented in Supplementary Data1-3.

Thank you once again for your agreement and help with our research. In the past 20 years, the field of animal color or odor perception has been propelled forward by technological advances including spectrophotometry and computational neuroscience. Our artificial intelligence platform can further help investigators understand and navigate colors and odors in the real world. We believe that our findings offer new insight into the understanding of objects’ colors and odors, and lend a few clues in the investigations of relationships among neural circuits and perceptions.

Sincerely yours,

Haotian Lin, on behalf of all authors

Response to Referees Letter
Click here to access/download;Personal Cover;Response to Referees Letter.docx

‘Response to Reviewers’ files GIGA-D-19-00112

Artificial intelligence deciphers codes for color and odor perceptions based on large-scale chemoinformatic data

GigaScience

Dear Scott Edmunds and Reviewers,

Thank you so much for the agreements and insightful suggestions on our manuscript. The following are our point-by-point responses to the reviewers’ comments and corresponding changes are marked in the revised manuscript. We hope that we have addressed all the suggestions adequately. Please let us know if you have any further questions or suggestions.

Our point-by-point responses are as follows:

Reviewer #1:

Comment (1): *The authors proposed machine learning approaches for predicting color and odor of a small molecule based on large-scale chemoinformatic features. They investigated the interplay between color and odor perception and found chemoinformatic features in predicting color and odor perception.*

Key results and information are missing in this manuscript. None of the figure legends were provided. For example, in line 118, the authors claim "Using k-fold cross-validations (k = 4), the random forest model identified and utilized the most discriminative features with 100.00% ± 0.0% (mean ± SD) accuracy in the prediction of twelve colors (Figure 2A)." However, Figure 2A seems to be a heatmap across different colors, instead of prediction accuracy. Even if the authors refer to Figure 2B, it does not make any sense to me. What is the meaning of colors in Figure 2B? Is it the result for only one odor? I assume the "Momentum" and "Learning rate" are the parameters used in DBN, then where are the results using random forest? Where is the result for each fold in their 4-fold cross-validation? It is the same situation for odor prediction in Figure 3.

In sum, the authors really need solid evidence (e.g. shown in both box plots and supplementary tables) to support their claim of 100% and 89% accuracy in predicting color and odor.

Response: Thanks so much for your constructive comments and suggestions for our study. We strengthened and completed the key results and information for both color and odor prediction. All the figure legends were modified (Pages 21-22). We hope you find that we have addressed your concerns well.

To show our key results more clearly, we added boxplots presenting the results of color and odor prediction using the random forest or DBN (Figure 2C, D; Figure 3C, D). At the same time, confusion matrixes were used to assist in observing the prediction results achieved using random forest (Figure 2A; Figure 3A), and column charts were used to assist in observing the prediction results achieved with DBN to represent the results of predicting all twelve colors or all twelve odors (Figure 2B; Figure 3B). In addition, the color of the column has been changed to be uniform.

The updated results for each fold in the 4-fold cross-validation are shown below. The table has been added to the Supplementary Materials (Table S3).

Table S3. The results for each fold in the 4-fold cross-validation.

Task	Method	Number of features	Mean accuracy	Accuracy (4-fold cross-validation)
Color perception	Random forest	All features	100%	100%, 100%, 100%, 100%
		24 features	99.45%	99.37%, 99.69%, 99.36%, 99.37%
	DBN	All features	95.23%	95.89%, 94.93%, 94.88%, 95.23%
		24 features	94.68%	95.24%, 95.33%, 94.46%, 93.67%
Odor perception	Random forest	All features	93.40%	93.33%, 93.92%, 93.20%, 93.15%
		39 features	93.38%	93.33%, 93.90%, 93.18%, 93.11%
		12 features	86.81%	86.00%, 86.49%, 87.07%, 87.67%
		4 features	66.07%	68.67, 63.51%, 61.22%, 71.23%
	DBN	All features	94.75%	95.24%, 94.35%, 94.27%, 95.13%
		39 features	94.68%	95.24%, 95.33%, 94.46% , 93.67%
		12 features	95.75%	95.53%, 95.67%, 96.03%, 95.78%
		4 features	96.40%	95.67%, 96.73%, 96.46%, 96.75%

Comment (2): *The experiment details are not clearly described. Based on the manuscript, the authors first used a strategy called SMOTE to over-sample the minority class and under-sample the majority class. Then they performed 4-fold cross validation. This may introduce overfitting to their study. For example, a molecule was oversampled and used twice in both model training and model testing during their cross validations. The correct way is partitioning the data into the training and testing data first, then oversampling. The authors need to clarify this.*

Response: Thanks so much for your scrupulous correction. We agree that partitioning the data into training and testing data should come first, followed by oversampling. In the previous version, first we separated the data used for the 4-fold cross-validation and testing, and second, we completed the oversampling for training. The test data were not oversampled. In this version, we did not use any oversampling method according to your suggestion, and we clarified this point on Page 7, Lines 145-146.

Comment (3): *The advantage of SMOTE is not clear. I suggest they compare the results of (1) SMOTE oversampling and (2) random oversampling.*

Response: Thanks for your suggestion. We agree that the use of SMOTE oversampling needs further verification. In the review process, our results suggested that the accuracies achieved using direct classifications for the random forest and DBN (Table S3) were better than those achieved using SMOTE or random oversampling. Therefore, all the information about SMOTE oversampling has been removed.

Comment (4): *The recent state-of-the-art method published in GigaScience ("Accurate prediction of personalized olfactory perception from large-scale chemoinformatic features.") was not discussed in this study. The author should compare with the previous method, or at least discuss the connections and differences between these studies.*

Response: We are grateful for your recommendation. We have studied the best algorithm for olfaction prediction in the DREAM challenge and further discussed the connections and differences between the findings (Page 10, Lines 215-232).

Comment (5): *The network architecture of deep belief network should be provided, including details such as number of layers, number of parameters.*

Response: Many thanks for your comment. We compared three DBN structures for the prediction of either color or odor, and optimizations of the parameters of each structure have been conducted. The architecture that performed best for both color and odor prediction was the input layer with 5270 neurons and only one RBM with 5270 visible neurons and 500 hidden neurons. The moderate performance was achieved with the input layer with 5270 neurons and two RBMs. One RBM was composed of 5270 visible neurons and 2000 hidden neurons, and the other contained 2000 visible neurons and 500 hidden neurons. The worst performance was achieved with the input layer with 5270 neurons and three RBMs. One RBM contained 5270 visible neurons and 2000 hidden neurons, one was composed of 2000 visible neurons and 1000 hidden neurons, and the last contained 1000 visible neurons and 500 hidden neurons. Therefore, the best architecture was used in the follow-up prediction. We have also added these details on Page 13, Lines 284-295.

Reviewer #2:

Comment (1): *The authors in their manuscript develop machine learning (random forest and DBN) trained models for distinguishing 12 distinct colours and 12 odours based on large-scale physicochemical features of 1267 and 598 structurally diverse molecules, respectively. In this analysis, the authors discuss identified important features for a specific classification. Moreover, shows some connections between colours, and odour features. The manuscript is well written, made it easier to go through the content. However, some major issues are listed below should discuss or clarify in the manuscript.*

Response: Thanks for your agreement on the merit and quality of our work. We also appreciate your constructive comments and have further discussed the major restrictions of our study (see the following responses).

Comment (2): *In the data description section: line 90 - 99: the decision of selecting these specific colours and odour is missing. For example where these colour for particular molecules previously defined from NCBI or they visually identify the colours of the molecules or they used some software for this identification. The similar question arises for the odours. I*

think odours are very subjective to the person who is labelling the features. This should be mentioned in the data description.

Response: Thanks so much for your constructive suggestion. We agree that olfactory perception varies greatly among individuals. So we selected molecules with definite color or odors as defined by the NCBI.

A previous study of personalized olfactory perception published in *GigaScience* used a dataset of molecules sensed by 49 voluntary people [1]. They found that the perceived attributes including the intensity were rated differently among individuals, which considerably complicated the prediction challenge. We further discussed the connections and differences between these studies and emphasized the data from NCBI as our "gold standard" in the revised manuscript (Page 10, Lines 215-221).

1. Hongyang Li, Bharat Panwar, Gilbert S. Omenn & Yuanfang Guan. Accurate prediction of personalized olfactory perception from large-scale chemoinformatic features. *GigaScience* 2017; 7, 1–11.

Comment (3): *line 111: replacing "NaN" with 0. I don't think the missing values should be treated this genitally. Unless all the missing values are because of one reason and the information is not needed for a particular molecule. The missing values in chemoinformatics dataset could be present because of various reasons, for example, the introduction of missing values is either no information was available (in literature/experiment etc) or due to the chemical calculation is not needed for this molecule. Both the cases can't have the same output. This should be reflected in your dataset and influence the model prediction. Also, mention how much missing data is present in your dataset.*

Response: Thanks for your suggestion. In our study, all the missing values are due to unavailable information in Dragon 7.0, which is the most used application for the calculation of molecular descriptors worldwide. The missing values simply mean that for the associated molecules, some descriptors have not been calculated for some reason, which commonly happens, as several descriptors have particular constraints (https://chm.kode-solutions.net/products_dragon_tutorial.php#01). In addition, our results of classification were quite good when substituting "NaN" with 0, indicating that these missing

values did not play significant roles in the prediction modeling. However, we agree that new information is required to confirm our findings if an upgraded version of the Dragon software becomes available. The reasons behind and statistics of missing data have been added in the data description according to your suggestion (Page 7, Lines 138-145).

Comment (4): *In Figure 1: it is unclear how odour dataset was included? Do you have two different workflows for colour and odour dataset?*

Response: Thanks for your suggestion. We have rearranged Figure 1 to integrate the workflows of color and odor prediction.

Comment (5): *I think the colour classification model is overestimating the prediction of the training dataset. For a clear understanding, you can report sensitivity, specificity, and F1 instead of accuracy, also because of accuracy paradox.*

Response: Many thanks for your comment. We are sorry that the sensitivity, specificity, and F1 which are regularly used for model evaluation in bi-classification were not fit for our study. Because both color and odor were divided into twelve categories, confusion matrixes were used to assist in observing the prediction effects of the random forest (Figure 2A, 3A), and column charts were used to assist in observing the prediction effects of the DBN (Figure 2B, 3B). To better evaluate our models of twelve-category classification, we added the kappa coefficient. Upon using all features to predict color, k = 1.0000 ± 0.0000 (mean ± SD) using the random forest, and k = 0.9400 ± 0.0030 (mean ± SD) using the DBN. Upon using all features to predict odor, k = 0.9232 ± 0.0037 (mean ± SD) using the random forest, and k = 0.9397 ± 0.0031 (mean ± SD) using the DBN. The kappa coefficients have been added in the results (Page7, Lines 152-153; Page8, Lines 177-178).

Comment (6): *Figure legend is missing, which makes it hard to read and understand the figures.*

Response: Thanks so much for your correction. All the figure legends were modified (Pages 21-22).

Comment (7): *From figures and text, it is unclear if the random forest performed better than DBN? This is not the main findings of this manuscript, however, it is helpful to identify which*

method performs better for future prediction. The impression of Figure 1 also suggests that there will be a comparison between the random forest and DBN. The comparison, in terms of evaluation measure (false positives, false negative, F1 measure), should be mentioned in the main publication.

Response: Thanks for your suggestion. We agree that the comparison between the random forest and DBN should be completed. To show our key results more clearly, we added boxplots presenting the results of the random forest and DBN for color and odor prediction (Figure 2C, D; Figure 3C, D). The new results for each fold in the 4-fold cross-validation are shown below. The table has also been added to the Supplementary Materials (Table S3).

Table S3. The results for each fold in the 4-fold cross-validation.

<table>
<tr><th>Task</th><th>Method</th><th>Number of features</th><th>Mean accuracy</th><th>Accuracy (4-fold cross-validation)</th></tr>
<tr><td rowspan="4">Color perception</td><td rowspan="2">Random forest</td><td>All features</td><td>100%</td><td>100%, 100%, 100%, 100%</td></tr>
<tr><td>24 features</td><td>99.45%</td><td>99.37%, 99.69%, 99.36%, 99.37%</td></tr>
<tr><td rowspan="2">DBN</td><td>All features</td><td>95.23%</td><td>95.89%, 94.93%, 94.88%, 95.23%</td></tr>
<tr><td>24 features</td><td>94.68%</td><td>95.24%, 95.33%, 94.46%, 93.67%</td></tr>
<tr><td rowspan="8">Odor perception</td><td rowspan="4">Random forest</td><td>All features</td><td>93.40%</td><td>93.33%, 93.92%, 93.20%, 93.15%</td></tr>
<tr><td>39 features</td><td>93.38%</td><td>93.33%, 93.90%, 93.18%, 93.11%</td></tr>
<tr><td>12 features</td><td>86.81%</td><td>86.00%, 86.49%, 87.07%, 87.67%</td></tr>
<tr><td>4 features</td><td>66.07%</td><td>68.67, 63.51%, 61.22%, 71.23%</td></tr>
<tr><td rowspan="4">DBN</td><td>All features</td><td>94.75%</td><td>95.24%, 94.35%, 94.27%, 95.13%</td></tr>
<tr><td>39 features</td><td>94.68%</td><td>95.24%, 95.33%, 94.46% , 93.67%</td></tr>
<tr><td>12 features</td><td>95.75%</td><td>95.53%, 95.67%, 96.03%, 95.78%</td></tr>
<tr><td>4 features</td><td>96.40%</td><td>95.67%, 96.73%, 96.46%, 96.75%</td></tr>
</table>

Overall, we found that the accuracy and kappa coefficient achieved using the random forest (100% ± 0.00%, 1.0000 ± 0.0000) were better than those achieved with the DBN (95.23% ± 0.40%, 0.9400 ± 0.0030) in color prediction with twelve categories. For odor prediction with twelve categories, the accuracy and kappa coefficient achieved using the DBN (94.75% ± 0.44%, 0.9397 ± 0.0031) were better than those achieved with the random forest (93.40% ± 0.31%, 0.9232 ± 0.0037). We further discussed the comparison in Page 10, Line 202-209. We are sorry that the sensitivity, specificity, and F1 which are regularly used for model evaluation in bi-classification, were not fit for our study.

Comment (8): *Line 218, could you elaborate on how random forest can effectively avoid overfitting and deliver generalized knowledge? there is no evidence suggesting that random forest avoids overfitting. For some reference check this blog: https://mljar.com/blog/random-forest-overfitting/.*

Response: Thanks for your scrupulous correction. We removed the statement "A random forest model can effectively avoid overfitting" to avoid potential controversies in the Method (Page 12, Lines 260-261).

Comment (9): *The random forest can produce variable importance, out of curiosity, are the variable importance comparable to the genetic algorithm? I think this is an interesting part of your publication that can be discussed.*

Response: Thanks for your suggestion. We agree that the comparison between the random forest and genetic algorithm could be very interesting. However, the dimensionality of the physicochemical data was very high with 5270 descriptors per molecule, and the data matrix was sparse in our study (Page 14, Lines 297-300). Many of the features are valued as "0" when calculated by the Dragon software, which means that they do not contribute to the classification (Page 14, Lines 304-306). Therefore, we preferred to combine the genetic algorithm and random forest algorithm, while the genetic algorithm was used for feature selection.

Comment (10): *Change heat map to heatmap in the publication*

Response: Thanks for your suggestion. We have changed the statement accordingly (Lines 164, 184).

Comment (11): *Use the full form first before abbreviation.*

Response: Thanks for your suggestion. We have made modifications to use the full form first. A list of abbreviations is presented on Page 16, Line 342-345.

Finally, thank you again for your acceptance and all of the helpful comments, and we hope that you will now find our revisions suitable for publication.

Sincerely yours,

Haotian Lin on behalf of all authors
