# Supplementary material for: Artificial intelligence deciphers codes for color and odor perceptions based on large-scale chemoinformatic data
Source: Gigascience. 2020 Feb 26;9(2):giaa011. doi: 10.1093/gigascience/giaa011 (PMC7043059; doi:10.1093/gigascience/giaa011)
Supplement: giaa011_GIGA-D-19-00112_Revision_2 [file giaa011_giga-d-19-00112_revision_2.pdf]

GigaScience

Artificial intelligence deciphers codes for color and odor perceptions based on large-scale chemoinformatic data

--Manuscript Draft--

**Manuscript Number:**	GIGA-D-19-00112R2
**Full Title:**	Artificial intelligence deciphers codes for color and odor perceptions based on large-scale chemoinformatic data
**Article Type:**	Research
**Funding Information:**	National Key Research and Development Program of China (2018YFC0116500) — Prof. Haotian Lin<br>National Natural Science Foundation of China (81770967) — Prof. Haotian Lin<br>National Natural Science Foundation of China (81822010) — Prof. Haotian Lin<br>the Key Research and Development Program of Guangdong Province (No. 2018B010109008) — Prof. Haotian Lin
**Abstract:**	Background<br>Color vision is the ability to detect, distinguish, and analyze the wavelength distributions of light independent of the total intensity. It mediates the interaction between an organism and its environment from multiple important aspects. However, the physicochemical basis of color coding has not been explored completely, and how color perception is integrated with other sensory input, typically odor, is unclear.<br>Results<br>Here, we developed an artificial intelligence platform to train algorithms for distinguishing color and odor based on the large-scale physicochemical features of 1267 and 598 structurally diverse molecules, respectively. The predictive accuracies achieved using the random forest and deep belief network for the prediction of color were 100.0% ± 0.0% and 95.23% ± 0.40% (mean ± SD), respectively. The predictive accuracies achieved using the random forest and deep belief network for the prediction of odor were 93.40% ± 0.31% and 94.75% ± 0.44% (mean ± SD), respectively. Twenty-four physicochemical features were sufficient for the accurate prediction of color, while thirty-nine physicochemical features were sufficient for the accurate prediction of odor. A positive correlation between the color coding and odor coding properties of the molecules was predicted. A group of descriptors was found to interlink prominently in color and odor perceptions.<br>Conclusions<br>Our random forest model and DBN accurately predicted the colors and odors of structurally diverse molecules. These findings extend our understanding of the molecular and structural basis of color vision and reveal the interrelationship between color and odor perceptions in nature.
**Corresponding Author:**	Haotian Lin, Ph.D., M.D.<br>Sun Yat-Sen University Zhongshan Ophthalmic Center<br>CHINA
**Corresponding Author Secondary Information:**	
**Corresponding Author's Institution:**	Sun Yat-Sen University Zhongshan Ophthalmic Center
**Corresponding Author's Secondary Institution:**	
**First Author:**	Xiayin Zhang
**First Author Secondary Information:**	
**Order of Authors:**	Xiayin Zhang

Powered by Editorial Manager® and ProduXion Manager® from Aries Systems Corporation

	Kai Zhang
	Duoru Lin
	Yi Zhu
	Chuan Chen
	Lin He
	Xusen Guo
	Kexin Chen
	Ruixin Wang
	Zhenzhen Liu
	Xiaohang Wu
	Erping Long
	Kai Huang
	Zhiqiang He
	Xiyang Liu
	Haotian Lin, Ph.D., M.D.
**Order of Authors Secondary Information:**	
**Response to Reviewers:**	Dear Scott Edmunds and Reviewers,<br><br>Thank you so much for the agreements and insightful suggestions on our manuscript.<br><br>The following are our point-by-point responses to the reviewers' comments and corresponding changes are marked in the revised manuscript. We hope that we have addressed all the suggestions adequately.<br><br>----------------------------------<br>Our point-by-point responses are as follows:<br><br>Reviewer #1:<br><br>Comment (1): Thank you for revising and improving the manuscript. The authors have addressed my previous concerns. I still have a few comments about the revised manuscript: In Figure 3A, it seems the model cannot distinguish "sweet" and "unpleasant" very well. But in general, "sweet" should be a "pleasant" odor instead of an "unpleasant" one. How can these results be reconciled?<br><br>Response: Thanks so much for your agreement on the merit of our work, as well as your constructive comments. We considered olfactory perception vary greatly among individuals, as your study discovered that the perceived attributes were rated differently among individuals, which considerably complicated the prediction challenge (GigaScience 2017; 7, 1–11). So in this study we emphasized the selection of molecules with definite color or odors were defined by NCBI (Line 103-104, 210-212). If the molecule is defined as "sweet" in NCBI, we try not to classify this "sweet" as a "pleasant sweet" or an "unpleasant sweet" according to our subjective feelings. And molecules with multiple odors that are difficult to define by NCBI were excluded.<br>We certainly agree that this process may reduce the credibility of results in the real world, and bring about the results that odor sensing was less accurate than that of color in our study (Discussion, Line 217-221). However, rather than arranging large-scale human resources from different races to distinguish odors "fairly", we prefer this way to deal with the data.<br><br>Comment (2): For Figure 2C-D and Figure 3C-D, it would be clearer if the median values of these boxplots are labeled.<br><br>Response: Thank you again for your acceptance and all of the helpful comments. We

have labeled the median values in the boxplots in Figure 2C-D and Figure 3C-D. The figure legends have been modified correspondingly (Line 457-465, 474-483).

Reviewer #2:

Comment (1): Figure 1: from the figure itself it looks like that the feature selection was done separately. However, to test the performance of the classifiers based on feature selected by the genetic algorithm random forest and DBN model were used again. This is unclear for this figure but clear in the text.

Response: Thanks so much for your constructive comments and suggestions for our study. We have rearranged Figure 1 to show the random forest and DBN models were used for a second time after feature selection. The figure legend has been modified correspondingly (Line 446-449).

Comment (2): Usually, a feature selection step is performed before model building and random forest can perform this selection on its own. I am not convinced with the response of the author that due to the sparsity of the matrix and huge physicochemical data they did not discuss the feature importance results from random forest however they consider random forest performs the best. This aspect should be discussed in the publication.

Response: We really appreciate your constructive suggestions. We have revised our method applying random forest models to perform feature selection on its own, and found that random forest alone did even better than combining it with the genetic algorithm using the same number of features (24 features for color perception, 39 features for odor perception). The results for each fold in the 4-fold cross-validation are shown below comparing two methods of feature selection after our correction, and the best performing results we used in the revised results are highlighted.
Table S3. The results for each fold in the 4-fold cross-validation.

Task	Method	Number of features	Mean accuracy	Accuracy (4-fold cross-validation)
Color perception	Random forest	① All features	100%	100%, 100%, 100%, 100%
		② 24 features #	100%	100%, 100%, 100%, 100%
		③ 24 features *	99.45%	99.37%, 99.69%, 99.36%, 99.37%
		② + ③features	100%	100%, 100%, 100%, 100%
	DBN	① All features	95.23%	95.89%, 94.93%, 94.88%, 95.23%
		② 24 features #	47.35%	47.95%, 49.06%, 46.50%, 45.89%
		③ 24 features *	44.20%	44.16%, 43.75%, 44.59%, 44.30%
		② + ③features	48.79%	53.63%, 44.69%, 50.00%, 46.84%
Odor perception	Random forest	① All features	93.40%	93.33%, 93.92%, 93.20%, 93.15%
		② 39 features #	93.40%	93.33%, 93.92%, 93.20%, 93.15%
		③ 39 features *	93.38%	93.33%, 93.90%, 93.18%, 93.11%
		② + ③features	93.40%	93.33%, 93.92%, 93.20%, 93.15%
	DBN	① All features	94.75%	95.24%, 94.35%, 94.27%, 95.13%
		② 39 features #	36.18%	43.33%, 30.41%, 36.73%, 34.25%
		③ 39 features *	31.46%	31.29%, 31.33%, 29.00%, 34.25%
		② + ③features	42.44%	48.00%, 38.51%, 48.30%, 34.93%

Random forest models
* The combination of random forest models and genetic algorithm

According to the new features selected by random forest models, we have made changes in the results section (Line 148-150, Line 166-168, Figure 2C-E, Figure 3C-E, Figure 4B and Table S1-S3). In the method section, we described the application of the two methods in detail (Line 280-299).

Comment (3): The results of the random forest are very impressive and confusing at the same time. I am unsure how can random forest achieve 100% accuracy with a sparse dataset as you mentioned in the comment for 12 different colours class. Because random forest -- bagging and suboptimal selection of splits may waste most

Powered by Editorial Manager® and ProduXion Manager® from Aries Systems Corporation

of the model insight on zero-only areas. It will be very informative if you could show AUC for each class individually.

Response: Thanks for your suggestion. The AUCs of the random forest in the prediction of twelve colors and twelve odors using all features are shown below. The figure has also been added to the Supplementary Materials (Figure S1).

Table S4. The prediction accuracies of random forest models for twelve colors and twelve odors using all features. AUC, area under the curve.

Task	Label	AUC	Task	Label	AUC
Color perception	White	0.9982	Odor perception	Ammonia	0.9985
	Yellow	0.9991		Aromatic	0.9985
	Colorless	0.9988		Characteristic	0.9957
	Red	0.9994		Flowery	0.9963
	Amber	0.9988		Fruity	0.9985
	Dark gray	1.0000		Mild	0.9939
	Brown	0.9983		Other	0.9982
	Orange	0.9988		Odorless	0.9960
	Purple	0.9968		Sweet	0.9654
	Blue	0.9988		Spicy	0.9952
	Green	0.9987		Pleasant	0.9987
	Black	0.9988		Unpleasant	0.9607

We confirmed the earlier result based on a re-examination of the source code (https://github.com/Hugo0512/ColorOdorprediction).

Comment (4): line 145: "The data was divided into the training and testing data sets without oversampling" - could mention the proportion of your data split into training and testing?

Response: Many thanks for your comment. We re-emphasized the classification method of training and testing data sets was k-fold cross-validations (k = 4) in this line (Line 131-132).

Comment (5): line 194-195: Pearson correlation coefficients > 0.1958, please write the exact coefficient value.

Response: Thanks so much for your scrupulous correction. The minimum absolute value of the Pearson correlation coefficients applied in this revised vision was 0.300552 (Line 180-181).

Comment (6): Figure 4: A) the intersecting part of the pie chart can be a different colour. Before Figure 4, there is no explanation of vital features. It will be useful to include the definition of the vital feature before or while writing the result on the correlation between colour and odour.

Response: Thanks so much for your scrupulous correction. The color in Figure 4A has been modified. We also unified our expression using “key physicochemical features” (Line 92, 94, 148, 167, 195, 488, Figure 4B), and defined it as the physicochemical descriptors contributed most to the predictive accuracies (Line 90-93, 301-303).

Comment (7): Line 209-210: It will be helpful for the readers if the authors discuss the rationale behind the connected colour and odour physicochemical features.

Response: Thanks for your suggestion. The shared and prominently interlinked key physicochemical features identified in predicting color and odor have not been reported yet. We cautiously discussed the significance in Page 10, Line 199-202.

Comment (8): Line 215: it would be interesting to show the evaluation scores from the DREAM challenge participants to directly compare the results from your claim.

Response: Thanks for your suggestion. We agree that the evaluation scores from the DREAM challenge participants should be mentioned in the discussion (Line 203-205). Actually, there were great differences in data types and evaluation criteria between our study and the DREAM challenge. We solved one question with a multi category for

	each time here, while multiple questions are classified together in the DREAM challenge. The evaluation scores we used were accuracies, kappa coefficients and AUCs, the evaluation in DREAM challenge was based on the Pearson's correlation between observed and predicted perceptions.<br><br>Comment (9): line 221, is a redundant sentence instead you can write - "The winning algorithm of the DREAM challenge indicated that the random forest outperforms other base learning methods. "<br><br>Response: Thanks so much for your scrupulous correction. We removed the statement accordingly in Line 208-210.<br><br>Comment (10): line 228 no comma needed.<br><br>Response: Thanks so much for your scrupulous correction. The sentence has been removed in the revised version.<br><br>Comment (11): line 461: and is missing in this line - "all features and the top 24 features".<br><br>Response: Thanks so much for your scrupulous correction. We have modified the figure legends (Line 457-465).<br><br>Comment (12): line 202, as a reader it would be informative to get the definition of terse framework or at least a citation?<br><br>Response: Thanks so much for your scrupulous correction. We removed the word "terse" to avoid misunderstanding (Line 188).<br><br>Comment (13): The cutoff to decide the number of features is not clear in the manuscript.<br><br>Response: Many thanks for your comment. In order to compare random forest algorithm itself with the combination of the genetic algorithm and random forest algorithm, the numbers of the key features selected were similar. As random forest model could not choose the cut off by itself, the cutoff to decide the number of features was determined by the genetic feature selection task. After running the genetic feature selection task 20 times, 24 descriptors were selected 18 times with a classification accuracy of 100% ± 0.0% in the random forest model for color, 39 descriptors were selected 16 times with a classification accuracy of 93.38% ± 0.31% in the random forest model for odor. (Method, Line 287-291, 296-299).<br><br>--<br>Finally, thank you again for your acceptance and all of the helpful comments, and we hope that you will now find our revisions suitable for publication.<br><br>Sincerely yours,<br>Haotian Lin on behalf of all authors
**Additional Information:**	
**Question**	**Response**
Are you submitting this manuscript to a special series or article collection?	No
**Experimental design and statistics**<br><br><br>Full details of the experimental design and statistical methods used should be given in the Methods section, as detailed in our Minimum Standards Reporting Checklist. Information essential to interpreting the	Yes

data presented should be made available in the figure legends.<br><br>Have you included all the information requested in your manuscript?	
**Resources**<br><br>A description of all resources used, including antibodies, cell lines, animals and software tools, with enough information to allow them to be uniquely identified, should be included in the Methods section. Authors are strongly encouraged to cite Research Resource Identifiers (RRIDs) for antibodies, model organisms and tools, where possible.<br><br>Have you included the information requested as detailed in our Minimum Standards Reporting Checklist?	Yes
**Availability of data and materials**<br><br>All datasets and code on which the conclusions of the paper rely must be either included in your submission or deposited in publicly available repositories (where available and ethically appropriate), referencing such data using a unique identifier in the references and in the “Availability of Data and Materials” section of your manuscript.<br><br>Have you have met the above requirement as detailed in our Minimum Standards Reporting Checklist?	Yes

Manuscript
Click here to access/download;Manuscript;Manuscript (with changes tracked).docx
Click here to view linked References

Artificial intelligence deciphers codes for color and odor perceptions based on large-scale chemoinformatic data

Xiayin Zhang[1†] (zhangxiayin@gzzoc.com), Kai Zhang[1,2†] (hugo88315@163.com), Duoru Lin[1†] (linduoru@sina.com), Yi Zhu[1,3] (y.zhu17@med.miami.edu), Chuan Chen[1,4] (c.chen30@med.miami.edu), Lin He[2] (August_us@163.com), Xusen Guo[5] (guoxs3@mail2.sysu.edu.cn), Kexin Chen[1] (873490288@qq.com), Ruixin Wang[1] (ruiruiw413@aliyun.com), Zhenzhen Liu[1] (liu_zhenzhen@qq.com), Xiaohang Wu[1] (1034281949@qq.com), Erping Long[1] (longerping@qq.com), Kai Huang[5] (huangk36@mail.sysu.edu.cn), Zhiqiang He[6] (hezq@bupt.edu.cn), Xiyang Liu[2] (xyliu@xidian.edu.cn) and Haotian Lin[1,7*] (haot.lin@hotmail.com).

[1]State Key Laboratory of Ophthalmology, Zhongshan Ophthalmic Center, Sun Yat-sen University, Guangzhou 510060, China;

[2]School of Computer Science and Technology, Xidian University, Xi'an 710000, China;

[3]Department of Molecular and Cellular Pharmacology, University of Miami Miller School of Medicine, Miami, Florida 33136, USA;

[4]Sylvester Comprehensive Cancer Center, University of Miami Miller School of Medicine, Miami, Florida 33136, USA;

[5]Key Laboratory of Machine Intelligence and Advanced Computing, Ministry of Education School of Data and Computer Science, Sun Yat-Sen University;

[6]Key Laboratory of Universal Wireless Communications, Beijing University of Posts and Telecommunications, Beijing 100876, China.

[7]Center of Precision Medicine, Sun Yat-sen University, Guangzhou 510080, China.

[†] These authors contributed equally to this work.

* **Corresponding Author:**

Prof. Haotian Lin

Xian Lie South Road 54#, Guangzhou, China, 510060

Telephone: +86-13802793086

Email address: haot.lin@hotmail.com

Abstract

Background

Color vision is the ability to detect, distinguish, and analyze the wavelength distributions of light independent of the total intensity. It mediates the interaction between an organism and its environment from multiple important aspects. However, the physicochemical basis of color coding has not been explored completely, and how color perception is integrated with other sensory input, typically odor, is unclear.

Results

Here, we developed an artificial intelligence platform to train algorithms for distinguishing color and odor based on the large-scale physicochemical features of 1267 and 598 structurally diverse molecules, respectively. The predictive accuracies achieved using the random forest and deep belief network for the prediction of color were 100.0% ± 0.0% and 95.23% ± 0.40% (mean ± SD), respectively. The predictive accuracies achieved using the random forest and deep belief network for the prediction of odor were 93.40% ± 0.31% and 94.75% ± 0.44% (mean ± SD), respectively. Twenty-four physicochemical features were sufficient for the accurate prediction of color, while thirty-nine physicochemical features were sufficient for the accurate prediction of odor. A positive correlation between the color coding and odor coding properties of the molecules was predicted. A group of descriptors was found to interlink prominently in color and odor perceptions.

Conclusions

Our random forest model and DBN accurately predicted the colors and odors of

structurally diverse molecules. These findings extend our understanding of the molecular and structural basis of color vision and reveal the interrelationship between color and odor perceptions in nature.

Keywords: color perception; odor perception; random forest; deep belief network; physicochemical features.

Background

Color vision mediates the relationship between an organism and its environment in multiple important ways, including influencing mate choice, camouflage, and speciation [1]. We see a colorful world because different objects are composed of materials with different reflectance spectra in the wavelength range visible to our eyes [2]. Although knowledge of fundamental optical processes such as reflection, refraction, interference, diffraction, and scattering is accumulating [3], we lack the ability to recognize the color of cellular structure and pattern formation at optical scales from nanometers to microns.

Nature creates various colorful materials based on physicochemical properties including topological and geometrical properties that humans cannot easily see [4, 5]. For instance, the color changes from bright yellow through reddish–purple to blue when the size of a gold sample is decreased [6]. The different colors of disubstituted benzenes were discovered to be related to differences in the molecular structure with ortho, meta and para substitutions [7, 8]. The odors of chemicals are also fully encoded within their specific physicochemical properties [9, 10]. The compositions and structures of functional groups have been suggested to be crucial for the perception of aroma [11]. Moreover, evidence of the interaction between color vision and olfaction has been discovered [12]. For example, the odor of a host plant can modify the color sensed by a swallowtail butterfly [13]. The odor of wine can be predicted according to its color [14]. Additionally, the perceived intensity of an odor is positively correlated with the intensity of color [15, 16]. Neuroimaging and

repetitive transcranial magnetic stimulation studies showed that high-level odor processing also actives the visual cortex [17, 18]. However, the relationship between color and odor in terms of molecular physicochemical properties is largely unknown.

Artificial intelligence (AI) tools can be optimized to infer the innate laws of natural processes through machine learning tasks based on large-scale data sets and make predictions of the unknown [19, 20]. In the chemical sciences, AI has been used to guide chemical and material design, synthesis, characterization, and modeling [21, 22]. Previous researchers have equipped AI with a “nose” to predict human olfactory perception from the physicochemical features of 476 molecules and 21 perceptual attributes perceived by 49 individuals [23].

Here, we developed a random forest model and deep belief network (DBN) to predict the colors and odors of chemicals based on their molecular descriptors. We applied random forest and the combination of the genetic algorithm and random forest algorithm for feature selection to identify the key physicochemical features that contribute most to the predictive accuracies. In addition, we investigated the connection between the key physicochemical features in color and odor coding to unravel the commonality between visual and olfactory perception.

Data Description

Data collection and labeling. A total of 1267 structurally diverse molecules was used for color prediction in this study, and 598 structurally diverse molecules were used for odor prediction. The color, odor and three-dimensional (3D) structure data of these

molecules were all collected from the key chemical information resource at the U.S. National Center for Biotechnology Information, PubChem [24] (https://pubchem.ncbi.nlm.nih.gov) between June 1, 2017, and November 30, 2017. Molecules with definite colors or odors were defined from PubChem, and molecules with multiple colors or odors that are difficult to define were excluded. The data set of colors was classified into 12 diverse colors, including yellow (257 molecules), white (301 molecules), orange (31 molecules), red (16 molecules), purple (11 molecules), green (24 molecules), blue (9 molecules), brown (20 molecules), amber (15 molecules), gray (6 molecules), black (17 molecules) and colorless (560 molecules). The data set of odors was classified into 12 diverse odors, including ammonia (37 molecules), aromatic (36 molecules), characteristic (27 molecules), flower (19 molecules), fruity (29 molecules), mild (38 molecules), other (127 molecules), pleasant (16 molecules), unpleasant (23 molecules), spicy (54 molecules), sweet (30 molecules) and odorless (162 molecules).

Physicochemical features of the molecules. The PubChem compound identifier for each molecule was provided (Supplementary data). We applied the commercial chemoinformatics software package Dragon (version 7.0, https://chm.kode-solutions.net/products_dragon_papers.php) to calculate 5270 physicochemical descriptors for each of the molecules, including the simplest atom types, functional groups and fragment counts, topological and geometrical descriptors, 3D descriptors, several property estimations (such as log*P*) and drug-like and lead-like alerts (such as the Lipinski's alert). These molecular descriptors are formal

mathematical representations of a molecule and include their definition, symbols and labels, formulas, some numerical examples, data, and molecular graphs, as presented in the Handbook of Molecular Descriptors [25]. The missing values marked as “NaN” simply mean that for these molecules, some descriptors have not been calculated for some reason, which is common because several descriptors have particular constraints. Molecules with more than 2000 descriptors marked as “NaN” were not used. We replaced all of the “NaN” entries with “0” during the dataset preprocessing. For molecules with color, the average number of “NaN” within 5270 descriptors was 353 per molecule. For molecules with odor, the average number of “NaN” within 5270 descriptors was 28 per molecule. The data was divided into the training and testing data sets without oversampling using k-fold cross-validations (k = 4). The overall workflow is shown in Figure 1.

Results

Color prediction

Random forest and DBN algorithms were applied for the *in silico* test. Using k-fold cross-validations (k = 4), the random forest model identified and utilized the most discriminative features with 100.00% ± 0.0% (mean ± SD) accuracy in the prediction of twelve colors (Figure 2A, C, Figure S1), with a kappa coefficient of 1.0000 ± 0.0000 (mean ± SD). As a type of probability generation model consisting of multiple restricted Boltzmann machines (RBMs), the DBN also performed excellently, with a predictive accuracy of 95.23% ± 0.40% (mean ± SD) (Figure 2B, D) and a kappa coefficient of 0.9400 ± 0.0030 (mean ± SD).

Key physicochemical features for color perception

The random forest algorithm and the combination of the genetic algorithm and random forest algorithm both enable us to estimate the importance of each molecular descriptor by permuting the values of the descriptors across samples and computing the increases in prediction errors. Twenty-four descriptors were selected as the key physicochemical features in random forest algorithm with a classification accuracy of 100.00% ± 0.0% by using *k*-fold cross-validations (*k* = 4). The molecular descriptor "B05[F-X]" ranked first, followed by "SddsAs", "RDF155s" and "F08[O-Si]". The heatmap of the hierarchical cluster analysis between the twenty-four key features and the twelve colors is shown in Figure 2E. "B10[P-X]", "B05[P-P]", "B05[P-Cl]", "HVcpx", and "ATS5i" were the main contributors to white, whereas "CATS3D_00_DL" and "E1e" were the most important features in predicting yellow. Information relevant to the key physicochemical features for color perception is reported in Table S1.

Distinction and connection with olfaction perception

We next applied the AI platform to predict odor perception based on physicochemical features. In total, 598 structurally diverse molecules were collected and classified into twelve diverse odors based on PubChem [24], including pleasant, unpleasant, ammonia, aromatic, flowery, fruity, spicy, sweet, mild, odorless, characteristic, and other. The accuracies of the odor prediction were 93.40% ± 0.31% for the random forest model using *k*-fold cross-validations (*k* = 4) (Figure 3A, C, Figure S1) and 94.75% ± 0.44% for the DBN (Figure 3B, D), with kappa coefficients of 0.9232 ±

0.0037 and 0.9397 ± 0.0031, respectively. Thirty-nine descriptors were selected as the key physicochemical features in the random forest model with a classification accuracy of 93.40% ± 0.31% (Table S3). The heatmap of the hierarchical cluster analysis between the thirty-nine key physicochemical features and the twelve odors is shown in Figure 3E. Information relevant to the key physicochemical features for odor perception is presented in Table S2.

To understand the correlation between color and odor, we collected 90 molecules with both color and odor information and analyzed the two groups using a chi-square test. The colors were divided into two categories (white, colorless/other), as were the odors (odorless/other). A correlation was predicted for both types of perception for these molecules (χ^2 = 17.445; P < 0.001). In the complex network of color and odor, key physicochemical features for color and odor prediction were converted into z-scores, and the relationship between each pair of attributes was evaluated by the Pearson correlation coefficient. More than fifty molecular descriptors were found to be interlinked prominently according to their correlation values (the absolute value of the Pearson correlation coefficients ≥ 0.300552) (Figure 4). Three key features "B05[P-CI]", "F08[O-B]" and "CATS3D_14_NL" were shared for both color perception and odor perception.

Discussion

Clarifying the underlying mechanism of color vision is inherently challenging, as the cognitive process of color vision is multidimensional and includes crossover among

the morphology and function of the human visual system [26-28]. Here, we established a framework for distinguishing color without wavelengths based on only 24 physicochemical features. We found that the accuracy and kappa coefficient achieved using random forest (100% ± 0.00%, 1.0000 ± 0.0000) were better than those achieved with the DBN (95.23% ± 0.40%, 0.9400 ± 0.0030) in color prediction with twelve categories. For odor prediction with twelve categories, the accuracy and kappa coefficient achieved using the DBN (94.75% ± 0.44%, 0.9397 ± 0.0031) were better than those achieved with the random forest (93.40% ± 0.31%, 0.9232 ± 0.0037). Our findings also suggested that key physicochemical features in distinguishing color and odor are connected. The 2D Atom Pairs descriptors and many other descriptors interlink at the network between color and odor perception, indicating that both color and odor perceptions are partially determined by the physicochemical properties of the molecules and that color and odor perceptions are closely interrelated. With the shared and prominently interlinked key physicochemical features identified in predicting color and odor, our results tend to explain the reason why modifying color property of an object changes the odor property of the same object [14].

Previous studies on predicting odor have been conducted by the DREAM Olfaction Prediction Challenge [23, 29], with the best Pearson's correlation coefficient achieved around 0.3 between observed and predicted perceptions. A dataset of 476 molecules sensed by 49 voluntary people was applied, and the perceived attributes including the intensity were found to rate differently among the individuals, which considerably complicated the prediction challenge [29]. The winning algorithm of the DREAM

challenge indicated that the random forest outperforms other base learners (linear, ridge, and support vector machine) in predicting odor [29]. Our study collected a total of 598 structurally diverse molecules and classified them into twelve diverse odors based on PubChem to avoid a subjective effect on odor perception. We added the DBN method and achieved the best result in odor prediction with a classification accuracy of 94.75% ± 0.44% for 12 categories. In contrast, the random forest showed a higher accuracy in color prediction than DBN did. Above all, we believe that the machine learning method can be extended to predict both physicochemical properties.

In addition, odor sensing was found to be less accurate than that of color. Several factors may affect the accuracy of the AI in odor perception. First, odor perception is more subjective based on perceived biases, and it is challenging to confirm the number and character of its perceptual dimensions [30]. Defining a specific odor is especially difficult for human beings compared with other sensory modalities [31]. Second, the olfactory system involves high-dimensional input with attached arbitrary associations, whereas color vision occurs under predefined spatial conditions [12]. Thus, the processing demands of the two systems are not entirely consistent with each other. Third, the two systems employ different strategies in temporal coding to convey information. The olfactory system uses temporal coding to increase its representational capacity, while the visual system uses temporal coding to reduce the redundancy [12].

In this study, we add new insight into the decoding of color vision, but the controlling and tuning of these codes require further investigation. Inspired by the key

physicochemical features involved in color prediction, researchers may be able to develop materials with vivid colors for potential applications in sensing technologies, security, light-emitting sources, and paints [32-34].

Potential implications

The ability to explain visual neural activities from the perspective of AI would also enable us to build an artificial vision system that could favorably stimulate the color vision of an individual. Once the perception process of human color vision is completely decoded, the AI platform may help in the design of artificial brain stimulation interfaces that can restore color vision and enable blind patients to “see” colors without biological eyes.

Methods

Random forest algorithm

Random forest is an ensemble learning method for regression and classification [35]. In a random forest model, each decision tree is built from a random sampling of samples and features, which can deliver generalized knowledge [35]. Furthermore, a random set of features is used to determine the best split at each node during the construction of a tree. Here, the dimensionality of the physicochemical data was high, with 5270 descriptors per molecule, and the perception data matrix was sparse. By averaging hundreds of trees in this work, the effects of outliers and noise were reduced. The random forest parameter mTry (i.e., the number of input variables randomly chosen at each split) was set to 72 (square root of 5270 features), while the

other random forest parameter nTree (i.e., the number of trees to grow for each forest) was set to 100. k-fold cross-validation (k = 4) was applied for the classification.

Deep belief network (DBN)

DBN is a type of probability generative model that consists of multiple RBMs. The superposition of multiple RBMs solves the training problem of multiple layered neural networks. The overall training process of the DBN includes two stages: a pretraining stage and a fine-tuning stage [36]. 1) Pretraining stage: Each RBM includes a visual layer and a hidden layer. There are no interlayer connections between the visual layer and hidden layer. After training the first RBM, the activation value of the hidden layer of the first RBM is input into the visual layer of the second RBM. 2) Fine-tuning stage: With the help of the BP neural network that resides after the last RBM and the chain rule of derivation, the DBN will be trained as a whole neural network. In this study, the input of the DBN is the vector consisting of 5270 molecular descriptors. During the first stage of the DBN, the dimensions of the vector are compressed. During the second stage, the compressed vector can be used for classification.

We compared three DBN structures for the prediction of either color or odor, and optimizations of the parameters of each structure were conducted. The architecture that performed best in both color and odor prediction was the input layer with 5270 neurons and only one RBM with 5270 visible neurons and 500 hidden neurons. The moderate performance was achieved with the input layer with 5270 neurons and two RBMs. One RBM was composed of 5270 visible neurons and 2000 hidden neurons,

and the other contained 2000 visible neurons and 500 hidden neurons. The worst performance was achieved with the input layer with 5270 neurons and three RBMs. One RBM contained 5270 visible neurons and 2000 hidden neurons, one was composed of 2000 visible neurons and 1000 hidden neurons, and the last contained 1000 visible neurons and 500 hidden neurons. Therefore, the best architecture was used in the follow-up prediction.

Feature selection

Random forest algorithm and the combination of the genetic algorithm [37,38] and random forest algorithm were both applied to select the key features in this study. For random forest algorithm, the samples left-out in the training of each classifier (referred to as out-of-bag samples) are used for feature selection by determining the importance of different features during classification process. A value of "0" signifies that the feature corresponding to this bit is not needed for the classification; otherwise, the feature is needed for the classification. A total of 1601 features were recognized as needed for the classification of color, and 1820 were recognized as needed for the classification of odor in random forest algorithm. In order to compare with the combination of the genetic algorithm and random forest algorithm, the numbers of the key features selected were similar.

Genetic algorithms designed for feature selection can implement feature selection and classification processes simultaneously [39]. The accuracy of the random forest was adopted as the fitness evaluation function of the genetic algorithm. The chromosome coding method was binary coding, and the length of the chromosome was equal to the

dimension of the feature vector. Because of the randomness of the genetic algorithm, the experiment was conducted 20 times. After running the genetic feature selection task 20 times, 24 descriptors were selected 18 times for color, 39 descriptors were selected 16 times for odor.

Feature ranking

Feature ranking for random forest algorithm used out of bag permutation error. With the features selected from the genetic algorithm, feature ranking was performed to study which attributes were more important for classification. In this process, for a feature A_i in the feature set $\{A_1, A_2 \ldots A_n\}$, the validating accuracy for the original validation dataset is *acc1*. The validatiom accuracy obtained with the random permutation of A_i is *acc2*. |*acc2-acc1*| is an indicator used to measure the importance of A_i. Then, all features are compared with this indicator. Because of the randomness of the random forest, this process was conducted 20 times.

Hierarchical clustering

Hierarchical approaches have the ability to simultaneously uncover multiple layers of a clustering structure [40]. The R heatmap package was used for clustering in this study.

Statistical analysis

The data were collected using the Qualtrics Web-based questionnaire package and analyzed using IBM SPSS Statistics version 24.

Availability of Supporting Data and Materials

All methods were implemented with MATLAB R2016a on HP Z420 workstation with Intel Xeon CPU E5-1620 v2@ 3.70GHZ and 16GB RAM. The operating system is Windows 7. Data corresponding to the molecules used in this study are presented in Supplementary Data1-3. The source code of this study is presented in https://github.com/Hugo0512/ColorOdorprediction.

Additional Files

Table S1. Attribute importance ranking of color.

Table S2. Attribute importance ranking of odor.

Table S3. The results for each fold in the 4-fold cross-validation.

Figure S1. The prediction accuracies of random forest models for twelve colors and twelve odors using all features.

Supplementary Data1. The datasets of the 1267 structurally diverse molecules labeled with 12 diverse colors and 5270 molecular descriptors.

Supplementary Data2. The datasets of the 598 structurally diverse molecules labeled with 12 diverse odors and 5270 molecular descriptors.

Supplementary Data3. The datasets of the 90 molecules with both color and odor information.

Abbreviations

3D, Three dimensional; AI, Artificial intelligence; DBN, Deep belief network; Dragon, Software for the calculation of molecular descriptors; GETAWAY, Geometry, topology and atom-weights assembly; RBM, Restricted Boltzmann machine.

Completing interests

The authors declare that they have no competing interests.

Funding

This study was funded by the National Key R&D Program of China (2018YFC0116500), the Key Research and Development Program of Guangdong Province (No. 2018B010109008), the National Natural Science Foundation of China (81770967, 81822010). The funders had no role in the study design, data collection, and analysis, the decision to publish or the preparation of the manuscript.

Author contributions

H.T.L., X.Y.Z. and D.R.L. conceived and designed the prediction algorithm, X.Y.Z., K.Z., D.R.L. and L.H. were responsible for data management and performing the computational analyses. R.X.W., Z.Z.L., X.H.W., and E.P.L. analyzed the discriminative features and prepared the figures. H.T.L., X.Y.Z. and D.R.L. contributed to the writing of the manuscript. Z.Y., C.C., X.S.G., K.X.C., K.H., X.Y.L., and Z.Q.H. contributed to the critical review of the study, and all authors read and approved the final manuscript.

Acknowledgments

We thank Xiaoming Chen (School Of Chemistry, Sun Yat-sen University) for reading, discussing and providing constructive comments for the manuscript.

References

1. Pete Vukusic & J. Roy Sambles. Photonic structures in biology. *Nature* 2003; 424, 852–855.

2. Le Chang, Pinglei Bao & Doris Y. Tsao. The representation of colored objects in

macaque color patches. *Nature Communications* 2017; 8 (1).

3. S Kinoshita, S Yoshioka & J Miyazaki. Physics of structural colors. *Rep. Prog. Phys*; 2008, 71, 30pp.

4. Wilkinson, F.A. & Murillo, S.G. Advanced inorganic chemistry. 1988. Wiley.

5. McMurry, John. Organic chemistry. 2007. Brooks Cole.

6. Hallenbeck. Recent Advances in QSAR Studies. *Challenges & Advances in Computational Chemistry & Physics* 2010; 8, 31-32.

7. Paul, A. The use of nanocrystals in biological detection. *Nat Biotechnol* 2004; 22, 47-52.

8. Chen, F. & Gerion, D. Fluorescent CdSe/ZnS Nanocrystal−Peptide Conjugates for Long-term, Nontoxic Imaging and Nuclear Targeting in Living Cells. *Office of Scientific & Technical Information Technical Reports* 2004; 4, 1827-1832.

9. Rossiter, K.J. Structure−Odor Relationships. *Chemical ReviewsChemical ReviewsChem. Rev.* 1996; 96, 3201-3240.

10. Turin, L. A method for the calculation of odor character from molecular structure. *J Theor Biol* 2002; 216, 367-385.

11. Czerny, M., Brueckner, R., Kirchhoff, E., Schmitt, R. & Buettner, A. The influence of molecular structure on odor qualities and odor detection thresholds of volatile alkylated phenols. *Chem Senses* 2011; 36, 539.

12. Gire, D.H., *et al*. Temporal processing in the olfactory system: can we see a smell. *Neuron* 2013; 78, 416-432.

13. Yoshida, M., Itoh, Y., Ômura, H., Arikawa, K. & Kinoshita, M. Plant scents modify innate color preference in foraging swallowtail butterflies. *Biol Lett* 2015; 11.

14. Morrot, G., Brochet, F. & Dubourdieu, D. The Color of Odors. *Brain & Language* 2001; 79, 309-320.

15. Zellner, D.A. & Kautz, M.A. Color affects perceived odor intensity. *J Exp Psychol Hum Percept Perform* 1990; 16, 391-397.

16. Dubose, C.N., Cardello, A.V. & Maller, O. Effects of colorants and flavorants on identification, perceived flavor and hedonic quality of fruit-flavored beverages and cake. *J Food Sci* 2010; 45, 1393-1399.

17. Royet, J.P., *et al.* Functional anatomy of perceptual and semantic processing for odors. *J Cogn Neurosci* 1999; 11, 94-109.

18. Jadauji, J.B., *et al.* Modulation of olfactory perception by visual cortex stimulation. *Journal of Neuroscience the Official Journal of the Society for Neuroscience* 2012; 32, 3095.

19. Gershman, S.J., Horvitz, E.J. & Tenenbaum, J.B. Computational rationality: A converging paradigm for intelligence in brains, minds, and machines. *Science* 2015; 349, 273-278.

20. Sanchez-Lengeling, B. & Aspuru-Guzik, A. Inverse molecular design using machine learning: Generative models for matter engineering. *Science* 2018; 361, 360-365.

21. Butler, K.T., Davies, D.W., Cartwright, H., Isayev, O. & Walsh, A. Machine learning for molecular and materials science. *Nature* 2018; 559, 547-555.

22. Paruzzo, F.M., *et al.* Chemical shifts in molecular solids by machine learning. *Nat Commun* 2018; 9, 4501.

23. Keller, A., *et al.* Predicting human olfactory perception from chemical features of odor molecules. *Science* 2017; 355, 820-826.

24. Kim, S., *et al.* PubChem Substance and Compound databases. *Nucleic Acids Res* 2016; 44, D1202-1213.

25. Todeschini, R. & Consonni, V. Handbook of Molecular Descriptors. 2000.

26. Solomon, S.G. & Lennie, P. The machinery of colour vision. *Nat Rev Neurosci* 2007; 8, 276-286.

27. Bennett, A. .D. & Théry, M. Avian Color Vision and Coloration: Multidisciplinary Evolutionary Biology. *Am Nat* 2007; 169, S1-1S6.

28. Kelber, A. & Osorio, D. From spectral information to animal colour vision: experiments and concepts. *Proceedings: Biological Sciences* 2010; 277, 1617-1625.

29. Hongyang Li, Bharat Panwar, Gilbert S. Omenn & Yuanfang Guan. Accurate prediction of personalized olfactory perception from large-scale chemoinformatic features. *GigaScience* 2017; 7, 1–11.

30. Kaeppler, K. & Mueller, F. Odor classification: a review of factors influencing perception-based odor arrangements. *Chem Senses* 2013; 38, 189-209.

31. Wippich, W., Mecklenbräuker, S. & Trouet, J. Implicit and explicit memories of odors. *Archiv Für Psychologie* 1989; 141, 195.

32. Hwang, J., *et al.* Electro-tunable optical diode based on photonic bandgap liquid-crystal heterojunctions. *Nat Mater* 2005; 4, 383-387.

33. Lee, H.S., Shim, T.S., Hwang, H., Yang, S.M. & Kim, S.H. Colloidal Photonic Crystals toward Structural Color Palettes for Security Materials. *Chemistry of Materials* 2013; 25, 2684-2690.

34. Sung Yeun, C., *et al.* Mesoporous bragg stack color tunable sensors. *Nano Lett* 2006; 6,

2456-2461.

35. Breiman, L. Random Forests. *Mach Learn* 2001; 45, 5-32.

36. Le, R.N. & Bengio, Y. Representational power of restricted boltzmann machines and deep belief networks. *Neural Comput* 2008; 20, 1631-1649.

37. Wang L, et al. Comparative analysis of image classification methods for automatic diagnosis of ophthalmic images. *Scientific reports* 2017; 7, 41545.

38. Zhang K, et al. Systemically modeling the relationship between climate change and wheat aphid abundance. *Science of The Total Environment* 2019; 674, 392-400.

39. Zhang K, et al. Prediction of postoperative complications of pediatric cataract patients using data mining. *J Transl Med* 2019; 3,17(1).

40. Eisen, M.B., *et al.* Cluster analysis and display of genome-wide expression patterns. *Proceedings of the National Academy of Sciences* 1998; 14863-14868.

Figure legends

Figure 1. The overall workflow of color prediction and odor prediction. 1267 structurally diverse molecules were labeled with 12 diverse colors, and 598 structurally diverse molecules were labeled with 12 diverse odors. In addition, 5270 physicochemical features of each molecule were generated by Dragon. Random forest models and deep belief networks were built to predict colors or odors using their physicochemical features. Feature selection were conducted by random forest models and the combination of random forest and the genetic algorithm. With the selected feature, random forest models and deep belief networks were reused for color and odor prediction. The models were evaluated based on the means and variances of the accuracies between the labeled and predicted colors or odors.

Figure 2. Color prediction using the random forest model and DBN. A. The confusion matrix for the classification of color with 100.00% accuracy by the random forest. The X-axis presents the labeled colors of the molecules, and the Y-axis presents the predicted colors of the molecules. B. The classification results for color were as high as 95.23% using the DBN. The *X*-axis presents the learning rate, the *Y*-axis presents the algorithm parameter “momentum”, and the *Z*-axis presents the accuracy rate. C. The boxplot presenting the accuracy of color prediction using the random forest with all features, the top 24 features selected by random forest models, the top 24 features selected by the combination of random forest and the genetic algorithm and the total 48 features from above. The median values of these boxplots are labeled. D. The boxplot presenting the accuracy of color prediction using the DBN

with all features, the top 24 features selected by random forest models, the top 24 features selected by the combination of random forest and the genetic algorithm and the total 48 features from above.The median values of these boxplots are labeled. [#] Random forest models; [*] The combination of random forest models and genetic algorithm. E. The heatmap of the the correlation values between the top 24 features selected by random forest models and the twelve colors based on the hierarchical clustering framework. The connections between the colors and descriptors were calculated by the Euclid distances.

Figure 3. Odor prediction using the random forest model and DBN. A. The confusion matrix for the classification of odor with 93.40% accuracy by the random forest. B. The classification results for odor were as high as 94.75% using the DBN. The *X*-axis presents the learning rate, the *Y*-axis presents the algorithm parameter “momentum”, and the *Z*-axis presents the accuracy rate. C. The boxplot to present the accuracy of color prediction using the random forest with all features, the top 39 features selected by random forest models, the top 39 features selected by the combination of random forest and the genetic algorithm and the total 78 features from above. The median values of these boxplots are labeled. D. The boxplot presenting the accuracy of color prediction using the DBN with all features, the top 39 features selected by random forest models, the top 39 features selected by the combination of random forest and the genetic algorithm and the total 78 features from above. The median values of these boxplots are labeled. [#] Random forest models; [*] The combination of random forest models and genetic algorithm. E. The heatmap of the correlation values

between the top 39 features selected by random forest models and the twelve odors based on the hierarchical clustering framework. Connections between the odors and descriptors were calculated by the Euclid distances.

Figure 4. The correlations between color and olfaction perception. A. Of the 1267 molecules with color, 90 also had odor information. B. Schematic diagram of the key physicochemical features for color and odor perceptions in the interactome. The key features for color perception were closely connected with the key features for odor perception. The distance of each line represents its correlation value.

Supplementary Materials

Table S1. Attribute importance ranking of color.

Ranking	Descriptor Name	Description	Block
1	B05[F-X]	Presence/absence of F - X at topological distance 5	2D Atom Pairs
2	SddsAs	Sum of ddsAs E-states	Atom-type E-state indices
3	RDF155s	Radial Distribution Function - 155 / weighted by I-state	RDF descriptors
4	F08[O-Si]	Frequency of O - Si at topological distance 8	2D Atom Pairs
5	ATS5i	Broto-Moreau autocorrelation of lag 5 (log function) weighted by ionization potential	2D autocorrelations
6	CATS3D_14_NL	CATS3D Negative-Lipophilic BIN 14 (14.000 - 15.000 Å)	CATS 3D
7	F02[O-I]	Frequency of O - I at topological distance 2	2D Atom Pairs
8	SpMax4_Bh(s)	Largest eigenvalue n. 4 of Burden matrix weighted by I-state	Burden eigenvalues
9	F01[Br-Si]	Frequency of Br - Si at topological distance 1	2D Atom Pairs
10	ATSC3e	Centred Broto-Moreau autocorrelation of lag 3 weighted by Sanderson electronegativity	2D autocorrelations
11	E1e	1st component accessibility directional WHIM index / weighted by Sanderson electronegativity	WHIM descriptors
12	Mor27i	Signal 27 / weighted by ionization potential	3D-MoRSE descriptors
13	HVcpx	Graph vertex complexity index	Information indices
14	C-014	CX4	Atom-centred fragments
15	SpPosA_Dz(p)	Normalized spectral positive sum from Barysz matrix weighted by polarizability	2D matrix-based descriptors
16	nN	Number of Nitrogen atoms	Constitutional indices
17	F03[N-P]	Frequency of N - P at topological distance 3	2D Atom Pairs
18	CATS3D_00_DL	CATS3D Donor-Lipophilic BIN 00 (0.000 - 1.000 Å)	CATS 3D
19	VE1_B(m)	Coefficient sum of the last eigenvector (absolute values) from Burden matrix weighted by mass	2D matrix-based descriptors
20	B08[C-Br]	Presence/absence of C - Br at topological distance 8	2D Atom Pairs

21	F08[O-B]	Frequency of O - B at topological distance 8	2D Atom Pairs
22	B10[P-X]	Presence/absence of P - X at topological distance 10	2D Atom Pairs
23	B05[P-P]	Presence/absence of P - P at topological distance 5	2D Atom Pairs
24	B05[P-Cl]	Presence/absence of P - Cl at topological distance 5	2D Atom Pairs

Table S2. Attribute importance ranking of odor.

Ranking	Descriptor Name	Description	Block
1	B09[O-F]	Presence/absence of O - F at topological distance 9	2D Atom Pairs
2	Eig12_AEA(dm)	Eigenvalue n. 12 from augmented edge adjacency mat. weighted by dipole moment	Edge adjacency indices
3	DISPm	Displacement value / weighted by mass	Geometrical descriptors
4	F09[B-Si]	Frequency of B - Si at topological distance 9	2D Atom Pairs
5	CATS3D_10_NN	CATS3D Negative-Negative BIN 10 (10.000 - 11.000 Å)	CATS 3D
6	H8i	H autocorrelation of lag 8 / weighted by ionization potential	GETAWAY descriptors
7	F04[S-Br]	Frequency of S - Br at topological distance 4	2D Atom Pairs
8	J_B(i)	Balaban-like index from Burden matrix weighted by ionization potential	2D matrix-based descriptors
9	CATS3D_07_PL	CATS3D Positive-Lipophilic BIN 07 (7.000 - 8.000 Å)	CATS 3D
10	P_VSA_ppp_A	P_VSA-like on potential pharmacophore points, A - hydrogen-bond acceptor	P_VSA-like descriptor
11	F04[N-Cl]	Frequency of N - Cl at topological distance 4	2D Atom Pairs
12	VE1_Dt	Coefficient sum of the last eigenvector (absolute values) from detour matrix	2D matrix-based descriptors
13	F10[N-S]	Frequency of N - S at topological distance 10	2D Atom Pairs
14	F06[S-S]	Frequency of S - S at topological distance 6	2D Atom Pairs
15	D/Dtr08	Distance/detour ring index of order 8	Ring descriptors
16	DLS_02	Modified drug-like score from Oprea et al. (6 rules)	Drug-like indices
17	B10[P-Si]	Presence/absence of P - Si at topological distance 10	2D Atom Pairs
18	NddssSe	Number of atoms of type ddssSe	Atom-type E-state indices
19	SpAD_D	Spectral absolute deviation from topological distance matrix	2D matrix-based descriptors
20	L3m	3rd component size directional WHIM index / weighted by mass	WHIM descriptors
21	F10[C-Cl]	Frequency of C - Cl at topological distance 10	2D Atom Pairs

22	Chi_Dt	Randic-like index from detour matrix	2D matrix-based descriptors
23	RDF085v	Radial Distribution Function - 085 / weighted by van der Waals volume	RDF descriptors
24	VR3_Dz(Z)	Logarithmic Randic-like eigenvector-based index from Barysz matrix weighted by atomic number	2D matrix-based descriptors
25	HyWi_B(i)	Hyper-Wiener-like index (log function) from Burden matrix weighted by ionization potential	2D matrix-based descriptors
26	B05[P-Cl]	Presence/absence of P - Cl at topological distance 5	2D Atom Pairs
27	CATS3D_03_LL	CATS3D Lipophilic-Lipophilic BIN 03 (3.000 - 4.000 Å)	CATS 3D
28	HATS7i	Leverage-weighted autocorrelation of lag 7 / weighted by ionization potential	GETAWAY descriptors
29	ZM1	First Zagreb index	Topological indices
30	HATS6m	Leverage-weighted autocorrelation of lag 6 / weighted by mass	GETAWAY descriptors
31	Eig11_AEA(dm)	Eigenvalue n. 11 from augmented edge adjacency mat. weighted by dipole moment	Edge adjacency indices
32	CATS3D_14_NL	CATS3D Negative-Lipophilic BIN 14 (14.000 - 15.000 Å)	CATS 3D
33	nSO3OH	Number of sulfuric (thio-/dithio-) acids	Functional group counts
34	TDB04u	3D Topological distance based descriptors - lag 4 unweighted	3D autocorrelations
35	F10[P-Br]	Frequency of P - Br at topological distance 10	2D Atom Pairs
36	F08[O-B]	Frequency of O - B at topological distance 8	2D Atom Pairs
37	JGI4	Mean topological charge index of order 4	2D autocorrelations
38	P_VSA_MR_8	P_VSA-like on Molar Refractivity, bin 8	P_VSA-like descriptor
39	CATS3D_09_NN	CATS3D Negative-Negative BIN 09 (9.000 - 10.000 Å)	CATS 3D

Table S3. The results for each fold in the 4-fold cross-validation.

Task	Method	Number of features	Mean accuracy	Accuracy (4-fold cross-validation)
Color perception	Random forest	① All features	100%	100%, 100%, 100%, 100%
		② 24 features #	100%	100%, 100%, 100%, 100%
		③ 24 features *	99.45%	99.37%, 99.69%, 99.36%, 99.37%
		② + ③features	100%	100%, 100%, 100%, 100%
	DBN	① All features	95.23%	95.89%, 94.93%, 94.88%, 95.23%
		② 24 features #	47.35%	47.95%, 49.06%, 46.50%, 45.89%
		③ 24 features *	44.20%	44.16%, 43.75%, 44.59%, 44.30%
		② + ③features	48.79%	53.63%, 44.69%, 50.00%, 46.84%
Odor perception	Random forest	① All features	93.40%	93.33%, 93.92%, 93.20%, 93.15%
		② 39 features #	93.40%	93.33%, 93.92%, 93.20%, 93.15%
		③ 39 features *	93.38%	93.33%, 93.90%, 93.18%, 93.11%
		② + ③features	93.40%	93.33%, 93.92%, 93.20%, 93.15%
	DBN	① All features	94.75%	95.24%, 94.35%, 94.27%, 95.13%
		② 39 features #	36.18%	43.33%, 30.41%, 36.73%, 34.25%
		③ 39 features *	31.46%	31.29%, 31.33%, 29.00%, 34.25%
		② + ③features	42.44%	48.00%, 38.51%, 48.30%, 34.93%

Random forest models

* The combination of random forest models and genetic algorithm

Supplementary Data1. The datasets of the 1267 structurally diverse molecules labeled with 12 diverse colors and 5270 molecular descriptors.

Supplementary Data2. The datasets of the 598 structurally diverse molecules labeled with 12 diverse odors and 5270 molecular descriptors.

Supplementary Data3. The datasets of the 90 molecules with both color and odor information.

Figure

Click here to access/download;Figu

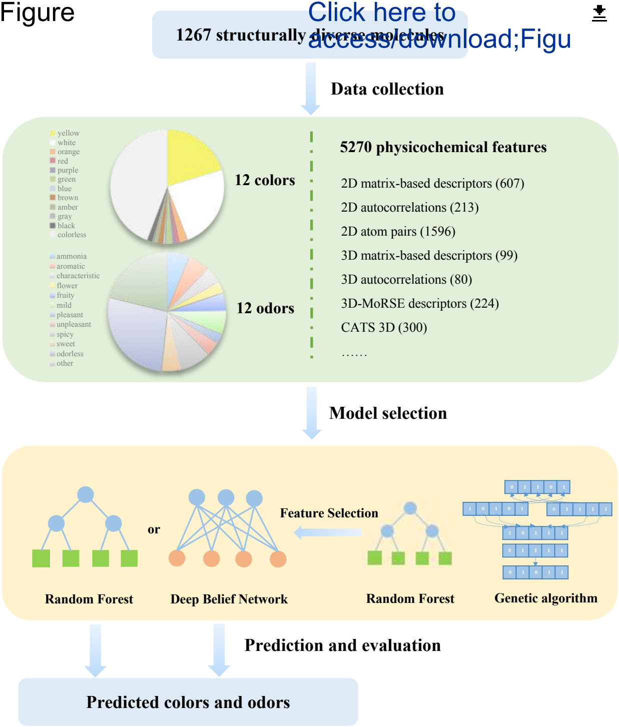

Figure

Click here to access/download;Figu

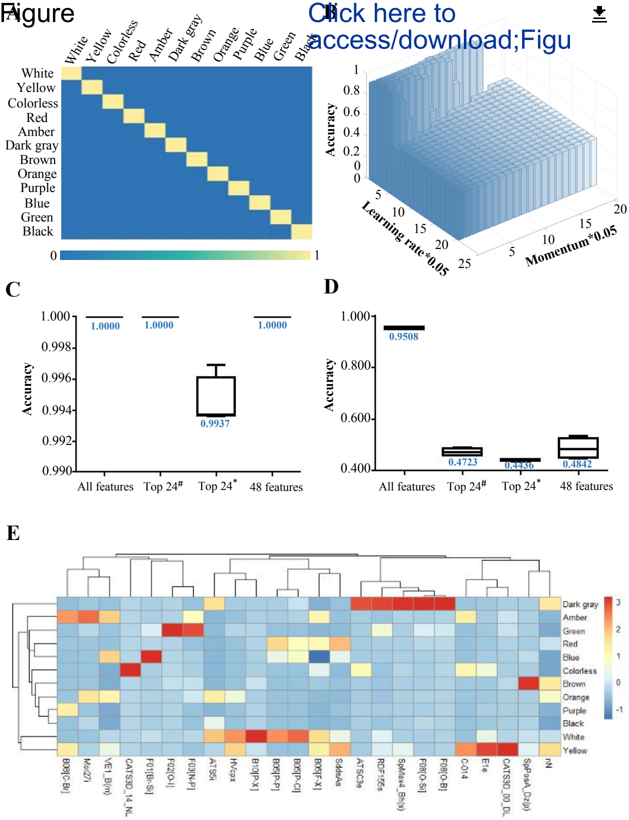

Figure

Click here to access/download;Figu

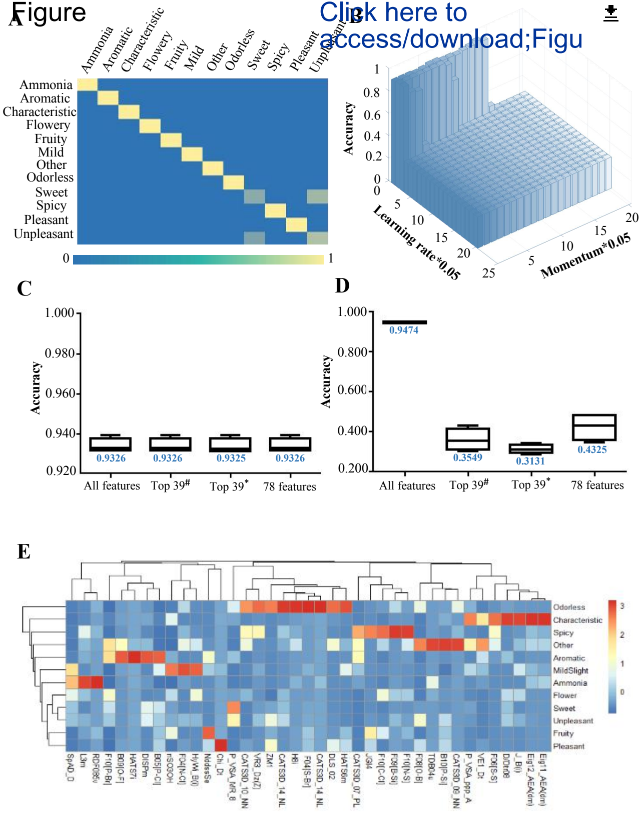

Figure

Click here to access/download;Figu

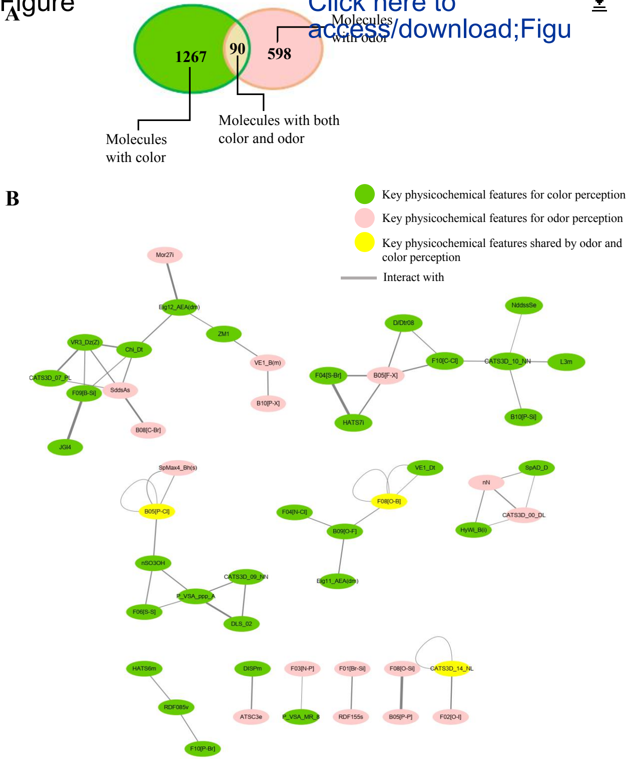

Figure

Click here to access/download;Figu

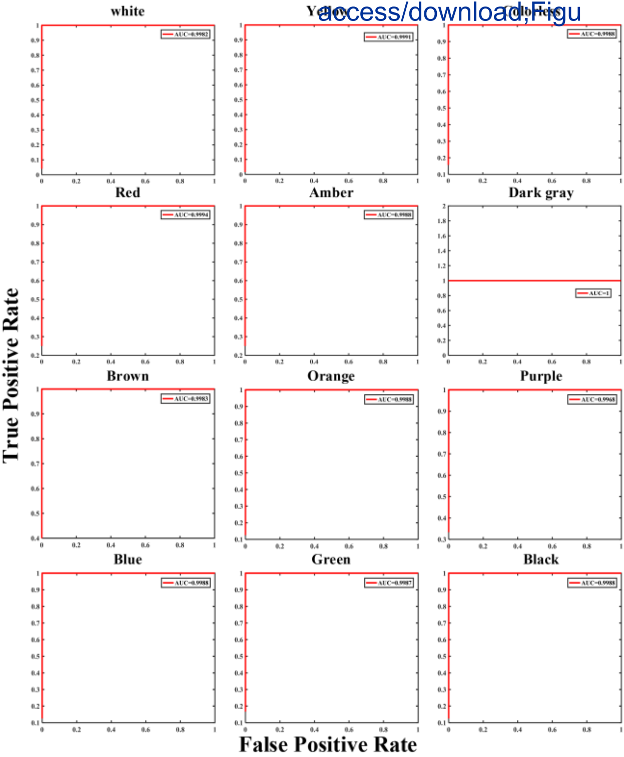

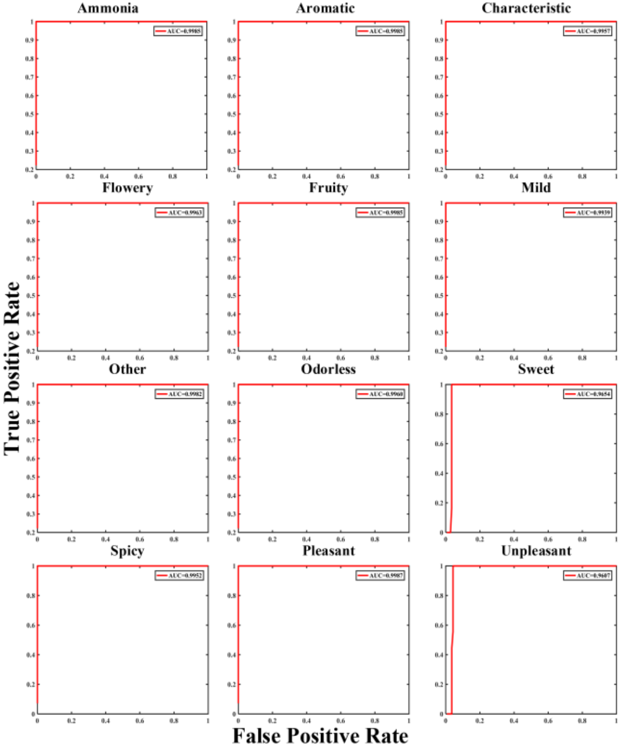
Ammonia
AUC=0.9985
Aromatic
AUC=0.9985
Characteristic
AUC=0.9957
Flowery
AUC=0.9963
Fruity
AUC=0.9985
Mild
AUC=0.9939
Other
AUC=0.9982
Odorless
AUC=0.9960
Sweet
AUC=0.9654
Spicy
AUC=0.9952
Pleasant
AUC=0.9987
Unpleasant
AUC=0.9607
True Positive Rate
False Positive Rate

Click here to access/download
Supplementary Material
Supplementary Data1.xlsx

Click here to access/download
Supplementary Material
Supplementary Data2.xlsx

Supplementary Data3

Click here to access/download
Supplementary Material
Supplementary Data3.xlsx

Revision Cover Letter

Click here to access/download;Personal Cover;Revision Cover Letter.doc

Haotian Lin, M.D., Ph.D.
State Key Laboratory of Ophthalmology
Zhongshan Ophthalmic Center
Sun Yat-sen University
Tel: +86-13802793086
E-mail: haot.lin@hotmail.com

Dec 11, 2019

Dear Scott Edmunds,

Thank you so much for your decision letter on our manuscript entitled "Artificial intelligence deciphers codes for color and odor perceptions based on large-scale chemoinformatic data". We sincerely treasure all the constructive and crucial suggestions from you and reviewers. Based on the provided suggestions and comments, we have addressed all the issues carefully and revised our manuscript accordingly. We hope that the revised manuscript can convince you and reviewers.

The main improvements and revisions are as follows:

1) The method for feature selection in color and odor prediction was improved by comparing random forest algorithm and the combination of the genetic algorithm and random forest algorithm. The corresponding results were changed (Line 148-150, Line 166-168, Figure 2C-E, Figure 3C-E, Figure 4B and Table S1-S3). However, it had no effect on our main results.
2) Additional results of the random forest evaluated by area under the curve were supplied in Supplementary Materials (Figure S1). More rigorous expressions were completed according to the constructive suggestions from Reviewer 2.

Thank you once again for your agreement and help with our research. We believe that our findings based on large-scale physicochemical features of structurally diverse molecules could offer new insight into the understanding of color and odor sensing.

Sincerely yours,

Haotian Lin, on behalf of all authors

Response to Reviewers Click here to access/download;Personal Cover;Response to Reviewers.docx

'Response to Reviewers' files GIGA-D-19-00112R1

Artificial intelligence deciphers codes for color and odor perceptions based on large-scale chemoinformatic data

GigaScience

Dear Scott Edmunds and Reviewers,

Thank you so much for the agreements and insightful suggestions on our manuscript. The following are our point-by-point responses to the reviewers' comments and corresponding changes are marked in the revised manuscript. We hope that we have addressed all the suggestions adequately.

Our point-by-point responses are as follows:

Reviewer #1: ***Comment (1):*** *Thank you for revising and improving the manuscript. The authors have addressed my previous concerns. I still have a few comments about the revised manuscript: In Figure 3A, it seems the model cannot distinguish "sweet" and "unpleasant" very well. But in general, "sweet" should be a "pleasant" odor instead of an "unpleasant" one. How can these results be reconciled?*

Response: Thanks so much for your agreement on the merit of our work, as well as your constructive comments. We considered olfactory perception vary greatly among individuals, as your study discovered that the perceived attributes were rated differently among individuals, which considerably complicated the prediction challenge (*GigaScience* 2017; 7, 1–11). So in this study we emphasized the selection of molecules with definite color or odors were defined by NCBI (Line 103-104, 210-212). If the molecule is defined as "sweet" in NCBI, we try not to classify this "sweet" as a "pleasant sweet" or an "unpleasant sweet" according to our subjective feelings. And molecules with multiple odors that are difficult to define by NCBI were excluded.

We certainly agree that this process may reduce the credibility of results in the real world, and bring about the results that odor sensing was less accurate than that of color in our study (Discussion, Line 217-221). However, rather than arranging large-scale human resources

from different races to distinguish odors “fairly”, we prefer this way to deal with the data.

Comment (2): *For Figure 2C-D and Figure 3C-D, it would be clearer if the median values of these boxplots are labeled.*

Response: Thank you again for your acceptance and all of the helpful comments. We have labeled the median values in the boxplots in Figure 2C-D and Figure 3C-D. The figure legends have been modified correspondingly (Line 457-465, 474-483).

Reviewer #2:

Comment (1): *Figure 1: from the figure itself it looks like that the feature selection was done separately. However, to test the performance of the classifiers based on feature selected by the genetic algorithm random forest and DBN model were used again. This is unclear for this figure but clear in the text.*

Response: Thanks so much for your constructive comments and suggestions for our study. We have rearranged Figure 1 to show the random forest and DBN models were used for a second time after feature selection. The figure legend has been modified correspondingly (Line 446-449).

Comment (2): *Usually, a feature selection step is performed before model building and random forest can perform this selection on its own. I am not convinced with the response of the author that due to the sparsity of the matrix and huge physicochemical data they did not discuss the feature importance results from random forest however they consider random forest performs the best. This aspect should be discussed in the publication.*

Response: We really appreciate your constructive suggestions. We have revised our method applying random forest models to perform feature selection on its own, and found that random forest alone did even better than combining it with the genetic algorithm using the same number of features (24 features for color perception, 39 features for odor perception). The results for each fold in the 4-fold cross-validation are shown below comparing two methods of feature selection after our correction, and the best performing results we used in

the revised results are highlighted.

Table S3. The results for each fold in the 4-fold cross-validation.

Task	Method	Number of features	Mean accuracy	Accuracy (4-fold cross-validation)
Color perception	Random forest	① All features	100%	100%, 100%, 100%, 100%
		② 24 features #	100%	100%, 100%, 100%, 100%
		③ 24 features *	99.45%	99.37%, 99.69%, 99.36%, 99.37%
		② + ③features	100%	100%, 100%, 100%, 100%
	DBN	① All features	95.23%	95.89%, 94.93%, 94.88%, 95.23%
		② 24 features #	47.35%	47.95%, 49.06%, 46.50%, 45.89%
		③ 24 features *	44.20%	44.16%, 43.75%, 44.59%, 44.30%
		② + ③features	48.79%	53.63%, 44.69%, 50.00%, 46.84%
Odor perception	Random forest	① All features	93.40%	93.33%, 93.92%, 93.20%, 93.15%
		② 39 features #	93.40%	93.33%, 93.92%, 93.20%, 93.15%
		③ 39 features *	93.38%	93.33%, 93.90%, 93.18%, 93.11%
		② + ③features	93.40%	93.33%, 93.92%, 93.20%, 93.15%
	DBN	① All features	94.75%	95.24%, 94.35%, 94.27%, 95.13%
		② 39 features #	36.18%	43.33%, 30.41%, 36.73%, 34.25%
		③ 39 features *	31.46%	31.29%, 31.33%, 29.00%, 34.25%
		② + ③features	42.44%	48.00%, 38.51%, 48.30%, 34.93%

Random forest models

* The combination of random forest models and genetic algorithm

According to the new features selected by random forest models, we have made changes in the results section (Line 148-150, Line 166-168, Figure 2C-E, Figure 3C-E, Figure 4B and Table S1-S3). In the method section, we described the application of the two methods in detail (Line 280-299).

Comment (3): *The results of the random forest are very impressive and confusing at the same time. I am unsure how can random forest achieve 100% accuracy with a sparse dataset as you mentioned in the comment for 12 different colours class. Because random forest -- bagging and suboptimal selection of splits may waste most of the model insight on zero-only areas. It will be very informative if you could show AUC for each class individually.*

Response: Thanks for your suggestion. The AUCs of the random forest in the prediction of twelve colors and twelve odors using all features are shown below. The figure has also been

added to the Supplementary Materials (Figure S1).

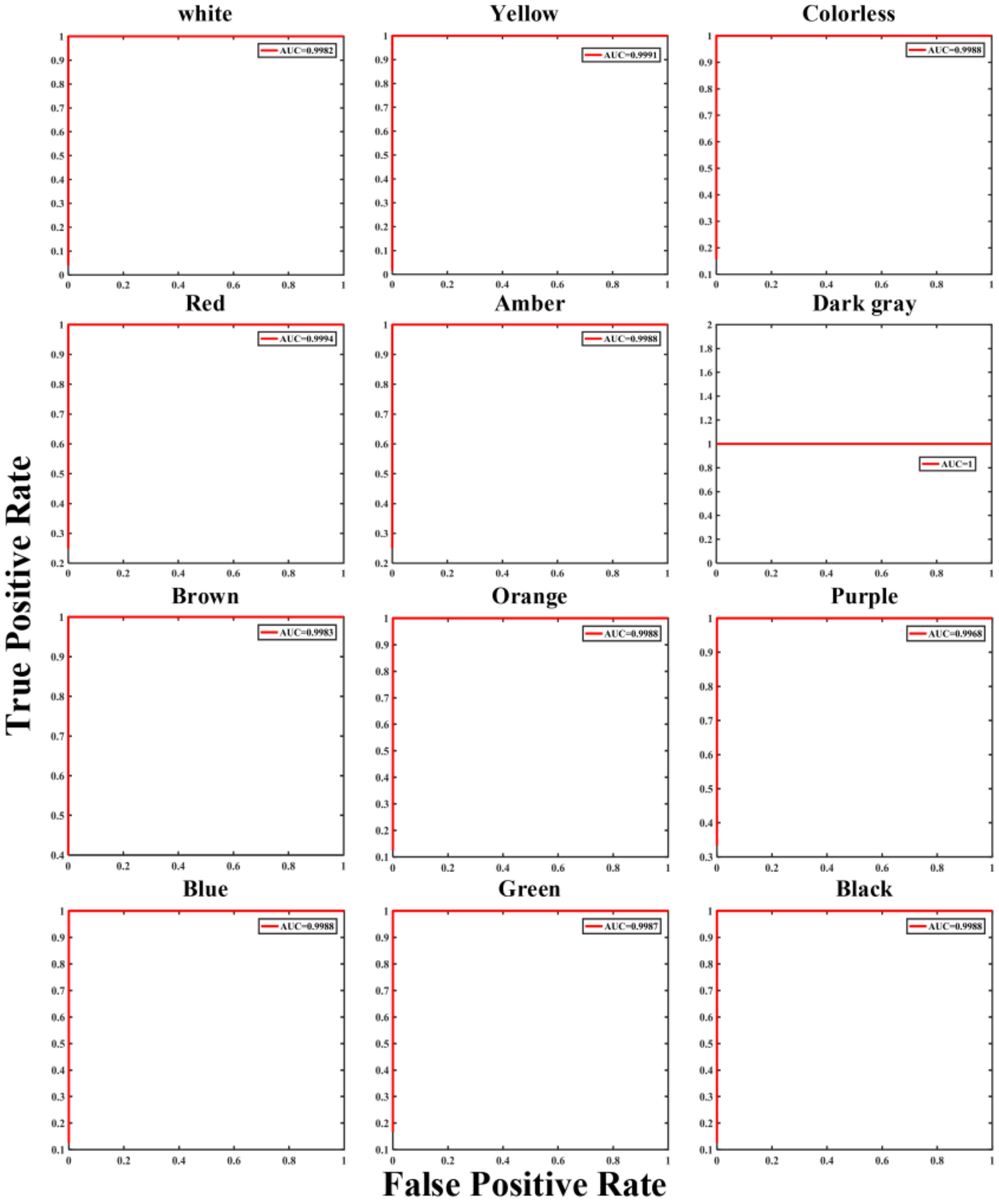

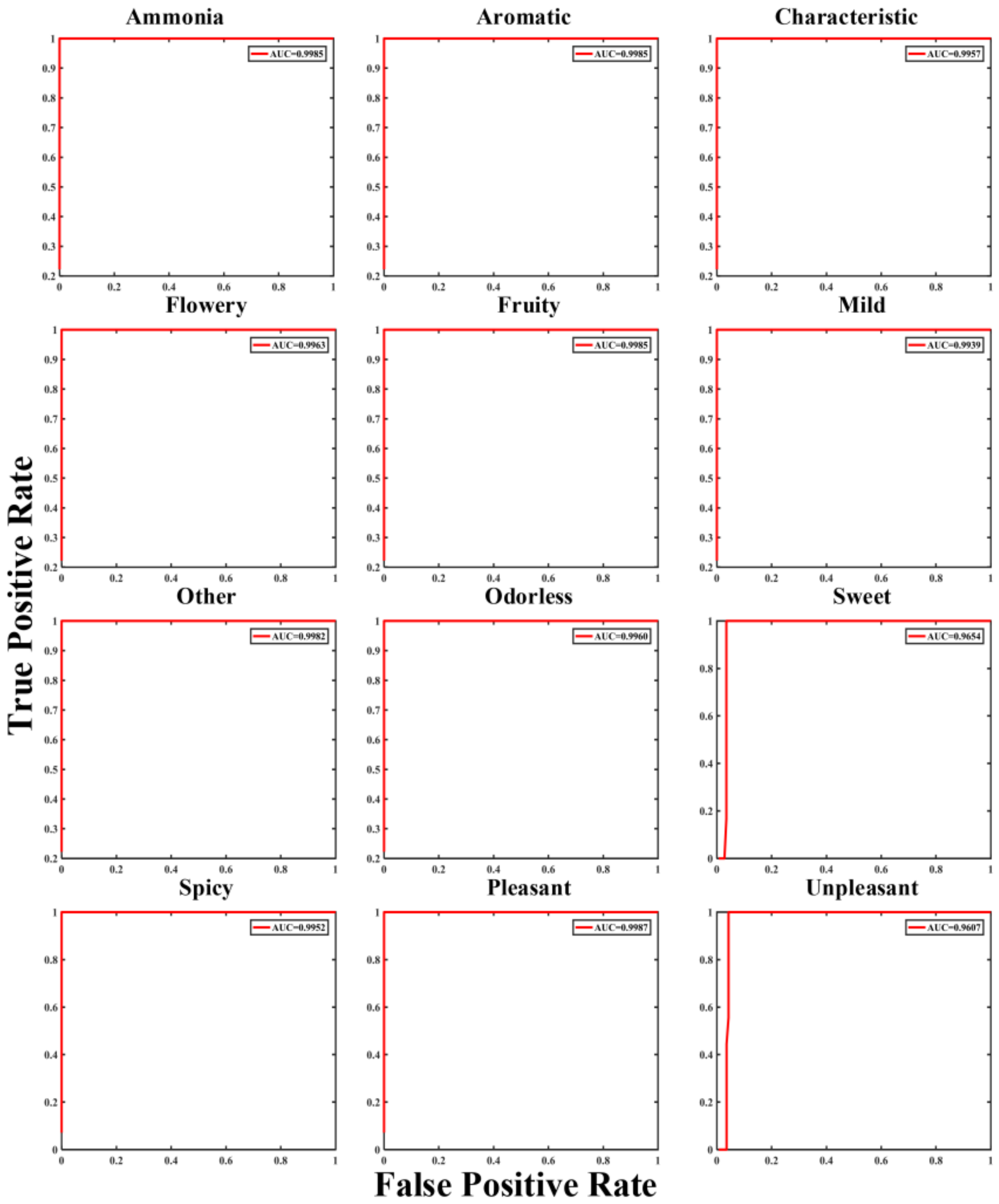

Figure S1. The prediction accuracies of random forest models for twelve colors and twelve odors using all features. AUC, area under the curve.

Table S4. The prediction accuracies of random forest models for twelve colors and twelve odors using all features. AUC, area under the curve.

Task	Label	AUC	Task	Label	AUC
Color perception	White	0.9982	Odor perception	Ammonia	0.9985
	Yellow	0.9991		Aromatic	0.9985
	Colorless	0.9988		Characteristic	0.9957
	Red	0.9994		Flowery	0.9963
	Amber	0.9988		Fruity	0.9985
	Dark gray	1.0000		Mild	0.9939
	Brown	0.9983		Other	0.9982
	Orange	0.9988		Odorless	0.9960
	Purple	0.9968		Sweet	0.9654
	Blue	0.9988		Spicy	0.9952
	Green	0.9987		Pleasant	0.9987
	Black	0.9988		Unpleasant	0.9607

We confirmed the earlier result based on a re-examination of the source code (https://github.com/Hugo0512/ColorOdorprediction).

Comment (4): *line 145: "The data was divided into the training and testing data sets without oversampling" - could mention the proportion of your data split into training and testing?*

Response: Many thanks for your comment. We re-emphasized the classification method of training and testing data sets was *k*-fold cross-validations (k = 4) in this line (Line 131-132).

Comment (5): *line 194-195: Pearson correlation coefficients > 0.1958, please write the exact coefficient value.*

Response: Thanks so much for your scrupulous correction. The minimum absolute value of the Pearson correlation coefficients applied in this revised vision was 0.300552 (Line 180-181).

Comment (6): *Figure 4: A) the intersecting part of the pie chart can be a different colour. Before Figure 4, there is no explanation of vital features. It will be useful to include the definition of the vital feature before or while writing the result on the correlation between colour and odour.*

Response: Thanks so much for your scrupulous correction. The color in Figure 4A has been

modified. We also unified our expression using “key physicochemical features” (Line 92, 94, 148, 167, 195, 488, Figure 4B), and defined it as the physicochemical descriptors contributed most to the predictive accuracies (Line 90-93, 301-303).

Comment (7): *Line 209-210: It will be helpful for the readers if the authors discuss the rationale behind the connected colour and odour physicochemical features.*

Response: Thanks for your suggestion. The shared and prominently interlinked key physicochemical features identified in predicting color and odor have not been reported yet. We cautiously discussed the significance in Page 10, Line 199-202.

Comment (8): *Line 215: it would be interesting to show the evaluation scores from the DREAM challenge participants to directly compare the results from your claim.*

Response: Thanks for your suggestion. We agree that the evaluation scores from the DREAM challenge participants should be mentioned in the discussion (Line 203-205). Actually, there were great differences in data types and evaluation criteria between our study and the DREAM challenge. We solved one question with a multi category for each time here, while multiple questions are classified together in the DREAM challenge. The evaluation scores we used were accuracies, kappa coefficients and AUCs, the evaluation in DREAM challenge was based on the Pearson’s correlation between observed and predicted perceptions.

Comment (9): *line 221, is a redundant sentence instead you can write - "The winning algorithm of the DREAM challenge indicated that the random forest outperforms other base learning methods. "*

Response: Thanks so much for your scrupulous correction. We removed the statement accordingly in Line 208-210.

Comment (10): *line 228 no comma needed*

Response: Thanks so much for your scrupulous correction. The sentence has been removed in the revised version.

Comment (11): *line 461: and is missing in this line - "all features and the top 24 features".*

Response: Thanks so much for your scrupulous correction. We have modified the figure legends (Line 457-465).

Comment (12): *line 202, as a reader it would be informative to get the definition of terse framework or at least a citation?*

Response: Thanks so much for your scrupulous correction. We removed the word “terse” to avoid misunderstanding (Line 188).

Comment (13): *The cutoff to decide the number of features is not clear in the manuscript.*

Response: Many thanks for your comment. In order to compare random forest algorithm itself with the combination of the genetic algorithm and random forest algorithm, the numbers of the key features selected were similar. As random forest model could not choose the cut off by itself, the cutoff to decide the number of features was determined by the genetic feature selection task. After running the genetic feature selection task 20 times, 24 descriptors were selected 18 times with a classification accuracy of 100% ± 0.0% in the random forest model for color, 39 descriptors were selected 16 times with a classification accuracy of 93.38% ± 0.31% in the random forest model for odor. (Method, Line 287-291, 296-299).

--

Finally, thank you again for your acceptance and all of the helpful comments, and we hope that you will now find our revisions suitable for publication.

Sincerely yours,

Haotian Lin on behalf of all authors
