# Supplementary material for: Artificial intelligence deciphers codes for color and odor perceptions based on large-scale chemoinformatic data
Source: Gigascience. 2020 Feb 26;9(2):giaa011. doi: 10.1093/gigascience/giaa011 (PMC7043059; doi:10.1093/gigascience/giaa011)
Supplement: giaa011_GIGA-D-19-00112_Revision_3 [file giaa011_giga-d-19-00112_revision_3.pdf]

# GigaScience

## Artificial intelligence deciphers codes for color and odor perceptions based on large-scale chemoinformatic data

--Manuscript Draft--

| | |
|---|---|
| **Manuscript Number:** | GIGA-D-19-00112R3 |
| **Full Title:** | Artificial intelligence deciphers codes for color and odor perceptions based on large-scale chemoinformatic data |
| **Article Type:** | Research |
| **Funding Information:** | National Key Research and Development Program of China (2018YFC0116500) — Prof. Haotian Lin<br>National Natural Science Foundation of China (81770967) — Prof. Haotian Lin<br>National Natural Science Foundation of China (81822010) — Prof. Haotian Lin<br>the Key Research and Development Program of Guangdong Province (No. 2018B010109008) — Prof. Haotian Lin |
| **Abstract:** | Background<br>Color vision is the ability to detect, distinguish, and analyze the wavelength distributions of light independent of the total intensity. It mediates the interaction between an organism and its environment from multiple important aspects. However, the physicochemical basis of color coding has not been explored completely, and how color perception is integrated with other sensory input, typically odor, is unclear.<br>Results<br>Here, we developed an artificial intelligence platform to train algorithms for distinguishing color and odor based on the large-scale physicochemical features of 1267 and 598 structurally diverse molecules, respectively. The predictive accuracies achieved using the random forest and deep belief network for the prediction of color were 100.0% ± 0.0% and 95.23% ± 0.40% (mean ± SD), respectively. The predictive accuracies achieved using the random forest and deep belief network for the prediction of odor were 93.40% ± 0.31% and 94.75% ± 0.44% (mean ± SD), respectively. Twenty-four physicochemical features were sufficient for the accurate prediction of color, while thirty-nine physicochemical features were sufficient for the accurate prediction of odor. A positive correlation between the color coding and odor coding properties of the molecules was predicted. A group of descriptors was found to interlink prominently in color and odor perceptions.<br>Conclusions<br>Our random forest model and DBN accurately predicted the colors and odors of structurally diverse molecules. These findings extend our understanding of the molecular and structural basis of color vision and reveal the interrelationship between color and odor perceptions in nature. |
| **Corresponding Author:** | Haotian Lin, Ph.D., M.D.<br>Sun Yat-Sen University Zhongshan Ophthalmic Center<br>CHINA |
| **Corresponding Author Secondary Information:** | |
| **Corresponding Author's Institution:** | Sun Yat-Sen University Zhongshan Ophthalmic Center |
| **Corresponding Author's Secondary Institution:** | |
| **First Author:** | Xiayin Zhang |
| **First Author Secondary Information:** | |
| **Order of Authors:** | Xiayin Zhang |

| | |
|---|---|
| | Kai Zhang |
| | Duoru Lin |
| | Yi Zhu |
| | Chuan Chen |
| | Lin He |
| | Xusen Guo |
| | Kexin Chen |
| | Ruixin Wang |
| | Zhenzhen Liu |
| | Xiaohang Wu |
| | Erping Long |
| | Kai Huang |
| | Zhiqiang He |
| | Xiyang Liu |
| | Haotian Lin, Ph.D., M.D. |
| **Order of Authors Secondary Information:** | |
| **Response to Reviewers:** | Dear Edmunds and Reviewers,<br><br>Thank you so much for your agreement and clear corrections on our manuscript. The following are our point-by-point responses to the reviewer's comments and corresponding changes are marked in the revised manuscript. We hope that we have addressed your concern adequately.<br><br>----------------------------------<br><br>Our point-by-point responses are as follows:<br><br>Reviewer #2: Comment (1): Thank you for the second revision. It is quite impressive to see prediction accuracies of random forest models for predicting individual colours and odours. It is clear from your results (Table S3) that DBN might choose different sets of features in order to perform better because DBN can reach to 95% accuracy but fails to deliver best performance for 24 features chosen by random forest/genetic algorithm. Can you please make a short discussion on this point in your manuscript. This will reflect on the poor performance of DBN.<br>Response: Thanks so much for your insightful suggestions on our manuscript all along. We considered that there were two main reasons why DBN failed to deliver best performance with the 24 features. Firstly, the key physicochemical features identified in predicting color and odor were selected by random forest and genetic algorithm. These features may not be suitable for DBN,which can map the raw data to low-dimensional space by unsupervised learning in the pre-training phase. Secondly, although DBN is a multi-layered recurrent neural network trained with energy minimizing methods, the network structure of DBN has a great influence on the learning performance. The network structure of DBN utilizing all features and the key physicochemical features are exactly the same in our study. If other algorithm like the Particle Swarm Optimization Algorithm could be used to optimize the number of DBN hidden-layer nodes, the performance of DBN network may improve. We have further discussed these potential reasons for the performance of DBN in Line 211-222.<br><br>Comment (2): line 115. specify the supplementary data table number.<br>Response: Thank you for your scrupulous correction. "Supplementary Data1-3" has been added in Line 114.<br><br>Comment (3): line 145-148 is not clear. As you stated the sentence saying random forest algorithm and the combination of the genetic algorithm and random forest |

Powered by Editorial Manager® and ProduXion Manager® from Aries Systems Corporation

| | |
|---|---|
| | algorithm..., where the second part of the sentence explains the advantage of random forest. This leaves the first part incomplete.<br>Response: We really appreciate your constructive suggestions. To highlight the best classification accuracy with 24 key physicochemical features selected by random forest in the results section, we have removed the description of feature selection methods here and revised the sentence in the method section (Line 288-290).<br><br>Comment (4): line 195-196. the sentence is misleading to suggest that physicochemical features are connected. This needs more clarity.<br>Response: Many thanks for your critical suggestion. We modified this sentence to "Our findings also suggested that key physicochemical features in distinguishing color and odor are significantly correlated" (Line 192-193).<br><br>Comment (5): line 199-202, this sentence is also misleading because the outcome of the edges between the two features is not explained properly. One can see that due to the correlation there are two features connected but there is no evidence suggesting that they influence changing the odour and colour.<br>Response: Thanks for your suggestion. We have changed the statement in a more conservative manner (Line 197-199).<br><br>Comment (6): There are some places where you mention random forest algorithm and the combination of the genetic algorithm and random forest algorithm. this is very confusing. I think you want to say that for feature selection random forest and genetic algorithm were used. Can you please make this consistent.<br>Response: Thank you again for your helpful corrections. We have made modifications to unify the descriptions for feature selection (Line 91, Line 287, Line 454, Line 467).<br><br><br>--------------------------------------------------------<br>Finally, thank you again for all of the helpful comments, and we hope that you will now find our revisions suitable for publication.<br><br>Sincerely yours,<br>Haotian Lin on behalf of all authors |
| **Additional Information:** | |
| **Question** | **Response** |
| Are you submitting this manuscript to a special series or article collection? | No |
| **Experimental design and statistics**<br><br><br>Full details of the experimental design and statistical methods used should be given in the Methods section, as detailed in our Minimum Standards Reporting Checklist. Information essential to interpreting the data presented should be made available in the figure legends.<br><br><br>Have you included all the information requested in your manuscript? | Yes |
| **Resources** | Yes |

| | |
|---|---|
| A description of all resources used, including antibodies, cell lines, animals and software tools, with enough information to allow them to be uniquely identified, should be included in the Methods section. Authors are strongly encouraged to cite Research Resource Identifiers (RRIDs) for antibodies, model organisms and tools, where possible.<br><br>Have you included the information requested as detailed in our Minimum Standards Reporting Checklist? | |
| **Availability of data and materials**<br><br>All datasets and code on which the conclusions of the paper rely must be either included in your submission or deposited in publicly available repositories (where available and ethically appropriate), referencing such data using a unique identifier in the references and in the “Availability of Data and Materials” section of your manuscript.<br><br>Have you have met the above requirement as detailed in our Minimum Standards Reporting Checklist? | Yes |

*Powered by Editorial Manager® and ProduXion Manager® from Aries Systems Corporation*

Manuscript

Click here to access/download;Manuscript;Manuscript (with changes tracked).docx

# Artificial intelligence deciphers codes for color and odor perceptions based on large-scale chemoinformatic data

Xiayin Zhang[1†] (zhangxiayin@gzzoc.com), Kai Zhang[1,2†] (hugo88315@163.com), Duoru Lin[1†] (linduoru@sina.com), Yi Zhu[1,3] (y.zhu17@med.miami.edu), Chuan Chen[1,4] (c.chen30@med.miami.edu), Lin He[2] (August_us@163.com), Xusen Guo[5] (guoxs3@mail2.sysu.edu.cn), Kexin Chen[1] (873490288@qq.com), Ruixin Wang[1] (ruiruiw413@aliyun.com), Zhenzhen Liu[1] (liu_zhenzhen@qq.com), Xiaohang Wu[1] (1034281949@qq.com), Erping Long[1] (longerping@qq.com), Kai Huang[5] (huangk36@mail.sysu.edu.cn), Zhiqiang He[6] (hezq@bupt.edu.cn), Xiyang Liu[2] (xyliu@xidian.edu.cn) and Haotian Lin[1,7*] (haot.lin@hotmail.com).

[1]State Key Laboratory of Ophthalmology, Zhongshan Ophthalmic Center, Sun Yat-sen University, Guangzhou 510060, China;

[2]School of Computer Science and Technology, Xidian University, Xi'an 710000, China;

[3]Department of Molecular and Cellular Pharmacology, University of Miami Miller School of Medicine, Miami, Florida 33136, USA;

[4]Sylvester Comprehensive Cancer Center, University of Miami Miller School of Medicine, Miami, Florida 33136, USA;

[5]Key Laboratory of Machine Intelligence and Advanced Computing, Ministry of Education School of Data and Computer Science, Sun Yat-Sen University;

[6]Key Laboratory of Universal Wireless Communications, Beijing University of Posts and Telecommunications, Beijing 100876, China.

[7]Center of Precision Medicine, Sun Yat-sen University, Guangzhou 510080, China.

[†] These authors contributed equally to this work.

* **Corresponding Author:**

Prof. Haotian Lin

Xian Lie South Road 54#, Guangzhou, China, 510060

Telephone: +86-13802793086

Email address: haot.lin@hotmail.com

ORCID IDs:
Xiayin Zhang: 0000-0003-0250-0247; Erping Long: 0000-0002-3502-5596; Haotian Lin: 0000-0003-4672-9721


# Abstract

**Background**

Color vision is the ability to detect, distinguish, and analyze the wavelength distributions of light independent of the total intensity. It mediates the interaction between an organism and its environment from multiple important aspects. However, the physicochemical basis of color coding has not been explored completely, and how color perception is integrated with other sensory input, typically odor, is unclear.

**Results**

Here, we developed an artificial intelligence platform to train algorithms for distinguishing color and odor based on the large-scale physicochemical features of 1267 and 598 structurally diverse molecules, respectively. The predictive accuracies achieved using the random forest and deep belief network for the prediction of color were 100.0% ± 0.0% and 95.23% ± 0.40% (mean ± SD), respectively. The predictive accuracies achieved using the random forest and deep belief network for the prediction of odor were 93.40% ± 0.31% and 94.75% ± 0.44% (mean ± SD), respectively. Twenty-four physicochemical features were sufficient for the accurate prediction of color, while thirty-nine physicochemical features were sufficient for the accurate prediction of odor. A positive correlation between the color coding and odor coding properties of the molecules was predicted. A group of descriptors was found to interlink prominently in color and odor perceptions.

**Conclusions**

Our random forest model and DBN accurately predicted the colors and odors of structurally diverse molecules. These findings extend our understanding of the molecular and structural basis of color vision and reveal the interrelationship between color and odor perceptions in nature.

**Keywords:** color perception; odor perception; random forest; deep belief network; physicochemical features.

## Background

Color vision mediates the relationship between an organism and its environment in multiple important ways, including influencing mate choice, camouflage, and speciation [1]. We see a colorful world because different objects are composed of materials with different reflectance spectra in the wavelength range visible to our eyes [2]. Although knowledge of fundamental optical processes such as reflection, refraction, interference, diffraction, and scattering is accumulating [3], we lack the ability to recognize the color of cellular structure and pattern formation at optical scales from nanometers to microns.

Nature creates various colorful materials based on physicochemical properties including topological and geometrical properties that humans cannot easily see [4, 5]. For instance, the color changes from bright yellow through reddish–purple to blue when the size of a gold sample is decreased [6]. The different colors of disubstituted benzenes were discovered to be related to differences in the molecular structure with ortho, meta and para substitutions [7, 8]. The odors of chemicals are also fully encoded within their specific physicochemical properties [9, 10]. The compositions and structures of functional groups have been suggested to be crucial for the perception of aroma [11]. Moreover, evidence of the interaction between color vision and olfaction has been discovered [12]. For example, the odor of a host plant can modify the color sensed by a swallowtail butterfly [13]. The odor of wine can be predicted according to its color [14]. Additionally, the perceived intensity of an odor is positively correlated with the intensity of color [15, 16]. Neuroimaging and

repetitive transcranial magnetic stimulation studies showed that high-level odor processing also actives the visual cortex [17, 18]. However, the relationship between color and odor in terms of molecular physicochemical properties is largely unknown.

Artificial intelligence (AI) tools can be optimized to infer the innate laws of natural processes through machine learning tasks based on large-scale data sets and make predictions of the unknown [19, 20]. In the chemical sciences, AI has been used to guide chemical and material design, synthesis, characterization, and modeling [21, 22]. Previous researchers have equipped AI with a “nose” to predict human olfactory perception from the physicochemical features of 476 molecules and 21 perceptual attributes perceived by 49 individuals [23].

Here, we developed a random forest model and deep belief network (DBN) to predict the colors and odors of chemicals based on their molecular descriptors. We applied random forest and genetic algorithm for feature selection to identify the key physicochemical features that contribute most to the predictive accuracies. In addition, we investigated the connection between the key physicochemical features in color and odor coding to unravel the commonality between visual and olfactory perception.

## Data Description

**Data collection and labeling.** A total of 1267 structurally diverse molecules was used for color prediction in this study, and 598 structurally diverse molecules were used for odor prediction. The color, odor and three-dimensional (3D) structure data of these molecules were all collected from the key chemical information resource at the U.S.

National Center for Biotechnology Information, PubChem [24] (https://pubchem.ncbi.nlm.nih.gov) between June 1, 2017, and November 30, 2017. Molecules with definite colors or odors were defined from PubChem, and molecules with multiple colors or odors that are difficult to define were excluded. The data set of colors was classified into 12 diverse colors, including yellow (257 molecules), white (301 molecules), orange (31 molecules), red (16 molecules), purple (11 molecules), green (24 molecules), blue (9 molecules), brown (20 molecules), amber (15 molecules), gray (6 molecules), black (17 molecules) and colorless (560 molecules). The data set of odors was classified into 12 diverse odors, including ammonia (37 molecules), aromatic (36 molecules), characteristic (27 molecules), flower (19 molecules), fruity (29 molecules), mild (38 molecules), other (127 molecules), pleasant (16 molecules), unpleasant (23 molecules), spicy (54 molecules), sweet (30 molecules) and odorless (162 molecules).

**Physicochemical features of the molecules.** The PubChem compound identifier for each molecule was provided (Supplementary data 1-3). We applied the commercial chemoinformatics software package Dragon (version 7.0, https://chm.kode-solutions.net/products_dragon_papers.php) to calculate 5270 physicochemical descriptors for each of the molecules, including the simplest atom types, functional groups and fragment counts, topological and geometrical descriptors, 3D descriptors, several property estimations (such as log*P*) and drug-like and lead-like alerts (such as the Lipinski's alert). These molecular descriptors are formal mathematical representations of a molecule and include their definition, symbols and

labels, formulas, some numerical examples, data, and molecular graphs, as presented in the Handbook of Molecular Descriptors [25]. The missing values marked as “NaN” simply mean that for these molecules, some descriptors have not been calculated for some reason, which is common because several descriptors have particular constraints. Molecules with more than 2000 descriptors marked as “NaN” were not used. We replaced all of the “NaN” entries with “0” during the dataset preprocessing. For molecules with color, the average number of “NaN” within 5270 descriptors was 353 per molecule. For molecules with odor, the average number of “NaN” within 5270 descriptors was 28 per molecule. The data was divided into the training and testing data sets without oversampling using *k*-fold cross-validations ($k$ = 4). The overall workflow is shown in Figure 1.

# Results

## Color prediction

Random forest and DBN algorithms were applied for the *in silico* test. Using *k*-fold cross-validations ($k$ = 4), the random forest model identified and utilized the most discriminative features with 100.00% ± 0.0% (mean ± SD) accuracy in the prediction of twelve colors (Figure 2A, C, Figure S1), with a kappa coefficient of 1.0000 ± 0.0000 (mean ± SD). As a type of probability generation model consisting of multiple restricted Boltzmann machines (RBMs), the DBN also performed excellently, with a predictive accuracy of 95.23% ± 0.40% (mean ± SD) (Figure 2B, D) and a kappa coefficient of 0.9400 ± 0.0030 (mean ± SD).

## Key physicochemical features for color perception

Twenty-four descriptors were selected as the key physicochemical features in random forest algorithm with a classification accuracy of 100.00% ± 0.0% by using *k*-fold cross-validations (*k* = 4). The molecular descriptor “B05[F-X]” ranked first, followed by “SddsAs”, “RDF155s” and “F08[O-Si]”. The heatmap of the hierarchical cluster analysis between the twenty-four key features and the twelve colors is shown in Figure 2E. “B10[P-X]”, “B05[P-P]”, “B05[P-Cl]”, “HVcpx”, and “ATS5i” were the main contributors to white, whereas “CATS3D_00_DL” and “E1e” were the most important features in predicting yellow. Information relevant to the key physicochemical features for color perception is reported in Table S1.

**Distinction and connection with olfaction perception**

We next applied the AI platform to predict odor perception based on physicochemical features. In total, 598 structurally diverse molecules were collected and classified into twelve diverse odors based on PubChem [24], including pleasant, unpleasant, ammonia, aromatic, flowery, fruity, spicy, sweet, mild, odorless, characteristic, and other. The accuracies of the odor prediction were 93.40% ± 0.31% for the random forest model using *k*-fold cross-validations (*k* = 4) (Figure 3A, C, Figure S1) and 94.75% ± 0.44% for the DBN (Figure 3B, D), with kappa coefficients of 0.9232 ± 0.0037 and 0.9397 ± 0.0031, respectively. Thirty-nine descriptors were selected as the key physicochemical features in the random forest model with a classification accuracy of 93.40% ± 0.31% (Table S3). The heatmap of the hierarchical cluster analysis between the thirty-nine key physicochemical features and the twelve odors is shown in Figure 3E. Information relevant to the key physicochemical features for

odor perception is presented in Table S2.

To understand the correlation between color and odor, we collected 90 molecules with both color and odor information and analyzed the two groups using a chi-square test. The colors were divided into two categories (white, colorless/other), as were the odors (odorless/other). A correlation was predicted for both types of perception for these molecules ($\chi^2$ = 17.445; $P$ < 0.001). In the complex network of color and odor, key physicochemical features for color and odor prediction were converted into z-scores, and the relationship between each pair of attributes was evaluated by the Pearson correlation coefficient. More than fifty molecular descriptors were found to be interlinked prominently according to their correlation values (the absolute value of the Pearson correlation coefficients ≥ 0.300552) (Figure 4). Three key features "B05[P-CI]", "F08[O-B]" and "CATS3D_14_NL" were shared for both color perception and odor perception.

## Discussion

Clarifying the underlying mechanism of color vision is inherently challenging, as the cognitive process of color vision is multidimensional and includes crossover among the morphology and function of the human visual system [26-28]. Here, we established a framework for distinguishing color without wavelengths based on only 24 physicochemical features. We found that the accuracy and kappa coefficient achieved using random forest (100% ± 0.00%, 1.0000 ± 0.0000) were better than those achieved with the DBN (95.23% ± 0.40%, 0.9400 ± 0.0030) in color prediction

with twelve categories. For odor prediction with twelve categories, the accuracy and kappa coefficient achieved using the DBN (94.75% ± 0.44%, 0.9397 ± 0.0031) were better than those achieved with the random forest (93.40% ± 0.31%, 0.9232 ± 0.0037). Above all, we believe that the machine learning method can be extended to predict both physicochemical properties.

Our findings also suggested that key physicochemical features in distinguishing color and odor are significantly correlated. The 2D Atom Pairs descriptors and many other descriptors interlink at the network between color and odor perception, indicating that both color and odor perceptions are partially determined by the physicochemical properties of the molecules and that color and odor perceptions are closely interrelated. With the prominently interlinked key physicochemical features identified in predicting color and odor, our results tend to call for more evidence for proving the practical relevance between these physicochemical features.

Previous studies on predicting odor have been conducted by the DREAM Olfaction Prediction Challenge [23, 29], with the best Pearson's correlation coefficient achieved around 0.3 between observed and predicted perceptions. A dataset of 476 molecules sensed by 49 voluntary people was applied, and the perceived attributes including the intensity were found to rate differently among the individuals, which considerably complicated the prediction challenge [29]. The winning algorithm of the DREAM challenge indicated that the random forest outperforms other base learners (linear, ridge, and support vector machine) in predicting odor [29]. Our study collected a total of 598 structurally diverse molecules and classified them into twelve diverse odors

based on PubChem to avoid a subjective effect on odor perception. We added the DBN method and achieved the best result in odor prediction with a classification accuracy of 94.75% ± 0.44% for 12 categories using all features. In contrast, the performance of DBN using the key physicochemical features was less than satisfaction for the following reasons. Firstly, the key physicochemical features identified in predicting color and odor were selected by random forest and genetic algorithm. These features may not be suitable for DBN,which can map the raw data to low-dimensional space by unsupervised learning in the pre-training phase. Secondly, although DBN is a multi-layered recurrent neural network trained with energy minimizing methods, the network structure of DBN has a great influence on the learning performance. The network structure of DBN utilizing all features and the key physicochemical features are exactly the same in our study. If other algorithm like the Particle Swarm Optimization Algorithm could be used to optimize the number of DBN hidden-layer nodes, the performance of DBN network may improve.

In addition, odor sensing was found to be less accurate than that of color. Several factors may affect the accuracy of the AI in odor perception. First, odor perception is more subjective based on perceived biases, and it is challenging to confirm the number and character of its perceptual dimensions [30]. Defining a specific odor is especially difficult for human beings compared with other sensory modalities [31]. Second, the olfactory system involves high-dimensional input with attached arbitrary associations, whereas color vision occurs under predefined spatial conditions [12]. Thus, the processing demands of the two systems are not entirely consistent with each

other. Third, the two systems employ different strategies in temporal coding to convey information. The olfactory system uses temporal coding to increase its representational capacity, while the visual system uses temporal coding to reduce the redundancy [12].

In this study, we add new insight into the decoding of color vision, but the controlling and tuning of these codes require further investigation. Inspired by the key physicochemical features involved in color prediction, researchers may be able to develop materials with vivid colors for potential applications in sensing technologies, security, light-emitting sources, and paints [32-34].

## Potential implications

The ability to explain visual neural activities from the perspective of AI would also enable us to build an artificial vision system that could favorably stimulate the color vision of an individual. Once the perception process of human color vision is completely decoded, the AI platform may help in the design of artificial brain stimulation interfaces that can restore color vision and enable blind patients to “see” colors without biological eyes.

## Methods

### Random forest algorithm

Random forest is an ensemble learning method for regression and classification [35]. In a random forest model, each decision tree is built from a random sampling of samples and features, which can deliver generalized knowledge [35]. Furthermore, a

random set of features is used to determine the best split at each node during the construction of a tree. Here, the dimensionality of the physicochemical data was high, with 5270 descriptors per molecule, and the perception data matrix was sparse. By averaging hundreds of trees in this work, the effects of outliers and noise were reduced. The random forest parameter mTry (i.e., the number of input variables randomly chosen at each split) was set to 72 (square root of 5270 features), while the other random forest parameter nTree (i.e., the number of trees to grow for each forest) was set to 100. $k$-fold cross-validation ($k$ = 4) was applied for the classification.

**Deep belief network (DBN)**

DBN is a type of probability generative model that consists of multiple RBMs. The superposition of multiple RBMs solves the training problem of multiple layered neural networks. The overall training process of the DBN includes two stages: a pretraining stage and a fine-tuning stage [36]. 1) Pretraining stage: Each RBM includes a visual layer and a hidden layer. There are no interlayer connections between the visual layer and hidden layer. After training the first RBM, the activation value of the hidden layer of the first RBM is input into the visual layer of the second RBM. 2) Fine-tuning stage: With the help of the BP neural network that resides after the last RBM and the chain rule of derivation, the DBN will be trained as a whole neural network. In this study, the input of the DBN is the vector consisting of 5270 molecular descriptors. During the first stage of the DBN, the dimensions of the vector are compressed. During the second stage, the compressed vector can be used for classification.

We compared three DBN structures for the prediction of either color or odor, and optimizations of the parameters of each structure were conducted. The architecture that performed best in both color and odor prediction was the input layer with 5270 neurons and only one RBM with 5270 visible neurons and 500 hidden neurons. The moderate performance was achieved with the input layer with 5270 neurons and two RBMs. One RBM was composed of 5270 visible neurons and 2000 hidden neurons, and the other contained 2000 visible neurons and 500 hidden neurons. The worst performance was achieved with the input layer with 5270 neurons and three RBMs. One RBM contained 5270 visible neurons and 2000 hidden neurons, one was composed of 2000 visible neurons and 1000 hidden neurons, and the last contained 1000 visible neurons and 500 hidden neurons. Therefore, the best architecture was used in the follow-up prediction.

**Feature selection**

Random forest algorithm and genetic algorithm [37,38] were both applied to select the key features in this study. The random forest algorithm enable us to estimate the importance of each molecular descriptor by permuting the values of the descriptors across samples and computing the increases in prediction errors. The samples left-out in the training of each classifier (referred to as out-of-bag samples) are used for feature selection by determining the importance of different features during classification process. A value of "0" signifies that the feature corresponding to this bit is not needed for the classification; otherwise, the feature is needed for the classification. A total of 1601 features were recognized as needed for the classification

of color, and 1820 were recognized as needed for the classification of odor in random forest algorithm. In order to compare with the selection results by using both the genetic algorithm and random forest algorithm, the numbers of the key features selected were similar.

Genetic algorithms designed for feature selection can implement feature selection and classification processes simultaneously [39]. The accuracy of the random forest was adopted as the fitness evaluation function of the genetic algorithm. The chromosome coding method was binary coding, and the length of the chromosome was equal to the dimension of the feature vector. Because of the randomness of the genetic algorithm, the experiment was conducted 20 times. After running the genetic feature selection task 20 times, 24 descriptors were selected 18 times for color, 39 descriptors were selected 16 times for odor.

**Feature ranking**

Feature ranking for random forest algorithm used out of bag permutation error. With the features selected from the genetic algorithm, feature ranking was performed to study which attributes were more important for classification. In this process, for a feature $A_i$ in the feature set $\{A_1, A_2 \ldots A_n\}$, the validating accuracy for the original validation dataset is *acc1*. The validatiom accuracy obtained with the random permutation of $A_i$ is *acc2*. |*acc2-acc1*| is an indicator used to measure the importance of $A_i$. Then, all features are compared with this indicator. Because of the randomness of the random forest, this process was conducted 20 times.

### Hierarchical clustering

Hierarchical approaches have the ability to simultaneously uncover multiple layers of a clustering structure [40]. The R heatmap package was used for clustering in this study.

### Statistical analysis

The data were collected using the Qualtrics Web-based questionnaire package and analyzed using IBM SPSS Statistics version 24 (SPSS, RRID:SCR_002865).

## Availability of supporting source code and requirements

Project name: Color Odor Prediction

Project home page: https://github.com/Hugo0512/ColorOdorprediction

Operating system: Platform independent

Programming language: MATLAB

License: MIT

## Availability of Supporting Data and Materials

All methods were implemented with MATLAB R2016a (MATLAB, RRID:SCR_001622) on a HP Z420 workstation with Intel Xeon CPU E5-1620 v2@ 3.70GHZ and 16GB RAM. The operating system was Windows 7. Data corresponding to the molecules used in this study are presented in Supplementary Data1-3, and archives of all the code and all supporting data is available in the *GigaScience* GigaDB repository[41].

## Additional Files

Table S1. Attribute importance ranking of color.

Table S2. Attribute importance ranking of odor.

Table S3. The results for each fold in the 4-fold cross-validation.

Figure S1. The prediction accuracies of random forest models for twelve colors and twelve odors using all features.

Supplementary Data1. The datasets of the 1267 structurally diverse molecules labeled with 12 diverse colors and 5270 molecular descriptors.

Supplementary Data2. The datasets of the 598 structurally diverse molecules labeled with 12 diverse odors and 5270 molecular descriptors.

Supplementary Data3. The datasets of the 90 molecules with both color and odor information.

## Abbreviations

3D, Three dimensional; AI, Artificial intelligence; DBN, Deep belief network; Dragon, Software for the calculation of molecular descriptors; GETAWAY, Geometry, topology and atom-weights assembly; RBM, Restricted Boltzmann machine.

## Completing interests

The authors declare that they have no competing interests.

## Funding

This study was funded by the National Key R&D Program of China (2018YFC0116500), the Key Research and Development Program of Guangdong

Province (No. 2018B010109008), the National Natural Science Foundation of China (81770967, 81822010). The funders had no role in the study design, data collection, and analysis, the decision to publish or the preparation of the manuscript.

## Author contributions

H.T.L., X.Y.Z. and D.R.L. conceived and designed the prediction algorithm, X.Y.Z., K.Z., D.R.L. and L.H. were responsible for data management and performing the computational analyses. R.X.W., Z.Z.L., X.H.W., and E.P.L. analyzed the discriminative features and prepared the figures. H.T.L., X.Y.Z. and D.R.L. contributed to the writing of the manuscript. Z.Y., C.C., X.S.G., K.X.C., K.H., X.Y.L., and Z.Q.H. contributed to the critical review of the study, and all authors read and approved the final manuscript.

## Acknowledgments

We thank Xiaoming Chen (School Of Chemistry, Sun Yat-sen University) for reading, discussing and providing constructive comments for the manuscript.

## References

1. Pete Vukusic & J. Roy Sambles. Photonic structures in biology. *Nature* 2003; 424, 852–855.

2. Le Chang, Pinglei Bao & Doris Y. Tsao. The representation of colored objects in macaque color patches. *Nature Communications* 2017; 8 (1).

3. S Kinoshita, S Yoshioka & J Miyazaki. Physics of structural colors. *Rep. Prog. Phys*; 2008, 71, 30pp.

4. Wilkinson, F.A. & Murillo, S.G. Advanced inorganic chemistry. 1988. Wiley.

5. McMurry, John. Organic chemistry. 2007. Brooks Cole.

6. Hallenbeck. Recent Advances in QSAR Studies. *Challenges & Advances in Computational Chemistry & Physics* 2010; 8, 31-32.

7. Paul, A. The use of nanocrystals in biological detection. *Nat Biotechnol* 2004; 22, 47-52.

8. Chen, F. & Gerion, D. Fluorescent CdSe/ZnS Nanocrystal−Peptide Conjugates for Long-term, Nontoxic Imaging and Nuclear Targeting in Living Cells. *Office of Scientific & Technical Information Technical Reports* 2004; 4, 1827-1832.

9. Rossiter, K.J. Structure−Odor Relationships. *Chemical ReviewsChemical ReviewsChem. Rev.* 1996; 96, 3201-3240.

10. Turin, L. A method for the calculation of odor character from molecular structure. *J Theor Biol* 2002; 216, 367-385.

11. Czerny, M., Brueckner, R., Kirchhoff, E., Schmitt, R. & Buettner, A. The influence of molecular structure on odor qualities and odor detection thresholds of volatile alkylated phenols. *Chem Senses* 2011; 36, 539.

12. Gire, D.H., *et al*. Temporal processing in the olfactory system: can we see a smell. *Neuron* 2013; 78, 416-432.

13. Yoshida, M., Itoh, Y., Ômura, H., Arikawa, K. & Kinoshita, M. Plant scents modify innate color preference in foraging swallowtail butterflies. *Biol Lett* 2015; 11.

14. Morrot, G., Brochet, F. & Dubourdieu, D. The Color of Odors. *Brain & Language* 2001; 79, 309-320.

15. Zellner, D.A. & Kautz, M.A. Color affects perceived odor intensity. *J Exp Psychol Hum Percept Perform* 1990; 16, 391-397.

16. Dubose, C.N., Cardello, A.V. & Maller, O. Effects of colorants and flavorants on identification, perceived flavor and hedonic quality of fruit-flavored beverages and cake. *J Food Sci* 2010; 45, 1393-1399.

17. Royet, J.P., *et al.* Functional anatomy of perceptual and semantic processing for odors. *J Cogn Neurosci* 1999; 11, 94-109.

18. Jadauji, J.B., *et al.* Modulation of olfactory perception by visual cortex stimulation. *Journal of Neuroscience the Official Journal of the Society for Neuroscience* 2012; 32, 3095.

19. Gershman, S.J., Horvitz, E.J. & Tenenbaum, J.B. Computational rationality: A converging paradigm for intelligence in brains, minds, and machines. *Science* 2015; 349, 273-278.

20. Sanchez-Lengeling, B. & Aspuru-Guzik, A. Inverse molecular design using machine learning: Generative models for matter engineering. *Science* 2018; 361, 360-365.

21. Butler, K.T., Davies, D.W., Cartwright, H., Isayev, O. & Walsh, A. Machine learning for molecular and materials science. *Nature* 2018; 559, 547-555.

22. Paruzzo, F.M., *et al.* Chemical shifts in molecular solids by machine learning. *Nat Commun* 2018; 9, 4501.

23. Keller, A., *et al.* Predicting human olfactory perception from chemical features of odor molecules. *Science* 2017; 355, 820-826.

24. Kim, S., *et al.* PubChem Substance and Compound databases. *Nucleic Acids Res* 2016; 44, D1202-1213.

25. Todeschini, R. & Consonni, V. Handbook of Molecular Descriptors. 2000.

26. Solomon, S.G. & Lennie, P. The machinery of colour vision. *Nat Rev Neurosci* 2007; 8,

276-286.

27. Bennett, A. .D. & Théry, M. Avian Color Vision and Coloration: Multidisciplinary Evolutionary Biology. *Am Nat* 2007; 169, S1-1S6.

28. Kelber, A. & Osorio, D. From spectral information to animal colour vision: experiments and concepts. *Proceedings: Biological Sciences* 2010; 277, 1617-1625.

29. Hongyang Li, Bharat Panwar, Gilbert S. Omenn & Yuanfang Guan. Accurate prediction of personalized olfactory perception from large-scale chemoinformatic features. *GigaScience* 2017; 7, 1–11.

30. Kaeppler, K. & Mueller, F. Odor classification: a review of factors influencing perception-based odor arrangements. *Chem Senses* 2013; 38, 189-209.

31. Wippich, W., Mecklenbräuker, S. & Trouet, J. Implicit and explicit memories of odors. *Archiv Für Psychologie* 1989; 141, 195.

32. Hwang, J., *et al.* Electro-tunable optical diode based on photonic bandgap liquid-crystal heterojunctions. *Nat Mater* 2005; 4, 383-387.

33. Lee, H.S., Shim, T.S., Hwang, H., Yang, S.M. & Kim, S.H. Colloidal Photonic Crystals toward Structural Color Palettes for Security Materials. *Chemistry of Materials* 2013; 25, 2684-2690.

34. Sung Yeun, C., *et al.* Mesoporous bragg stack color tunable sensors. *Nano Lett* 2006; 6, 2456-2461.

35. Breiman, L. Random Forests. *Mach Learn* 2001; 45, 5-32.

36. Le, R.N. & Bengio, Y. Representational power of restricted boltzmann machines and deep belief networks. *Neural Comput* 2008; 20, 1631-1649.

37. Wang L, et al. Comparative analysis of image classification methods for automatic diagnosis of ophthalmic images. *Scientific reports* 2017; 7, 41545.

38. Zhang K, et al. Systemically modeling the relationship between climate change and wheat aphid abundance. *Science of The Total Environment* 2019; 674, 392-400.

39. Zhang K, et al. Prediction of postoperative complications of pediatric cataract patients using data mining. *J Transl Med* 2019; 3,17(1).

40. Eisen, M.B., *et al.* Cluster analysis and display of genome-wide expression patterns. *Proceedings of the National Academy of Sciences* 1998; 14863-14868.

41. Zhang X; Zhang K; Lin D; Zhu Y; Chen C; He L; Guo X; Chen K; Wang R; Liu Z; Wu X; Long E; Huang K; He Z; Liu X; Lin H (2020): Supporting data for "Artificial intelligence deciphers codes for color and odor perceptions based on large-scale chemoinformatic data" GigaScience Database. http://dx.doi.org/10.5524/100700

# Figure legends

**Figure 1. The overall workflow of color prediction and odor prediction.** 1267 structurally diverse molecules were labeled with 12 diverse colors, and 598 structurally diverse molecules were labeled with 12 diverse odors. In addition, 5270 physicochemical features of each molecule were generated by Dragon. Random forest models and deep belief networks were built to predict colors or odors using their physicochemical features. Feature selection were conducted by random forest models and the genetic algorithm. With the selected feature, random forest models and deep belief networks were reused for color and odor prediction. The models were evaluated based on the means and variances of the accuracies between the labeled and predicted colors or odors.

**Figure 2. Color prediction using the random forest model and DBN.** A. The confusion matrix for the classification of color with 100.00% accuracy by the random forest. The X-axis presents the labeled colors of the molecules, and the Y-axis presents the predicted colors of the molecules. B. The classification results for color were as high as 95.23% using the DBN. The *X*-axis presents the learning rate, the *Y*-axis presents the algorithm parameter "momentum", and the *Z*-axis presents the accuracy rate. C. The boxplot presenting the accuracy of color prediction using the random forest with all features, the top 24 features selected by random forest models, the top 24 features selected by random forest and the genetic algorithm and the total 48 features from above. The median values of these boxplots are labeled. D. The boxplot presenting the accuracy of color prediction using the DBN with all features,

the top 24 features selected by random forest models, the top 24 features selected by random forest and the genetic algorithm and the total 48 features from above.The median values of these boxplots are labeled. #Random forest models, *Random forest models and genetic algorithm. E. The heatmap of the the correlation values between the top 24 features selected by random forest models and the twelve colors based on the hierarchical clustering framework. The connections between the colors and descriptors were calculated by the Euclid distances.

**Figure 3. Odor prediction using the random forest model and DBN.** A. The confusion matrix for the classification of odor with 93.40% accuracy by the random forest. B. The classification results for odor were as high as 94.75% using the DBN. The *X*-axis presents the learning rate, the *Y*-axis presents the algorithm parameter "momentum", and the *Z*-axis presents the accuracy rate. C. The boxplot to present the accuracy of color prediction using the random forest with all features, the top 39 features selected by random forest models, the top 39 features selected by the random forest and the genetic algorithm and the total 78 features from above. The median values of these boxplots are labeled. D. The boxplot presenting the accuracy of color prediction using the DBN with all features, the top 39 features selected by random forest models, the top 39 features selected by random forest and the genetic algorithm and the total 78 features from above. The median values of these boxplots are labeled. #Random forest models, *Random forest models and genetic algorithm. E. The heatmap of the correlation values between the top 39 features selected by random forest models and the twelve odors based on the hierarchical clustering framework.

Connections between the odors and descriptors were calculated by the Euclid distances.

**Figure 4. The correlations between color and olfaction perception.** A. Of the 1267 molecules with color, 90 also had odor information. B. Schematic diagram of the key physicochemical features for color and odor perceptions in the interactome. The key features for color perception were closely connected with the key features for odor perception. The distance of each line represents its correlation value.

## Supplementary Materials

**Table S1. Attribute importance ranking of color.**

| Ranking | Descriptor Name | Description | Block |
|---|---|---|---|
| 1 | B05[F-X] | Presence/absence of F - X at topological distance 5 | 2D Atom Pairs |
| 2 | SddsAs | Sum of ddsAs E-states | Atom-type E-state indices |
| 3 | RDF155s | Radial Distribution Function - 155 / weighted by I-state | RDF descriptors |
| 4 | F08[O-Si] | Frequency of O - Si at topological distance 8 | 2D Atom Pairs |
| 5 | ATS5i | Broto-Moreau autocorrelation of lag 5 (log function) weighted by ionization potential | 2D autocorrelations |
| 6 | CATS3D_14_NL | CATS3D Negative-Lipophilic BIN 14 (14.000 - 15.000 Å) | CATS 3D |
| 7 | F02[O-I] | Frequency of O - I at topological distance 2 | 2D Atom Pairs |
| 8 | SpMax4_Bh(s) | Largest eigenvalue n. 4 of Burden matrix weighted by I-state | Burden eigenvalues |
| 9 | F01[Br-Si] | Frequency of Br - Si at topological distance 1 | 2D Atom Pairs |
| 10 | ATSC3e | Centred Broto-Moreau autocorrelation of lag 3 weighted by Sanderson electronegativity | 2D autocorrelations |
| 11 | E1e | 1st component accessibility directional WHIM index / weighted by Sanderson electronegativity | WHIM descriptors |
| 12 | Mor27i | Signal 27 / weighted by ionization potential | 3D-MoRSE descriptors |
| 13 | HVcpx | Graph vertex complexity index | Information indices |
| 14 | C-014 | CX4 | Atom-centred fragments |
| 15 | SpPosA_Dz(p) | Normalized spectral positive sum from Barysz matrix weighted by polarizability | 2D matrix-based descriptors |
| 16 | nN | Number of Nitrogen atoms | Constitutional indices |
| 17 | F03[N-P] | Frequency of N - P at topological distance 3 | 2D Atom Pairs |
| 18 | CATS3D_00_DL | CATS3D Donor-Lipophilic BIN 00 (0.000 - 1.000 Å) | CATS 3D |
| 19 | VE1_B(m) | Coefficient sum of the last eigenvector (absolute values) from Burden matrix weighted by mass | 2D matrix-based descriptors |
| 20 | B08[C-Br] | Presence/absence of C - Br at topological distance 8 | 2D Atom Pairs |

| | | | |
|---|---|---|---|
| 21 | F08[O-B] | Frequency of O - B at topological distance 8 | 2D Atom Pairs |
| 22 | B10[P-X] | Presence/absence of P - X at topological distance 10 | 2D Atom Pairs |
| 23 | B05[P-P] | Presence/absence of P - P at topological distance 5 | 2D Atom Pairs |
| 24 | B05[P-Cl] | Presence/absence of P - Cl at topological distance 5 | 2D Atom Pairs |

**Table S2. Attribute importance ranking of odor.**

| Ranking | Descriptor Name | Description | Block |
|---|---|---|---|
| 1 | B09[O-F] | Presence/absence of O - F at topological distance 9 | 2D Atom Pairs |
| 2 | Eig12_AEA(dm) | Eigenvalue n. 12 from augmented edge adjacency mat. weighted by dipole moment | Edge adjacency indices |
| 3 | DISPm | Displacement value / weighted by mass | Geometrical descriptors |
| 4 | F09[B-Si] | Frequency of B - Si at topological distance 9 | 2D Atom Pairs |
| 5 | CATS3D_10_NN | CATS3D Negative-Negative BIN 10 (10.000 - 11.000 Å) | CATS 3D |
| 6 | H8i | H autocorrelation of lag 8 / weighted by ionization potential | GETAWAY descriptors |
| 7 | F04[S-Br] | Frequency of S - Br at topological distance 4 | 2D Atom Pairs |
| 8 | J_B(i) | Balaban-like index from Burden matrix weighted by ionization potential | 2D matrix-based descriptors |
| 9 | CATS3D_07_PL | CATS3D Positive-Lipophilic BIN 07 (7.000 - 8.000 Å) | CATS 3D |
| 10 | P_VSA_ppp_A | P_VSA-like on potential pharmacophore points, A - hydrogen-bond acceptor | P_VSA-like descriptor |
| 11 | F04[N-Cl] | Frequency of N - Cl at topological distance 4 | 2D Atom Pairs |
| 12 | VE1_Dt | Coefficient sum of the last eigenvector (absolute values) from detour matrix | 2D matrix-based descriptors |
| 13 | F10[N-S] | Frequency of N - S at topological distance 10 | 2D Atom Pairs |
| 14 | F06[S-S] | Frequency of S - S at topological distance 6 | 2D Atom Pairs |
| 15 | D/Dtr08 | Distance/detour ring index of order 8 | Ring descriptors |
| 16 | DLS_02 | Modified drug-like score from Oprea et al. (6 rules) | Drug-like indices |
| 17 | B10[P-Si] | Presence/absence of P - Si at topological distance 10 | 2D Atom Pairs |
| 18 | NddssSe | Number of atoms of type ddssSe | Atom-type E-state indices |
| 19 | SpAD_D | Spectral absolute deviation from topological distance matrix | 2D matrix-based descriptors |
| 20 | L3m | 3rd component size directional WHIM index / weighted by mass | WHIM descriptors |
| 21 | F10[C-Cl] | Frequency of C - Cl at topological distance 10 | 2D Atom Pairs |

| | | | |
|---|---|---|---|
| 22 | Chi_Dt | Randic-like index from detour matrix | 2D matrix-based descriptors |
| 23 | RDF085v | Radial Distribution Function - 085 / weighted by van der Waals volume | RDF descriptors |
| 24 | VR3_Dz(Z) | Logarithmic Randic-like eigenvector-based index from Barysz matrix weighted by atomic number | 2D matrix-based descriptors |
| 25 | HyWi_B(i) | Hyper-Wiener-like index (log function) from Burden matrix weighted by ionization potential | 2D matrix-based descriptors |
| 26 | B05[P-Cl] | Presence/absence of P - Cl at topological distance 5 | 2D Atom Pairs |
| 27 | CATS3D_03_LL | CATS3D Lipophilic-Lipophilic BIN 03 (3.000 - 4.000 Å) | CATS 3D |
| 28 | HATS7i | Leverage-weighted autocorrelation of lag 7 / weighted by ionization potential | GETAWAY descriptors |
| 29 | ZM1 | First Zagreb index | Topological indices |
| 30 | HATS6m | Leverage-weighted autocorrelation of lag 6 / weighted by mass | GETAWAY descriptors |
| 31 | Eig11_AEA(dm) | Eigenvalue n. 11 from augmented edge adjacency mat. weighted by dipole moment | Edge adjacency indices |
| 32 | CATS3D_14_NL | CATS3D Negative-Lipophilic BIN 14 (14.000 - 15.000 Å) | CATS 3D |
| 33 | nSO3OH | Number of sulfuric (thio-/dithio-) acids | Functional group counts |
| 34 | TDB04u | 3D Topological distance based descriptors - lag 4 unweighted | 3D autocorrelations |
| 35 | F10[P-Br] | Frequency of P - Br at topological distance 10 | 2D Atom Pairs |
| 36 | F08[O-B] | Frequency of O - B at topological distance 8 | 2D Atom Pairs |
| 37 | JGI4 | Mean topological charge index of order 4 | 2D autocorrelations |
| 38 | P_VSA_MR_8 | P_VSA-like on Molar Refractivity, bin 8 | P_VSA-like descriptor |
| 39 | CATS3D_09_NN | CATS3D Negative-Negative BIN 09 (9.000 - 10.000 Å) | CATS 3D |

**Table S3. The results for each fold in the 4-fold cross-validation.**

| Task | Method | Number of features | Mean accuracy | Accuracy (4-fold cross-validation) |
|---|---|---|---|---|
| **Color perception** | Random forest | ① All features | 100% | 100%, 100%, 100%, 100% |
| | | ② 24 features [#] | 100% | 100%, 100%, 100%, 100% |
| | | ③ 24 features [*] | 99.45% | 99.37%, 99.69%, 99.36%, 99.37% |
| | | ② + ③features | 100% | 100%, 100%, 100%, 100% |
| | DBN | ① All features | 95.23% | 95.89%, 94.93%, 94.88%, 95.23% |
| | | ② 24 features [#] | 47.35% | 47.95%, 49.06%, 46.50%, 45.89% |
| | | ③ 24 features [*] | 44.20% | 44.16%, 43.75%, 44.59%, 44.30% |
| | | ② + ③features | 48.79% | 53.63%, 44.69%, 50.00%, 46.84% |
| **Odor perception** | Random forest | ① All features | 93.40% | 93.33%, 93.92%, 93.20%, 93.15% |
| | | ② 39 features [#] | 93.40% | 93.33%, 93.92%, 93.20%, 93.15% |
| | | ③ 39 features [*] | 93.38% | 93.33%, 93.90%, 93.18%, 93.11% |
| | | ② + ③features | 93.40% | 93.33%, 93.92%, 93.20%, 93.15% |
| | DBN | ① All features | 94.75% | 95.24%, 94.35%, 94.27%, 95.13% |
| | | ② 39 features [#] | 36.18% | 43.33%, 30.41%, 36.73%, 34.25% |
| | | ③ 39 features [*] | 31.46% | 31.29%, 31.33%, 29.00%, 34.25% |
| | | ② + ③features | 42.44% | 48.00%, 38.51%, 48.30%, 34.93% |

[#] Random forest models

[*] Random forest models and genetic algorithm

**Supplementary Data1.** The datasets of the 1267 structurally diverse molecules labeled with 12 diverse colors and 5270 molecular descriptors.

**Supplementary Data2.** The datasets of the 598 structurally diverse molecules labeled with 12 diverse odors and 5270 molecular descriptors.

**Supplementary Data3.** The datasets of the 90 molecules with both color and odor information.

Figure
Click here to access/download;Figu

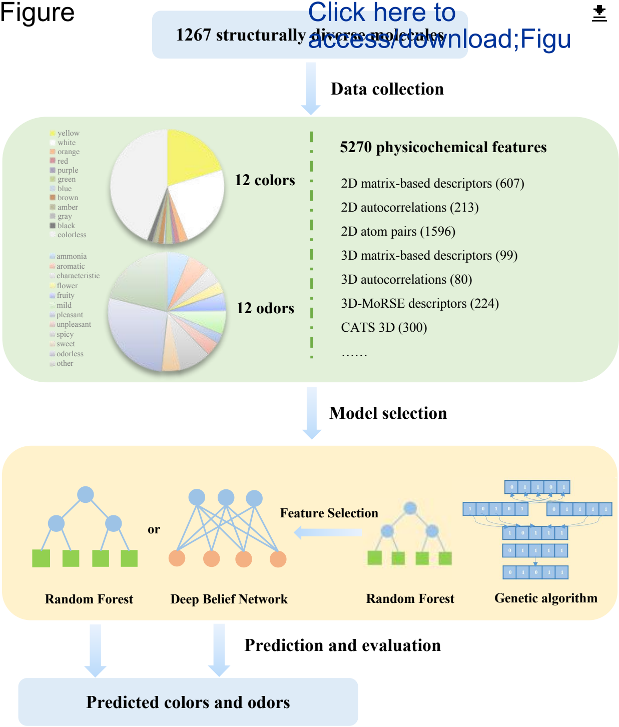

Figure

Click here to access/download;Figu

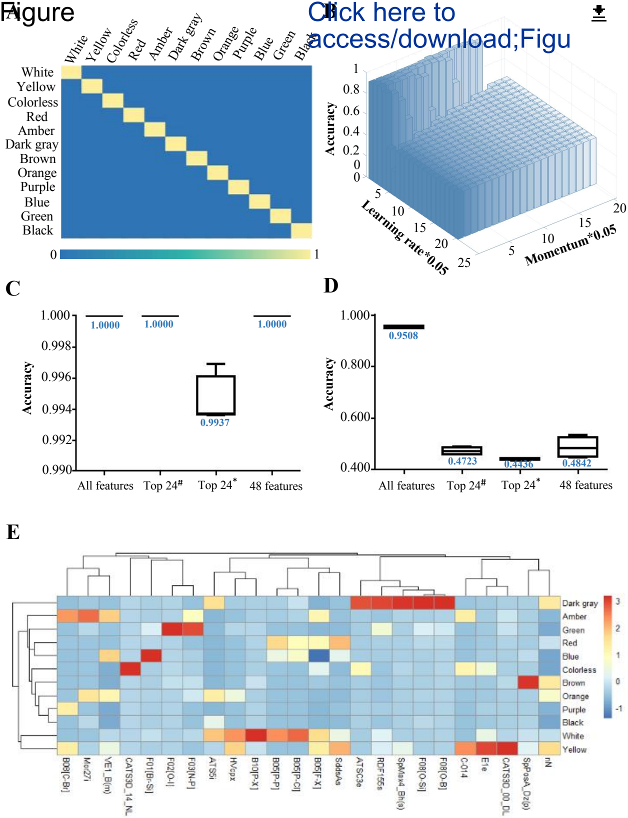

Figure
Click here to access/download;Figure

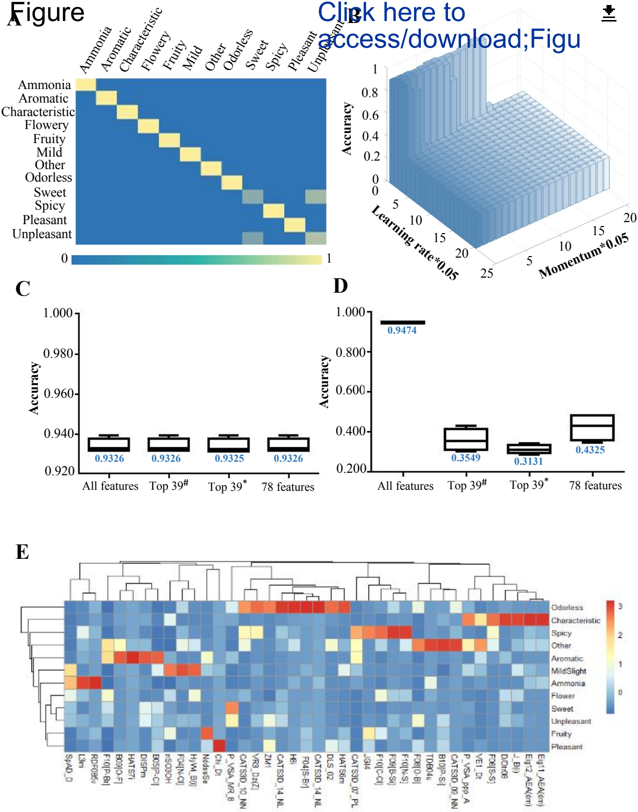

Figure

Click here to access/download;Figu

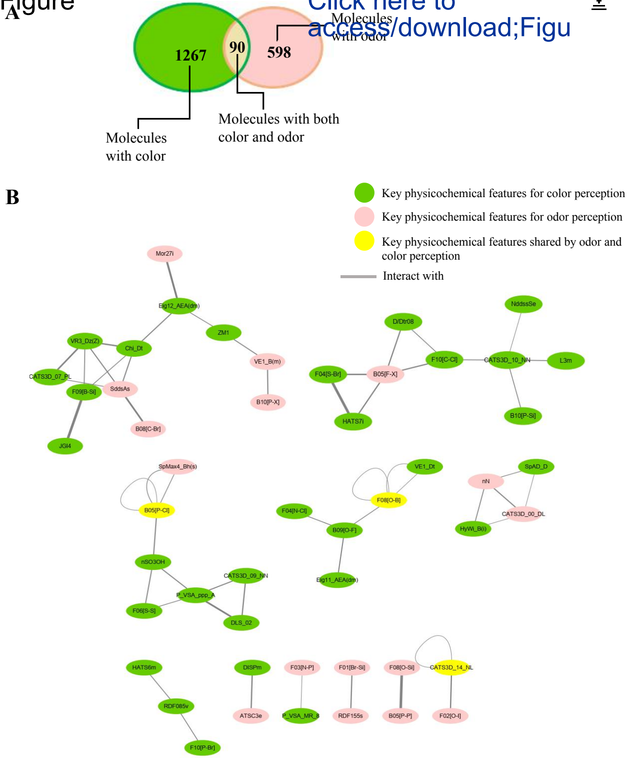

Figure

Click here to access/download;Figu

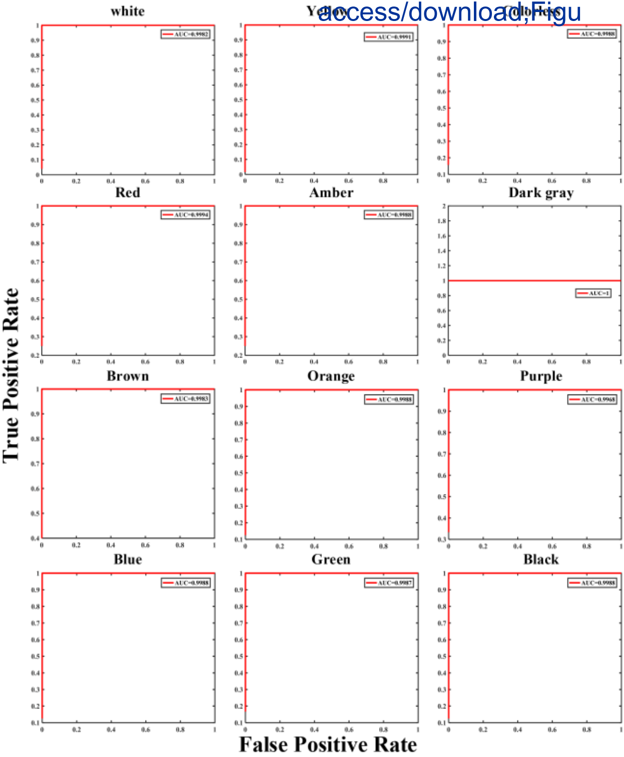

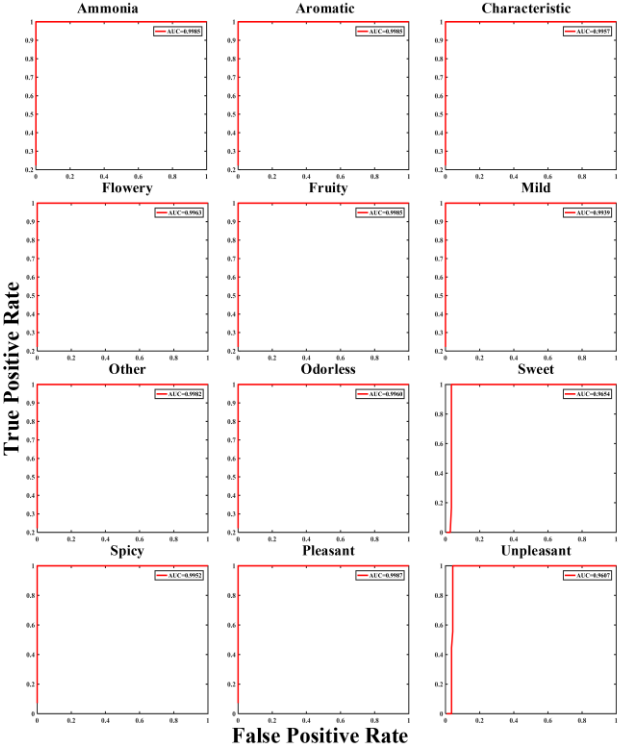

Ammonia
AUC=0.9985
Aromatic
AUC=0.9985
Characteristic
AUC=0.9957
Flowery
AUC=0.9963
Fruity
AUC=0.9985
Mild
AUC=0.9939
Other
AUC=0.9982
Odorless
AUC=0.9960
Sweet
AUC=0.9654
Spicy
AUC=0.9952
Pleasant
AUC=0.9987
Unpleasant
AUC=0.9607
True Positive Rate
False Positive Rate

Click here to access/download
**Supplementary Material**
Supplementary Data1.xlsx

Click here to access/download
**Supplementary Material**
Supplementary Data2.xlsx

Click here to access/download
**Supplementary Material**
Supplementary Data3.xlsx

Response to Reviewers Click here to access/download;Personal Cover;Response to Reviewers.docx

**'Response to Reviewers' files GIGA-D-19-00112R2**

**Artificial intelligence deciphers codes for color and odor perceptions based on large-scale chemoinformatic data**

***GigaScience***

Dear Edmunds and Reviewers,

Thank you so much for your agreement and clear corrections on our manuscript.

The following are our point-by-point responses to the reviewer's comments and corresponding changes are marked in the revised manuscript. We hope that we have addressed your concern adequately.

---------------------------------

**Our point-by-point responses are as follows:**

**Reviewer #2:** ***Comment (1):*** *Thank you for the second revision. It is quite impressive to see prediction accuracies of random forest models for predicting individual colours and odours. It is clear from your results (Table S3) that DBN might choose different sets of features in order to perform better because DBN can reach to 95% accuracy but fails to deliver best performance for 24 features chosen by random forest/genetic algorithm. Can you please make a short discussion on this point in your manuscript. This will reflect on the poor performance of DBN.*

**Response:** Thanks so much for your insightful suggestions on our manuscript all along.

We considered that there were two main reasons why DBN failed to deliver best performance with the 24 features. Firstly, the key physicochemical features identified in predicting color and odor were selected by random forest and genetic algorithm. These features may not be suitable for DBN,which can map the raw data to low-dimensional space by unsupervised learning in the pre-training phase. Secondly, although DBN is a multi-layered recurrent neural network trained with energy minimizing methods, the network structure of DBN has a great influence on the learning performance. The network structure of DBN utilizing all features

and the key physicochemical features are exactly the same in our study. If other algorithm like the Particle Swarm Optimization Algorithm could be used to optimize the number of DBN hidden-layer nodes, the performance of DBN network may improve. We have further discussed these potential reasons for the performance of DBN in Line 211-222.

***Comment (2):*** *line 115. specify the supplementary data table number.*

**Response:** Thank you for your scrupulous correction. "Supplementary Data1-3" has been added in Line 114.

***Comment (3):*** *line 145-148 is not clear. As you stated the sentence saying random forest algorithm and the combination of the genetic algorithm and random forest algorithm..., where the second part of the sentence explains the advantage of random forest. This leaves the first part incomplete.*

**Response:** We really appreciate your constructive suggestions. To highlight the best classification accuracy with 24 key physicochemical features selected by random forest in the results section, we have removed the description of feature selection methods here and revised the sentence in the method section (Line 288-290).

***Comment (4):*** *line 195-196. the sentence is misleading to suggest that physicochemical features are connected. This needs more clarity.*

**Response:** Many thanks for your critical suggestion. We modified this sentence to "Our findings also suggested that key physicochemical features in distinguishing color and odor are significantly correlated" (Line 192-193).

***Comment (5):*** *line 199-202, this sentence is also misleading because the outcome of the edges between the two features is not explained properly. One can see that due to the correlation there are two features connected but there is no evidence suggesting that they influence changing the odour and colour.*

**Response:** Thanks for your suggestion. We have changed the statement in a more conservative manner (Line 197-199).

***Comment (6):*** *There are some places where you mention random forest algorithm and the combination of the genetic algorithm and random forest algorithm. this is very confusing. I think you want to say that for feature selection random forest and genetic algorithm were used. Can you please make this consistent.*

**Response:** Thank you again for your helpful corrections. We have made modifications to unify the descriptions for feature selection (Line 91, Line 287, Line 454, Line 467).

-------------------------------------------------------

**Finally, thank you again for all of the helpful comments, and we hope that you will now find our revisions suitable for publication.**

Sincerely yours,

Haotian Lin on behalf of all authors

Revision Cover Letter
Click here to access/download;Personal Cover;Revision Cover Letter.doc

SUN YAT-SEN UNIVERSITY

Haotian Lin, M.D., Ph.D.
State Key Laboratory of Ophthalmology
Zhongshan Ophthalmic Center
Sun Yat-sen University
Tel: +86-13802793086
E-mail: haot.lin@hotmail.com

Jan 7, 2020

Dear Edmunds,

Thank you so much for your comments on our manuscript entitled "Artificial intelligence deciphers codes for color and odor perceptions based on large-scale chemoinformatic data". We sincerely treasure all the constructive and crucial suggestions from you and reviewers. Based on the provided suggestions and comments, we have addressed the issues carefully and made necessary corrections accordingly. We hope that the revised manuscript can convince you and reviewers.

----------------------------------

**The main improvements and revisions are as follows:**

The discussion section was improved by explaining potential reasons for the performance of the deep belief network (Line 211-222), and the descriptions for feature selection have been unified (Line 91, Line 287, Line 454, Line 467).

----------------------------------

Thank you once again for your agreement and help with our research.

Sincerely yours,

Haotian Lin, on behalf of all authors
