# Supplementary material for: Artificial intelligence deciphers codes for color and odor perceptions based on large-scale chemoinformatic data
Source: Gigascience. 2020 Feb 26;9(2):giaa011. doi: 10.1093/gigascience/giaa011 (PMC7043059; doi:10.1093/gigascience/giaa011)
Supplement: giaa011_Response_to_Reviewer_Comments_Original_Submission [file giaa011_response_to_reviewer_comments_original_submission.pdf]

Author's Response To Reviewer Comments

Close

Dear Scott Edmunds and Reviewers,

Thank you so much for the agreements and insightful suggestions on our manuscript. The following are our point-by-point responses to the reviewers' comments and corresponding changes are marked in the revised manuscript. We hope that we have addressed all the suggestions adequately. Please let us know if you have any further questions or suggestions.

Our point-by-point responses are as follows:

Reviewer #1:

Comment (1): The authors proposed machine learning approaches for predicting color and odor of a small molecule based on large-scale chemoinformatic features. They investigated the interplay between color and odor perception and found chemoinformatic features in predicting color and odor perception. Key results and information are missing in this manuscript. None of the figure legends were provided. For example, in line 118, the authors claim "Using k-fold cross-validations (k = 4), the random forest model identified and utilized the most discriminative features with 100.00% ± 0.0% (mean ± SD) accuracy in the prediction of twelve colors (Figure 2A)." However, Figure 2A seems to be a heatmap across different colors, instead of prediction accuracy. Even if the authors refer to Figure 2B, it does not make any sense to me. What is the meaning of colors in Figure 2B? Is it the result for only one odor? I assume the "Momentum" and "Learning rate" are the parameters used in DBN, then where are the results using random forest? Where is the result for each fold in their 4-fold cross-validation? It is the same situation for odor prediction in Figure 3.
In sum, the authors really need solid evidence (e.g. shown in both box plots and supplementary tables) to support their claim of 100% and 89% accuracy in predicting color and odor.

Response: Thanks so much for your constructive comments and suggestions for our study. We strengthened and completed the key results and information for both color and odor prediction. All the figure legends were modified (Pages 21-22). We hope you find that we have addressed your concerns well.
To show our key results more clearly, we added boxplots presenting the results of color and odor prediction using the random forest or DBN (Figure 2C, D; Figure 3C, D). At the same time, confusion matrixes were used to assist in observing the prediction results achieved using random forest (Figure 2A; Figure 3A), and column charts were used to assist in observing the prediction results achieved with DBN to represent the results of predicting all twelve colors or all twelve odors (Figure 2B; Figure 3B). In addition, the color of the column has been changed to be uniform.
The updated results for each fold in the 4-fold cross-validation are shown below. The table has been added to the Supplementary Materials (Table S3).

Comment (2): The experiment details are not clearly described. Based on the manuscript, the authors first used a strategy called SMOTE to over-sample the minority class and under-sample the majority class. Then they performed 4-fold cross validation. This may introduce overfitting to their study. For example, a molecule was oversampled and used twice in both model training and model testing during their cross validations. The correct way is partitioning the data into the training and testing data first, then oversampling. The authors need to clarify this.

Response: Thanks so much for your scrupulous correction. We agree that partitioning the data into training and testing data should come first, followed by oversampling. In the previous version, first we separated the data used for the 4-fold cross-validation and testing, and second, we completed the oversampling for training. The test data were not oversampled. In this version, we did not use any oversampling method according to your suggestion, and we clarified this point on Page 7, Lines 145-

146.

Comment (3): The advantage of SMOTE is not clear. I suggest they compare the results of (1) SMOTE oversampling and (2) random oversampling.

Response: Thanks for your suggestion. We agree that the use of SMOTE oversampling needs further verification. In the review process, our results suggested that the accuracies achieved using direct classifications for the random forest and DBN (Table S3) were better than those achieved using SMOTE or random oversampling. Therefore, all the information about SMOTE oversampling has been removed.

Comment (4): The recent state-of-the-art method published in GigaScience ("Accurate prediction of personalized olfactory perception from large-scale chemoinformatic features.") was not discussed in this study. The author should compare with the previous method, or at least discuss the connections and differences between these studies.

Response: We are grateful for your recommendation. We have studied the best algorithm for olfaction prediction in the DREAM challenge and further discussed the connections and differences between the findings (Page 10, Lines 215-232).

Comment (5): The network architecture of deep belief network should be provided, including details such as number of layers, number of parameters.

Response: Many thanks for your comment. We compared three DBN structures for the prediction of either color or odor, and optimizations of the parameters of each structure have been conducted. The architecture that performed best for both color and odor prediction was the input layer with 5270 neurons and only one RBM with 5270 visible neurons and 500 hidden neurons. The moderate performance was achieved with the input layer with 5270 neurons and two RBMs. One RBM was composed of 5270 visible neurons and 2000 hidden neurons, and the other contained 2000 visible neurons and 500 hidden neurons. The worst performance was achieved with the input layer with 5270 neurons and three RBMs. One RBM contained 5270 visible neurons and 2000 hidden neurons, one was composed of 2000 visible neurons and 1000 hidden neurons, and the last contained 1000 visible neurons and 500 hidden neurons. Therefore, the best architecture was used in the follow-up prediction. We have also added these details on Page 13, Lines 284-295.

Reviewer #2:

Comment (1): The authors in their manuscript develop machine learning (random forest and DBN) trained models for distinguishing 12 distinct colours and 12 odours based on large-scale physicochemical features of 1267 and 598 structurally diverse molecules, respectively. In this analysis, the authors discuss identified important features for a specific classification. Moreover, shows some connections between colours, and odour features. The manuscript is well written, made it easier to go through the content. However, some major issues are listed below should discuss or clarify in the manuscript.

Response: Thanks for your agreement on the merit and quality of our work. We also appreciate your constructive comments and have further discussed the major restrictions of our study (see the following responses).

Comment (2): In the data description section: line 90 - 99: the decision of selecting these specific colours and odour is missing. For example where these colour for particular molecules previously defined from NCBI or they visually identify the colours of the molecules or they used some software for this identification. The similar question arises for the odours. I think odours are very subjective to the person who is labelling the features. This should be mentioned in the data description.

Response: Thanks so much for your constructive suggestion. We agree that olfactory perception varies greatly among individuals. So we selected molecules with definite color or odors as defined by the NCBI. A previous study of personalized olfactory perception published in GigaScience used a dataset of molecules sensed by 49 voluntary people [1]. They found that the perceived attributes including the intensity were rated differently among individuals, which considerably complicated the prediction challenge. We further discussed the connections and differences between these studies and emphasized the data from NCBI as our “gold standard” in the revised manuscript (Page 10, Lines 215-221).
1. Hongyang Li, Bharat Panwar, Gilbert S. Omenn & Yuanfang Guan. Accurate prediction of personalized olfactory perception from large-scale chemoinformatic features. GigaScience 2017; 7, 1–11.

Comment (3): line 111: replacing "NaN" with 0. I don't think the missing values should be treated this genitally. Unless all the missing values are because of one reason and the information is not needed for a particular molecule. The missing values in chemoinformatics dataset could be present because of various reasons, for example, the introduction of missing values is either no information was available (in literature/experiment etc) or due to the chemical calculation is not needed for this molecule. Both the cases can't have the same output. This should be reflected in your dataset and influence the model prediction. Also, mention how much missing data is present in your dataset.

Response: Thanks for your suggestion. In our study, all the missing values are due to unavailable information in Dragon 7.0, which is the most used application for the calculation of molecular descriptors worldwide. The missing values simply mean that for the associated molecules, some descriptors have not been calculated for some reason, which commonly happens, as several descriptors have particular constraints (https://chm.kode-solutions.net/products_dragon_tutorial.php#01). In addition, our results of classification were quite good when substituting "NaN" with 0, indicating that these missing values did not play significant roles in the prediction modeling. However, we agree that new information is required to confirm our findings if an upgraded version of the Dragon software becomes available. The reasons behind and statistics of missing data have been added in the data description according to your suggestion (Page 7, Lines 138-145).

Comment (4): In Figure 1: it is unclear how odour dataset was included? Do you have two different workflows for colour and odour dataset?

Response: Thanks for your suggestion. We have rearranged Figure 1 to integrate the workflows of color and odor prediction.

Comment (5): I think the colour classification model is overestimating the prediction of the training dataset. For a clear understanding, you can report sensitivity, specificity, and F1 instead of accuracy, also because of accuracy paradox.

Response: Many thanks for your comment. We are sorry that the sensitivity, specificity, and F1 which are regularly used for model evaluation in bi-classification were not fit for our study. Because both color and odor were divided into twelve categories, confusion matrixes were used to assist in observing the prediction effects of the random forest (Figure 2A, 3A), and column charts were used to assist in observing the prediction effects of the DBN (Figure 2B, 3B). To better evaluate our models of twelve-category classification, we added the kappa coefficient. Upon using all features to predict color, $k = 1.0000 \pm 0.0000$ (mean ± SD) using the random forest, and $k = 0.9400 \pm 0.0030$ (mean ± SD) using the DBN. Upon using all features to predict odor, $k = 0.9232 \pm 0.0037$ (mean ± SD) using the random forest, and $k = 0.9397 \pm 0.0031$ (mean ± SD) using the DBN. The kappa coefficients have been added in the results (Page7, Lines 152-153; Page8, Lines 177-178).

Comment (6): Figure legend is missing, which makes it hard to read and understand the figures.

Response: Thanks so much for your correction. All the figure legends were modified (Pages 21-22).

Comment (7): From figures and text, it is unclear if the random forest performed better than DBN? This is not the main findings of this manuscript, however, it is helpful to identify which method performs better for future prediction. The impression of Figure 1 also suggests that there will be a comparison between the random forest and DBN. The comparison, in terms of evaluation measure (false positives, false negative, F1 measure), should be mentioned in the main publication.

Response: Thanks for your suggestion. We agree that the comparison between the random forest and DBN should be completed. To show our key results more clearly, we added boxplots presenting the results of the random forest and DBN for color and odor prediction (Figure 2C, D; Figure 3C, D). The new results for each fold in the 4-fold cross-validation are shown below. The table has also been added to the Supplementary Materials (Table S3).
Overall, we found that the accuracy and kappa coefficient achieved using the random forest (100% ± 0.00%, 1.0000 ± 0.0000) were better than those achieved with the DBN (95.23% ± 0.40%, 0.9400 ± 0.0030) in color prediction with twelve categories. For odor prediction with twelve categories, the accuracy and kappa coefficient achieved using the DBN (94.75% ± 0.44%, 0.9397 ± 0.0031) were better than those achieved with the random forest (93.40% ± 0.31%, 0.9232 ± 0.0037). We further discussed the comparison in Page 10, Line 202-209. We are sorry that the sensitivity, specificity, and F1

which are regularly used for model evaluation in bi-classification, were not fit for our study.

Comment (8): Line 218, could you elaborate on how random forest can effectively avoid overfitting and deliver generalized knowledge? there is no evidence suggesting that random forest avoids overfitting. For some reference check this blog: https://mljar.com/blog/random-forest-overfitting/.

Response: Thanks for your scrupulous correction. We removed the statement "A random forest model can effectively avoid overfitting" to avoid potential controversies in the Method (Page 12, Lines 260-261).

Comment (9): The random forest can produce variable importance, out of curiosity, are the variable importance comparable to the genetic algorithm? I think this is an interesting part of your publication that can be discussed.

Response: Thanks for your suggestion. We agree that the comparison between the random forest and genetic algorithm could be very interesting. However, the dimensionality of the physicochemical data was very high with 5270 descriptors per molecule, and the data matrix was sparse in our study (Page 14, Lines 297-300). Many of the features are valued as "0" when calculated by the Dragon software, which means that they do not contribute to the classification (Page 14, Lines 304-306). Therefore, we preferred to combine the genetic algorithm and random forest algorithm, while the genetic algorithm was used for feature selection.

Comment (10): Change heat map to heatmap in the publication.

Response: Thanks for your suggestion. We have changed the statement accordingly (Lines 164, 184).

Comment (11): Use the full form first before abbreviation.

Response: Thanks for your suggestion. We have made modifications to use the full form first. A list of abbreviations is presented on Page 16, Line 342-345.

Finally, thank you again for your acceptance and all of the helpful comments, and we hope that you will now find our revisions suitable for publication.

Sincerely yours,
Haotian Lin on behalf of all authors

Close
