# Supplementary material for: Artificial intelligence deciphers codes for color and odor perceptions based on large-scale chemoinformatic data
Source: Gigascience. 2020 Feb 26;9(2):giaa011. doi: 10.1093/gigascience/giaa011 (PMC7043059; doi:10.1093/gigascience/giaa011)
Supplement: giaa011_Response_to_Reviewer_Comments_Revision_1 [file giaa011_response_to_reviewer_comments_revision_1.pdf]

Author's Response To Reviewer Comments

Close

Dear Scott Edmunds and Reviewers,

Thank you so much for the agreements and insightful suggestions on our manuscript.

The following are our point-by-point responses to the reviewers' comments and corresponding changes are marked in the revised manuscript. We hope that we have addressed all the suggestions adequately.

Our point-by-point responses are as follows:

Reviewer #1:

Comment (1): Thank you for revising and improving the manuscript. The authors have addressed my previous concerns. I still have a few comments about the revised manuscript: In Figure 3A, it seems the model cannot distinguish "sweet" and "unpleasant" very well. But in general, "sweet" should be a "pleasant" odor instead of an "unpleasant" one. How can these results be reconciled?

Response: Thanks so much for your agreement on the merit of our work, as well as your constructive comments. We considered olfactory perception vary greatly among individuals, as your study discovered that the perceived attributes were rated differently among individuals, which considerably complicated the prediction challenge (GigaScience 2017; 7, 1–11). So in this study we emphasized the selection of molecules with definite color or odors were defined by NCBI (Line 103-104, 210-212). If the molecule is defined as "sweet" in NCBI, we try not to classify this "sweet" as a "pleasant sweet" or an "unpleasant sweet" according to our subjective feelings. And molecules with multiple odors that are difficult to define by NCBI were excluded.
We certainly agree that this process may reduce the credibility of results in the real world, and bring about the results that odor sensing was less accurate than that of color in our study (Discussion, Line 217-221). However, rather than arranging large-scale human resources from different races to distinguish odors "fairly", we prefer this way to deal with the data.

Comment (2): For Figure 2C-D and Figure 3C-D, it would be clearer if the median values of these boxplots are labeled.

Response: Thank you again for your acceptance and all of the helpful comments. We have labeled the median values in the boxplots in Figure 2C-D and Figure 3C-D. The figure legends have been modified correspondingly (Line 457-465, 474-483).

Reviewer #2:

Comment (1): Figure 1: from the figure itself it looks like that the feature selection was done separately. However, to test the performance of the classifiers based on feature selected by the genetic algorithm random forest and DBN model were used again. This is unclear for this figure but clear in the text.

Response: Thanks so much for your constructive comments and suggestions for our study. We have rearranged Figure 1 to show the random forest and DBN models were used for a second time after feature selection. The figure legend has been modified correspondingly (Line 446-449).

Comment (2): Usually, a feature selection step is performed before model building and random forest can perform this selection on its own. I am not convinced with the response of the author that due to the sparsity of the matrix and huge physicochemical data they did not discuss the feature importance results from random forest however they consider random forest performs the best. This aspect should be discussed in the publication.

Response: We really appreciate your constructive suggestions. We have revised our method applying random forest models to perform feature selection on its own, and found that random forest alone did even better than combining it with the genetic algorithm using the same number of features (24 features for color perception, 39 features for odor perception). The results for each fold in the 4-fold cross-validation are shown below comparing two methods of feature selection after our correction, and the best performing results we used in the revised results are highlighted.

Table S3. The results for each fold in the 4-fold cross-validation.
Task Method Number of features Mean accuracy Accuracy (4-fold cross-validation)
Color perception Random forest ① All features 100% 100%, 100%, 100%, 100%
② 24 features # 100% 100%, 100%, 100%, 100%
③ 24 features * 99.45% 99.37%, 99.69%, 99.36%, 99.37%
② + ③features 100% 100%, 100%, 100%, 100%
DBN ① All features 95.23% 95.89%, 94.93%, 94.88%, 95.23%
② 24 features # 47.35% 47.95%, 49.06%, 46.50%, 45.89%
③ 24 features * 44.20% 44.16%, 43.75%, 44.59%, 44.30%
② + ③features 48.79% 53.63%, 44.69%, 50.00%, 46.84%
Odor perception Random forest ① All features 93.40% 93.33%, 93.92%, 93.20%, 93.15%
② 39 features # 93.40% 93.33%, 93.92%, 93.20%, 93.15%
③ 39 features * 93.38% 93.33%, 93.90%, 93.18%, 93.11%
② + ③features 93.40% 93.33%, 93.92%, 93.20%, 93.15%
DBN ① All features 94.75% 95.24%, 94.35%, 94.27%, 95.13%
② 39 features # 36.18% 43.33%, 30.41%, 36.73%, 34.25%
③ 39 features * 31.46% 31.29%, 31.33%, 29.00%, 34.25%
② + ③features 42.44% 48.00%, 38.51%, 48.30%, 34.93%
Random forest models
* The combination of random forest models and genetic algorithm

According to the new features selected by random forest models, we have made changes in the results section (Line 148-150, Line 166-168, Figure 2C-E, Figure 3C-E, Figure 4B and Table S1-S3). In the method section, we described the application of the two methods in detail (Line 280-299).

Comment (3): The results of the random forest are very impressive and confusing at the same time. I am unsure how can random forest achieve 100% accuracy with a sparse dataset as you mentioned in the comment for 12 different colours class. Because random forest -- bagging and suboptimal selection of splits may waste most of the model insight on zero-only areas. It will be very informative if you could show AUC for each class individually.

Response: Thanks for your suggestion. The AUCs of the random forest in the prediction of twelve colors and twelve odors using all features are shown below. The figure has also been added to the Supplementary Materials (Figure S1).

Table S4. The prediction accuracies of random forest models for twelve colors and twelve odors using all features. AUC, area under the curve.
Task Label AUC Task Label AUC
Color perception White 0.9982 Odor perception Ammonia 0.9985
Yellow 0.9991 Aromatic 0.9985
Colorless 0.9988 Characteristic 0.9957
Red 0.9994 Flowery 0.9963
Amber 0.9988 Fruity 0.9985
Dark gray 1.0000 Mild 0.9939
Brown 0.9983 Other 0.9982
Orange 0.9988 Odorless 0.9960
Purple 0.9968 Sweet 0.9654
Blue 0.9988 Spicy 0.9952
Green 0.9987 Pleasant 0.9987
Black 0.9988 Unpleasant 0.9607
We confirmed the earlier result based on a re-examination of the source code (https://github.com/Hugo0512/ColorOdorprediction).

Comment (4): line 145: "The data was divided into the training and testing data sets without oversampling" - could mention the proportion of your data split into training and testing?

Response: Many thanks for your comment. We re-emphasized the classification method of training and testing data sets was k-fold cross-validations (k = 4) in this line (Line 131-132).

Comment (5): line 194-195: Pearson correlation coefficients > 0.1958, please write the exact coefficient value.

Response: Thanks so much for your scrupulous correction. The minimum absolute value of the Pearson correlation coefficients applied in this revised vision was 0.300552 (Line 180-181).

Comment (6): Figure 4: A) the intersecting part of the pie chart can be a different colour. Before Figure 4, there is no explanation of vital features. It will be useful to include the definition of the vital feature before or while writing the result on the correlation between colour and odour.

Response: Thanks so much for your scrupulous correction. The color in Figure 4A has been modified. We also unified our expression using "key physicochemical features" (Line 92, 94, 148, 167, 195, 488, Figure 4B), and defined it as the physicochemical descriptors contributed most to the predictive accuracies (Line 90-93, 301-303).

Comment (7): Line 209-210: It will be helpful for the readers if the authors discuss the rationale behind the connected colour and odour physicochemical features.

Response: Thanks for your suggestion. The shared and prominently interlinked key physicochemical features identified in predicting color and odor have not been reported yet. We cautiously discussed the significance in Page 10, Line 199-202.

Comment (8): Line 215: it would be interesting to show the evaluation scores from the DREAM challenge participants to directly compare the results from your claim.

Response: Thanks for your suggestion. We agree that the evaluation scores from the DREAM challenge participants should be mentioned in the discussion (Line 203-205). Actually, there were great differences in data types and evaluation criteria between our study and the DREAM challenge. We solved one question with a multi category for each time here, while multiple questions are classified together in the DREAM challenge. The evaluation scores we used were accuracies, kappa coefficients and AUCs, the evaluation in DREAM challenge was based on the Pearson's correlation between observed and predicted perceptions.

Comment (9): line 221, is a redundant sentence instead you can write - "The winning algorithm of the DREAM challenge indicated that the random forest outperforms other base learning methods. "

Response: Thanks so much for your scrupulous correction. We removed the statement accordingly in Line 208-210.

Comment (10): line 228 no comma needed.

Response: Thanks so much for your scrupulous correction. The sentence has been removed in the revised version.

Comment (11): line 461: and is missing in this line - "all features and the top 24 features".

Response: Thanks so much for your scrupulous correction. We have modified the figure legends (Line 457-465).

Comment (12): line 202, as a reader it would be informative to get the definition of terse framework or at least a citation?

Response: Thanks so much for your scrupulous correction. We removed the word "terse" to avoid misunderstanding (Line 188).

Comment (13): The cutoff to decide the number of features is not clear in the manuscript.

Response: Many thanks for your comment. In order to compare random forest algorithm itself with the

combination of the genetic algorithm and random forest algorithm, the numbers of the key features selected were similar. As random forest model could not choose the cut off by itself, the cutoff to decide the number of features was determined by the genetic feature selection task. After running the genetic feature selection task 20 times, 24 descriptors were selected 18 times with a classification accuracy of 100% ± 0.0% in the random forest model for color, 39 descriptors were selected 16 times with a classification accuracy of 93.38% ± 0.31% in the random forest model for odor. (Method, Line 287-291, 296-299).

Finally, thank you again for your acceptance and all of the helpful comments, and we hope that you will now find our revisions suitable for publication.

Sincerely yours,
Haotian Lin on behalf of all authors

Close
