# Supplementary material for: Artificial intelligence deciphers codes for color and odor perceptions based on large-scale chemoinformatic data
Source: Gigascience. 2020 Feb 26;9(2):giaa011. doi: 10.1093/gigascience/giaa011 (PMC7043059; doi:10.1093/gigascience/giaa011)
Supplement: giaa011_Response_to_Reviewer_Comments_Revision_2 [file giaa011_response_to_reviewer_comments_revision_2.pdf]

Author's Response To Reviewer Comments

Close

Dear Edmunds and Reviewers,

Thank you so much for your agreement and clear corrections on our manuscript.
The following are our point-by-point responses to the reviewer's comments and corresponding changes are marked in the revised manuscript. We hope that we have addressed your concern adequately.

Our point-by-point responses are as follows:

Reviewer #2: Comment (1): Thank you for the second revision. It is quite impressive to see prediction accuracies of random forest models for predicting individual colours and odours. It is clear from your results (Table S3) that DBN might choose different sets of features in order to perform better because DBN can reach to 95% accuracy but fails to deliver best performance for 24 features chosen by random forest/genetic algorithm. Can you please make a short discussion on this point in your manuscript. This will reflect on the poor performance of DBN.
Response: Thanks so much for your insightful suggestions on our manuscript all along.
We considered that there were two main reasons why DBN failed to deliver best performance with the 24 features. Firstly, the key physicochemical features identified in predicting color and odor were selected by random forest and genetic algorithm. These features may not be suitable for DBN,which can map the raw data to low-dimensional space by unsupervised learning in the pre-training phase. Secondly, although DBN is a multi-layered recurrent neural network trained with energy minimizing methods, the network structure of DBN has a great influence on the learning performance. The network structure of DBN utilizing all features and the key physicochemical features are exactly the same in our study. If other algorithm like the Particle Swarm Optimization Algorithm could be used to optimize the number of DBN hidden-layer nodes, the performance of DBN network may improve. We have further discussed these potential reasons for the performance of DBN in Line 211-222.

Comment (2): line 115. specify the supplementary data table number.
Response: Thank you for your scrupulous correction. "Supplementary Data1-3" has been added in Line 114.

Comment (3): line 145-148 is not clear. As you stated the sentence saying random forest algorithm and the combination of the genetic algorithm and random forest algorithm..., where the second part of the sentence explains the advantage of random forest. This leaves the first part incomplete.
Response: We really appreciate your constructive suggestions. To highlight the best classification accuracy with 24 key physicochemical features selected by random forest in the results section, we have removed the description of feature selection methods here and revised the sentence in the method section (Line 288-290).

Comment (4): line 195-196. the sentence is misleading to suggest that physicochemical features are connected. This needs more clarity.
Response: Many thanks for your critical suggestion. We modified this sentence to "Our findings also suggested that key physicochemical features in distinguishing color and odor are significantly correlated" (Line 192-193).

Comment (5): line 199-202, this sentence is also misleading because the outcome of the edges between the two features is not explained properly. One can see that due to the correlation there are two features connected but there is no evidence suggesting that they influence changing the odour and colour.
Response: Thanks for your suggestion. We have changed the statement in a more conservative manner (Line 197-199).

Comment (6): There are some places where you mention random forest algorithm and the combination of the genetic algorithm and random forest algorithm. this is very confusing. I think you want to say that for feature selection random forest and genetic algorithm were used. Can you please make this consistent.
Response: Thank you again for your helpful corrections. We have made modifications to unify the descriptions for feature selection (Line 91, Line 287, Line 454, Line 467).

Finally, thank you again for all of the helpful comments, and we hope that you will now find our revisions suitable for publication.

Sincerely yours,
Haotian Lin on behalf of all authors

Close
