# Supplementary material for: Artificial intelligence deciphers codes for color and odor perceptions based on large-scale chemoinformatic data
Source: Gigascience. 2020 Feb 26;9(2):giaa011. doi: 10.1093/gigascience/giaa011 (PMC7043059; doi:10.1093/gigascience/giaa011)
Supplement: giaa011_Reviewer_1_Report_Original_Submission — Hongyang Li -- 6/22/2019 Reviewed [file giaa011_reviewer_1_report_original_submission.pdf]

Reviewer Report

Title: Artificial intelligence deciphers codes for color and odor perceptions based on large-scale chemoinformatic data

Version: Original Submission **Date:** 6/22/2019

Reviewer name: Hongyang Li

Reviewer Comments to Author:

The authors proposed machine learning approaches for predicting color and odor of a small molecule based on large-scale chemoinformatic features. They investigated the interplay between color and odor perception and found chemoinformatic features in predicting color and odor perception.
Major Comments
1. Key results and information are missing in this manuscript. None of the figure legends were provided. For example, in line 118, the authors claim "Using k-fold cross-validations (k = 4), the random forest model identified and utilized the most discriminative features with 100.00% Â± 0.0% (mean Â± SD) accuracy in the prediction of twelve colors (Figure 2A)." However, Figure 2A seems to be a heatmap across different colors, instead of prediction accuracy. Even if the authors refer to Figure 2B, it does not make any sense to me. What is the meaning of colors in Figure 2B? Is it the result for only one odor? I assume the "Momentum" and "Learning rate" are the parameters used in DBN, then where are the results using random forest? Where is the result for each fold in their 4-fold cross-validation?
It is the same situation for odor prediction in Figure 3.
In sum, the authors really need solid evidence (e.g. shown in both box plots and supplementary tables) to support their claim of 100% and 89% accuracy in predicting color and odor.
2. The experiment details are not clearly described. Based on the manuscript, the authors first used a strategy called SMOTE to over-sample the minority class and under-sample the majority class. Then they performed 4-fold cross validation. This may introduce overfitting to their study. For example, a molecule was oversampled and used twice in both model training and model testing during their cross validations. The correct way is partitioning the data into the training and testing data first, then oversampling. The authors need to clarify this.
3. The advantage of SMOTE is not clear. I suggest they compare the results of (1) SMOTE oversampling and (2) random oversampling.
4. The recent state-of-the-art method published in GigaScience ("Accurate prediction of personalized olfactory perception from large-scale chemoinformatic features.") was not discussed in this study. The author should compare with the previous method, or at least discuss the connections and differences between these studies.
5. The network architecture of deep belief network should be provided, including details such as number of layers, number of parameters.

Methods

Are the methods appropriate to the aims of the study, are they well described, and are necessary controls included? Choose an item.

Conclusions

Are the conclusions adequately supported by the data shown? Choose an item.

Reporting Standards

Does the manuscript adhere to the journal's guidelines on minimum standards of reporting? Choose an item.

Choose an item.

Statistics

Are you able to assess all statistics in the manuscript, including the appropriateness of statistical tests used? Choose an item.

Quality of Written English

Please indicate the quality of language in the manuscript: Choose an item.

Declaration of Competing Interests

Please complete a declaration of competing interests, considering the following questions:

- Have you in the past five years received reimbursements, fees, funding, or salary from an organisation that may in any way gain or lose financially from the publication of this manuscript, either now or in the future?
- Do you hold any stocks or shares in an organisation that may in any way gain or lose financially from the publication of this manuscript, either now or in the future?
- Do you hold or are you currently applying for any patents relating to the content of the manuscript?
- Have you received reimbursements, fees, funding, or salary from an organization that holds or has applied for patents relating to the content of the manuscript?
- Do you have any other financial competing interests?
- Do you have any non-financial competing interests in relation to this paper?

If you can answer no to all of the above, write 'I declare that I have no competing interests' below. If your reply is yes to any, please give details below.

I declare that I have no competing interests

I agree to the open peer review policy of the journal. I understand that my name will be included on my report to the authors and, if the manuscript is accepted for publication, my named report including any attachments I upload will be posted on the website along with the authors' responses. I agree for my report to be made available under an Open Access Creative Commons CC-BY license (http://creativecommons.org/licenses/by/4.0/). I understand that any comments which I do not wish to be included in my named report can be included as confidential comments to the editors, which will not be published.

Choose an item.

To further support our reviewers, we have joined with Publons, where you can gain additional credit to further highlight your hard work (see: https://publons.com/journal/530/gigascience). On publication of this paper, your review will be automatically added to Publons, you can then choose whether or not to claim your Publons credit. I understand this statement.

Yes Choose an item.
