# Supplementary material for: Artificial intelligence deciphers codes for color and odor perceptions based on large-scale chemoinformatic data
Source: Gigascience. 2020 Feb 26;9(2):giaa011. doi: 10.1093/gigascience/giaa011 (PMC7043059; doi:10.1093/gigascience/giaa011)
Supplement: giaa011_Reviewer_2_Report_Original_Submission — Neetika Nath, Ph.D. -- 7/30/2019 Reviewed [file giaa011_reviewer_2_report_original_submission.pdf]

Reviewer Report

Title: Artificial intelligence deciphers codes for color and odor perceptions based on large-scale chemoinformatic data

Version: Original Submission **Date:** 7/30/2019

Reviewer name: Neetika Nath, Ph.D.

Reviewer Comments to Author:

The authors in their manuscript develop machine learning (random forest and DBN) trained models for distinguishing 12 distinct colours and 12 odours based on large-scale physicochemical features of 1267 and 598 structurally diverse molecules, respectively. In this analysis, the authors discuss identified important features for a specific classification. Moreover, shows some connections between colours, and odour features. The manuscript is well written, made it easier to go through the content. However, some major issues are listed below should discuss or clarify in the manuscript.

Comments:

1. In the data description section: line 90 - 99: the decision of selecting these specific colours and odour is missing. For example where these colour for particular molecules previously defined from NCBI or they visually identify the colours of the molecules or they used some software for this identification. The similar question arises for the odours. I think odours are very subjective to the person who is labelling the features. This should be mentioned in the data description.
2. line 111: replacing "NaN" with 0. I don't think the missing values should be treated this genitally. Unless all the missing values are because of one reason and the information is not needed for a particular molecule. The missing values in chemoinformatics dataset could be present because of various reasons, for example, the introduction of missing values are either no information was available (in literature/experiment etc) or due to the chemical calculation is not needed for this molecules. Both the cases cant have the same output. This should be reflected in your dataset and influence the model prediction. Also, mention how much missing data is present in your dataset.
3. In Figure 1: it is unclear how odour dataset was included? Do you have two different workflows for colour and odour dataset?
4. I think the colour classification model is overestimating the prediction of the training dataset. For a clear understanding, you can report sensitivity, specificity, and F1 instead of accuracy, also because of accuracy paradox.
5. Figure legend is missing, which makes it hard to read and understand the figures.
6. From figures and text, it is unclear if the random forest performed better than DBN? This is not the main findings of this manuscript, however, it is helpful to identify which method performs better for future prediction. The impression of Figure 1 also suggests that there will be a comparison between the random forest and DBN. The comparison, in terms of evaluation measure (false positives, false negative, F1 measure), should be mentioned in the main publication.
7. Line 218, could you elaborate on how random forest can effectively avoid overfitting and deliver generalized knowledge? there is no evidence suggesting that random forest avoids overfitting. For some

reference check this blog:https://mljar.com/blog/random-forest-overfitting/
8. The random forest can produce variable importance, out of curiosity, are the variable importance comparable to the genetic algorithm? I think this is an interesting part of your publication that can be discussed.
Minor comments:
1. change heat map to heatmap in the publication
2. Use the full form first before abbreviation.

Methods

Are the methods appropriate to the aims of the study, are they well described, and are necessary controls included? Choose an item.

Conclusions

Are the conclusions adequately supported by the data shown? Choose an item.

Reporting Standards

Does the manuscript adhere to the journal's guidelines on [minimum standards of reporting?] Choose an item.

Choose an item.

Statistics

Are you able to assess all statistics in the manuscript, including the appropriateness of statistical tests used? Choose an item.

Quality of Written English

Please indicate the quality of language in the manuscript: Choose an item.

Declaration of Competing Interests

Please complete a declaration of competing interests, considering the following questions:

- Have you in the past five years received reimbursements, fees, funding, or salary from an organisation that may in any way gain or lose financially from the publication of this manuscript, either now or in the future?
- Do you hold any stocks or shares in an organisation that may in any way gain or lose financially from the publication of this manuscript, either now or in the future?
- Do you hold or are you currently applying for any patents relating to the content of the manuscript?

- Have you received reimbursements, fees, funding, or salary from an organization that holds or has applied for patents relating to the content of the manuscript?
- Do you have any other financial competing interests?
- Do you have any non-financial competing interests in relation to this paper?

If you can answer no to all of the above, write 'I declare that I have no competing interests' below. If your reply is yes to any, please give details below.

I declare that I have no competing interests

I agree to the open peer review policy of the journal. I understand that my name will be included on my report to the authors and, if the manuscript is accepted for publication, my named report including any attachments I upload will be posted on the website along with the authors' responses. I agree for my report to be made available under an Open Access Creative Commons CC-BY license (http://creativecommons.org/licenses/by/4.0/). I understand that any comments which I do not wish to be included in my named report can be included as confidential comments to the editors, which will not be published.

Choose an item.

To further support our reviewers, we have joined with Publons, where you can gain additional credit to further highlight your hard work (see: https://publons.com/journal/530/gigascience). On publication of this paper, your review will be automatically added to Publons, you can then choose whether or not to claim your Publons credit. I understand this statement.

Yes Choose an item.
