# Supplementary material for: Artificial intelligence deciphers codes for color and odor perceptions based on large-scale chemoinformatic data
Source: Gigascience. 2020 Feb 26;9(2):giaa011. doi: 10.1093/gigascience/giaa011 (PMC7043059; doi:10.1093/gigascience/giaa011)
Supplement: giaa011_Reviewer_2_Report_Revision_1 — Neetika Nath, Ph.D. -- 11/28/2019 Reviewed [file giaa011_reviewer_2_report_revision_1.pdf]

Reviewer Report

Title: Artificial intelligence deciphers codes for color and odor perceptions based on large-scale chemoinformatic data

Version: Revision 1 **Date:** 11/28/2019

Reviewer name: Neetika Nath, Ph.D.

Reviewer Comments to Author:

Figure 1: from the figure itself it looks like that the feature selection was done separately. However, to test the performance of the classifiers based on feature selected by the genetic algorithm random forest and DBN model were used again. This is unclear for this figure but clear in the text. Usually, a feature selection step is performed before model building and random forest can perform this selection on its own. I am not convinced with the response of the author that due to the sparsity of the matrix and huge physicochemical data they did not discuss the feature importance results from random forest however they consider random forest performs the best. This aspect should be discussed in the publication.
The results of the random forest are very impressive and confusing at the same time. I am unsure how can random forest achieve 100% accuracy with a sparse dataset as you mentioned in the comment for 12 different colours class. Because random forest -- bagging and suboptimal selection of splits may waste most of the model insight on zero-only areas. It will be very informative if you could show AUC for each class individually.
line 145: "The data was divided into the training and testing data sets without oversampling" - could mention the proportion of your data split into training and testing?
line 194-195 Pearson correlation coefficients > 0.1958, please write the exact coefficient value.
Figure 4: A) the intersecting part of the pie chart can be a different colour. Before Figure 4, there is no explanation of vital features. It will be useful to include the definition of the vital feature before or while writing the result on the correlation between colour and odour.
Line 209-210: It will be helpful for the readers if the authors discuss the rationale behind the connected colour and odour physicochemical features.
Line 215: it would be interesting to show the evaluation scores from the DREAM challenge participants to directly compare the results from your claim.
line 221, is a redundant sentence instead you can write - "The winning algorithm of the DREAM challenge indicated that the random forest outperforms other base learning methods. "
line 228 no comma needed
line 461: and is missing in this line - "all features and the top 24 features".
line 202, as a reader it would be informative to get the definition of terse framework or at least a citation?
The cutoff to decide the number of features is not clear in the manuscript

Methods

Are the methods appropriate to the aims of the study, are they well described, and are necessary controls included? Choose an item.

Conclusions

Are the conclusions adequately supported by the data shown? Choose an item.

Reporting Standards

Does the manuscript adhere to the journal's guidelines on minimum standards of reporting? Choose an item.

Choose an item.

Statistics

Are you able to assess all statistics in the manuscript, including the appropriateness of statistical tests used? Choose an item.

Quality of Written English

Please indicate the quality of language in the manuscript: Choose an item.

Declaration of Competing Interests

Please complete a declaration of competing interests, considering the following questions:

- Have you in the past five years received reimbursements, fees, funding, or salary from an organisation that may in any way gain or lose financially from the publication of this manuscript, either now or in the future?
- Do you hold any stocks or shares in an organisation that may in any way gain or lose financially from the publication of this manuscript, either now or in the future?
- Do you hold or are you currently applying for any patents relating to the content of the manuscript?
- Have you received reimbursements, fees, funding, or salary from an organization that holds or has applied for patents relating to the content of the manuscript?
- Do you have any other financial competing interests?
- Do you have any non-financial competing interests in relation to this paper?

If you can answer no to all of the above, write 'I declare that I have no competing interests' below. If your reply is yes to any, please give details below.

I declare that I have no competing interests

I agree to the open peer review policy of the journal. I understand that my name will be included on my report to the authors and, if the manuscript is accepted for publication, my named report including any attachments I upload will be posted on the website along with the authors' responses. I agree for my

report to be made available under an Open Access Creative Commons CC-BY license (http://creativecommons.org/licenses/by/4.0/). I understand that any comments which I do not wish to be included in my named report can be included as confidential comments to the editors, which will not be published.

Choose an item.

To further support our reviewers, we have joined with Publons, where you can gain additional credit to further highlight your hard work (see: https://publons.com/journal/530/gigascience). On publication of this paper, your review will be automatically added to Publons, you can then choose whether or not to claim your Publons credit. I understand this statement.

Yes Choose an item.
