# Supplementary material for: Artificial intelligence deciphers codes for color and odor perceptions based on large-scale chemoinformatic data
Source: Gigascience. 2020 Feb 26;9(2):giaa011. doi: 10.1093/gigascience/giaa011 (PMC7043059; doi:10.1093/gigascience/giaa011)
Supplement: giaa011_Reviewer_2_Report_Revision_2 — Neetika Nath, Ph.D. -- 1/6/2020 Reviewed [file giaa011_reviewer_2_report_revision_2.pdf]

Reviewer Report

Title: Artificial intelligence deciphers codes for color and odor perceptions based on large-scale chemoinformatic data

Version: Revision 2 **Date:** 1/6/2020

Reviewer name: Neetika Nath, Ph.D.

Reviewer Comments to Author:

Thank you for the second revision. It is quite impressive to see prediction accuracies of random forest models for predicting individual colours and odours. It is clear from your results (Table S3) that DBN might choose different sets of features in order to perform better because DBN can reach to 95% accuracy but fails to deliver best performance for 24 features chosen by random forest/genetic algorithm. Can you please make a short discussion on this point in your manuscript. This will reflect on the poor performance of DBN.
line 115. specify the supplementary data table number.
line 145-148 is not clear. As you stated the sentence saying random forest algorithm and the combination of the genetic algorithm and random forest algorithm..., where the second part of the sentence explains the advantage of random forest. This leaves the first part incomplete.
line 195-196. the sentence is misleading to suggest that physicochemical features are connected. This needs more clarity. line 199-202, this sentence is also misleading because the outcome of the edges between the two features is not explained properly. One can see that due to the correlation there are two features connected but there is no evidence suggesting that they influence changing the odour and colour
there are some places where you mention random forest algorithm and the combination of the genetic algorithm and random forest algorithm. this is very confusing. I think you want to say that for feature selection random forest and genetic algorithm were used. Can you please make this consistent.

Methods

Are the methods appropriate to the aims of the study, are they well described, and are necessary controls included? Choose an item.

Conclusions

Are the conclusions adequately supported by the data shown? Choose an item.

Reporting Standards

Does the manuscript adhere to the journal's guidelines on minimum standards of reporting? Choose an item.

Choose an item.

Statistics

Are you able to assess all statistics in the manuscript, including the appropriateness of statistical tests used? Choose an item.

Quality of Written English

Please indicate the quality of language in the manuscript: Choose an item.

Declaration of Competing Interests

Please complete a declaration of competing interests, considering the following questions:

- Have you in the past five years received reimbursements, fees, funding, or salary from an organisation that may in any way gain or lose financially from the publication of this manuscript, either now or in the future?
- Do you hold any stocks or shares in an organisation that may in any way gain or lose financially from the publication of this manuscript, either now or in the future?
- Do you hold or are you currently applying for any patents relating to the content of the manuscript?
- Have you received reimbursements, fees, funding, or salary from an organization that holds or has applied for patents relating to the content of the manuscript?
- Do you have any other financial competing interests?
- Do you have any non-financial competing interests in relation to this paper?

If you can answer no to all of the above, write 'I declare that I have no competing interests' below. If your reply is yes to any, please give details below.

I declare that I have no competing interests

I agree to the open peer review policy of the journal. I understand that my name will be included on my report to the authors and, if the manuscript is accepted for publication, my named report including any attachments I upload will be posted on the website along with the authors' responses. I agree for my report to be made available under an Open Access Creative Commons CC-BY license (http://creativecommons.org/licenses/by/4.0/). I understand that any comments which I do not wish to be included in my named report can be included as confidential comments to the editors, which will not be published.

Choose an item.

To further support our reviewers, we have joined with Publons, where you can gain additional credit to further highlight your hard work (see: https://publons.com/journal/530/gigascience). On publication of this paper, your review will be automatically added to Publons, you can then choose whether or not to claim your Publons credit. I understand this statement.

Yes Choose an item.
